# Application of a mobile health data platform for public health surveillance: A case study in stress monitoring and prediction

**DOI:** 10.1177/20552076241249931

**Published:** 2024-06-08

**Authors:** Pedro Elkind Velmovitsky, Paulo Alencar, Scott T Leatherdale, Donald Cowan, Plinio Pelegrini Morita

**Affiliations:** 1 School of Public Health Sciences, 8430 University of Waterloo, Waterloo, ON, Canada; 2 David R. Cheriton School of Computer Science, 8430 University of Waterloo, Waterloo, ON, Canada; 3 Research Institute for Aging, 8430 University of Waterloo, Waterloo, ON, Canada; 4 Department of Systems Design Engineering, 8430 University of Waterloo, Waterloo, ON, Canada; 5 Institute of Health Policy, Management, and Evaluation, Dalla Lana School of Public Health, University of Toronto, Toronto, ON, Canada; 6 Centre for Digital Therapeutics, Techna Institute, University Health Network, Toronto, ON, Canada

**Keywords:** Public health, stress, machine learning, mHealth, app, heart rate, sleep, Apple Health

## Abstract

**Background:**

Public health surveillance involves the collection, analysis and dissemination of data to improve population health. The main sources of data for public health decision-making are surveys, typically comprised of self-report which may be subject to biases, costs and delays. To complement subjective data, objective measures from sensors could potentially be used. Specifically, advancements in personal mobile and wearable technologies enable the collection of real-time and continuous health data.

**Objective:**

In this context, the goal of this work is to apply a mobile health platform (MHP) that extracts health data from the Apple Health repository to collect data in daily-life scenarios and use it for the prediction of stress, a major public health issue.

**Methods:**

A pilot study was conducted with 45 participants over 2 weeks, using the MHP to collect stress-related data from Apple Health and perceived stress self-reports. Apple, Withings and Empatica devices were distributed to participants and collected a wide range of data, including heart rate, sleep, blood pressure, temperature, and weight. These were used to train random forests and support vector machines. The SMOTE technique was used to handle imbalanced datasets.

**Results:**

Accuracy and f1-macro scores were in line with state-of-the-art models for stress prediction above 60% for the majority of analyses and samples analysed. Apple Watch sleep features were particularly good predictors, with most models with these data achieving results around 70%.

**Conclusions:**

A system such as the MHP could be used for public health data collection, complementing traditional self-reporting methods when possible. The data collected with the system was promising for monitoring and predicting stress in a population.

## Introduction

The goal of public health is to improve and protect the health of communities and populations.^1^ In order to understand the characteristics of a population and where treatments and interventions are more effective, public health agencies typically conduct surveillance efforts to collect and analyse data.^2–4^ These efforts are traditionally focused on self-report, such as surveys and questionnaires. For example, the Canadian Health Measures Survey^5^ and the Canadian Community Health Survey^6^ are major surveys that collect data on the characteristics, behaviour and health of Canadians. However, subjective and self-reported data may be subject to limitations such as biases, delays, costs and logistics.^7–16^

New advancements in sensing and remote monitoring technologies allow the ubiquitous and effortless monitoring of objective health data with the use of smart devices and Internet of Things (IoT) solutions.^17^ For example, smartphones can typically collect movement data; smart thermostats are able to collect temperature and movements around the house; and smartwatches collect a range of variables from heart rate to steps and sleep.^17–19^ These technologies could potentially be used in complement to traditional data collection techniques, collecting objective data that can mitigate challenges associated with self-report – as evidenced by a number of recent studies that use mobile and wearable technologies to gain new insights into the health of individuals.^20–26^ Further, given the personal nature of these devices, it is possible to leverage data that is being passively collected in real-life environments for long periods. For instance, smartwatches typically collect heart rate and steps data from individuals wearing them throughout the day, without any action required on the user's part. This could provide new and large sources of continuous, real-world data collected with relatively low effort that will allow scientists to conduct novel health research.^5^

Indeed, many efforts are being put into place to create platforms that allow individuals to share their data for research. For example, the ecobee smart thermostat company has a program called Donate Your Data,^27,28^ which enables the anonymous sharing of device information with researchers.^29–31^ The Ubiquitous Health Technology Lab at the University of Waterloo has developed a web platform that enables the enrolment of personal Fitbit and ecobee devices for research: data from the devices is continuously collected once a day once enrolment is complete.^32^ The Digital Epidemiology and Population Health Laboratory (DEPtH Lab) at Western University have developed the Smart Platform, which allows researchers to engage with personal devices of patients.^33^

However, these mobile health datasets also bring new set of challenges. Larger datasets, generated at faster speeds than previous data collection efforts, with a variety of formats, structures, and even different data collection periods, require new methods of processing and analysing data. Therefore, to handle this Big Data, Machine Learning (ML) techniques have been shown to be useful tools in analysis, discovery and prediction.^34^

The goal of this study is to contribute to the informatisation of public health data collection efforts by introducing the mobile health platform (MHP), an iOS app that leverages data from personal devices that are stored in Apple Health (AH),^18,35^ one of the most popular health data repositories. We describe how this app was applied in a pilot study with 45 participants, collecting a plethora of health data (e.g. sleep, steps, heart rate, blood pressure) and using it to predict stress states through ML models, specifically random forests (RFs) and support vector machines (SVM). Related work on stress prediction and the rationale behind stress as a use case are presented in the next subsection.

### Related work – stress and machine learning

Stress is a major public health issue, with the World Health Organization calling it the ‘health epidemic of the 21^st^ century’,^36^ and its prevalence is increasing. While stress is a normal response to an unexpected situation – generating energy and enabling the individual to deal with a threatening circumstance – the body should, ideally, return to its normal state once the situation is resolved.^37,38^ Long-term, constant exposure to stressors can increase the risk for hypertension, cardiovascular diseases, and stroke, among others.^38,39^ It is estimated that stress places a burden of over $300 billion USD annually on health costs and job performance,^36,40^ leading to 120,000 preventable deaths when coupled with a lack of health insurance.^41^ The pandemic has also greatly affected the stress levels of individuals: according to a recent survey from the American Psychological Association, almost 70% of respondents experienced increased levels of stress due to COVID-19.^42^

Stress is typically collected for public health initiatives and in real-world environments through self-report.^38,43,44^ In this way, it is an ideal use case for the MHP, which collects objective data that can be used for stress prediction. Indeed, many studies have sought to use ML coupled with mobile and wearable technologies to predict stress. For example, a study used daily self-report for 4 months coupled with variables such as physical activity and heart rate variability (HRV) to predict stress in 35 participants.^45^ Logistic regression was used in a generalised model – using data from all participants and a leave-one-person-out (LOPO) validation method – with 53% accuracy, and an individualised model – using data from each participant using a leave-one-day-out validation procedure – with 61% accuracy. Jin et al.^46^ use data from 6 participants obtained with the Empatica E4 device such as blood volume pulse and electrodermal activity for 4 weeks, applying RFs and SVMs on a generalised model that uses 10-fold cross-validation to train and tune the model and a 10% validation set for testing, achieving an Area Under the Curve of 87.3% (RF) and 82.1% (SVM). Can et al.^47^ collects heart rate (HR) and electrodermal activity data from 14 participants, both in the lab and in real life during one week, training several combinations of models (e.g. models developed using the real-life data, laboratory data, or combination of both). Several ML algorithms are used on a generalised dataset. In particular, a 68% accuracy is achieved with SVMs and 52% accuracy with RF using 10-fold cross-validation and 80-20 train-test datasets for data collected in real-life.

In addition to daily self-report, many studies use stressors applied in a laboratory or controlled environment.^43,48,49^ Akmandor and Jha^49^ use ECG, respiration, blood pressure, and other variables from 32 participants to develop generalised and individualised models using SVMs and k-nearest neighbors (kNN), dividing the datasets into train, test and validation sets to validate the models. They achieved an accuracy of 89.2% (kNN) and 83.1% (SVM) for the generalised models and 94.5% (kNN) and 86.7% (SVM) for individualised ones. Liao et al.^50^ use neural networks to develop generalised models based on attention and meditation (as opposed to stress and non-stress states) in the laboratory with EEG data from 7 participants, achieving f1-scores of 60% for the attention state but of only 1% for the meditation state.

As can be seen by the examples above, the state-of-the-art accuracy for stress prediction seems to lie between 60% and 80%, decreasing for studies using real-life data. Further, there are many different ways to predict stress. Indeed, the works above vary widely according to the number of participants, period of data collection, algorithms used to develop the models, and metrics and methods applied to validate the models, among other factors. In this work, based on the results of previous studies and a survey by Can et al.,^38^ we elected to use RFs and SVMs, as they were successfully used in a variety of studies to predict stress. These models will be used to predict stress based on data collected from the MHP to test its efficacy in monitoring and predicting this condition in a population.

## Methods

### Recruitment and study protocol

We recruited participants from the University of Waterloo as well as through Facebook groups and Ads. Kijiji, a Canadian website that allows users to advertise products and services, was also used. Inclusion criteria consisted of participants aged 18 years and older, and initially involved participants that did not have any chronic condition, take any medication or consume alcohol/smoke frequently. The latter criteria were later relaxed due to a difficulty in finding participants, and this was accounted for in the analyses as will be expanded in the following subsections. Since devices were delivered to participants in-person, they were required to be located near the Kitchener–Waterloo region in Ontario.

Forty-five participants were recruited for the study and were offered CAD 100.00 for 2 weeks of data collection. Participants were given the following devices (per manufacturer):

- **Apple**: iPhone 8 with iOS 15.0 and Apple Watch Series 6 with watchOS 8.3.
- **Withings**: Withings Sleep, Withings Blood Pressure Monitor (BPM) Connect, Withings Thermos and Withings Body+
- **Empatica**: Empatica E4 wristband

Of this list, the Empatica E4 is the only one that is not considered a personal, consumer-level device, and was included due to its extensive use in stress prediction literature. During our experiments, we conducted analyses excluding Empatica data to test if this research-grade device had a significant impact on the results.

Table 1 describes the variables collected in each device. In Appendix 3, the user manual shared with each participant providing instructions on how devices should be installed and used is included. A 1-hour video call was also scheduled with each participant to go over the manual, make sure the devices were installed and working properly, and answer any questions about the protocol.

**Table 1.**
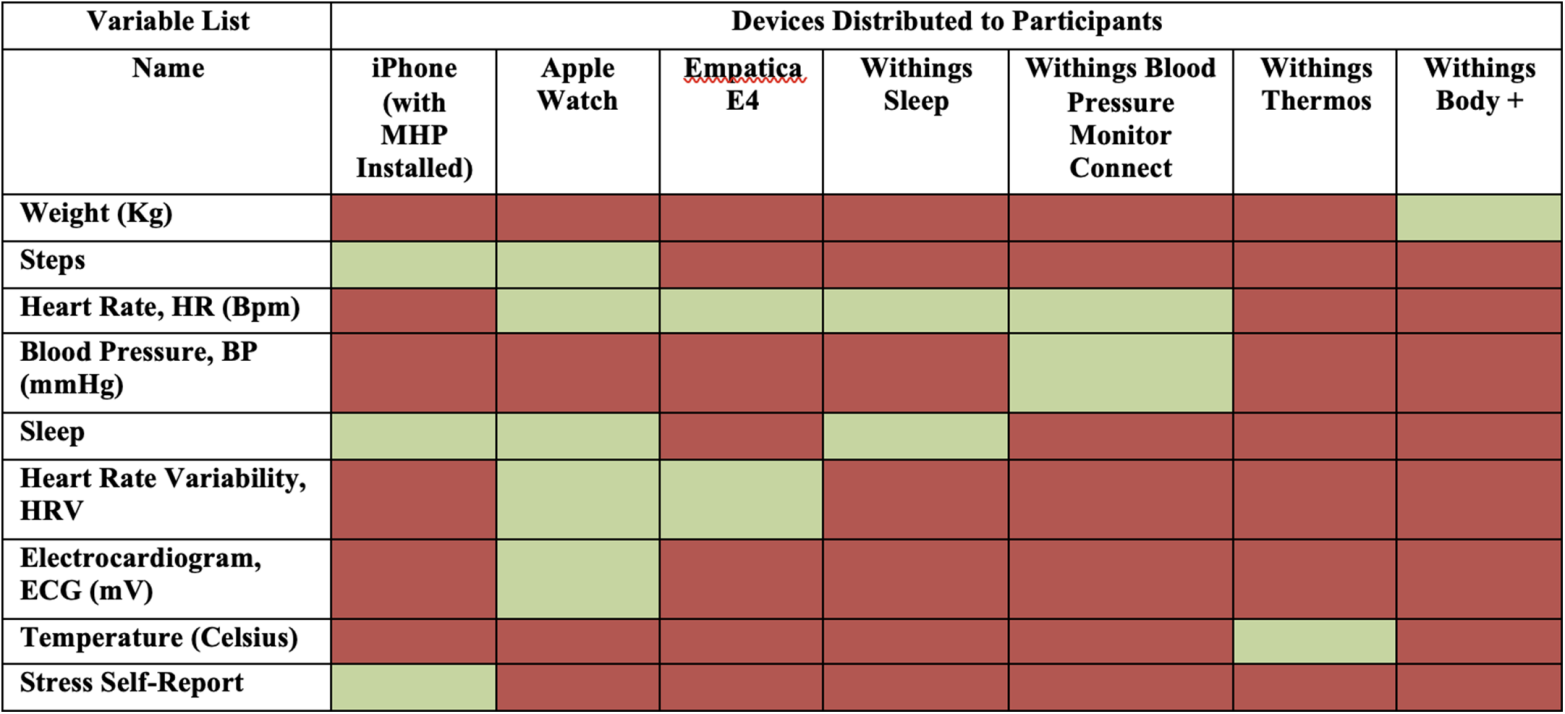
Variables collected and devices used in study.

**Figure 1.**
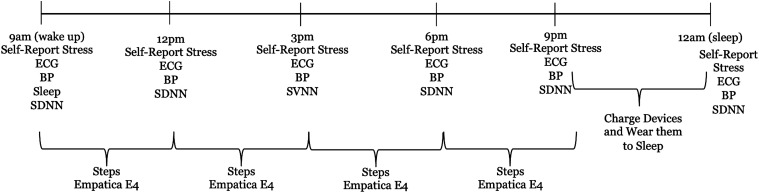
Study protocol.

This study followed the ecological momentary assessment (EMA) methodology, which strives to obtain self-reports closer to events in daily life to approximate real-world scenarios and obtain accurate data.^51^ Therefore, users were instructed to collect data 6 times during the day (starting at wake-up and finishing at sleep), in approximately 3-hour intervals according to their daily routine. This included taking Weight, Blood Pressure, Heart Rate Variability, ECG and Temperature measurements and filling out the stress self-report forms. Apple Watch and iPhone Steps, Apple Watch HR, and Empatica E4 data were collected continuously without patient involvement. The protocol is shown in Figure 1, with the times shown being illustrative.

The iPhone contained the prototype MHP. Participants were instructed on how to use the platform to complete stress self-reports (see User Manual in Appendix 3 for detailed instructions). The MHP uses the HealthKit Application Programming Interface (API) provided by Apple to extract Apple Health data.^52^

**Figure 2.**
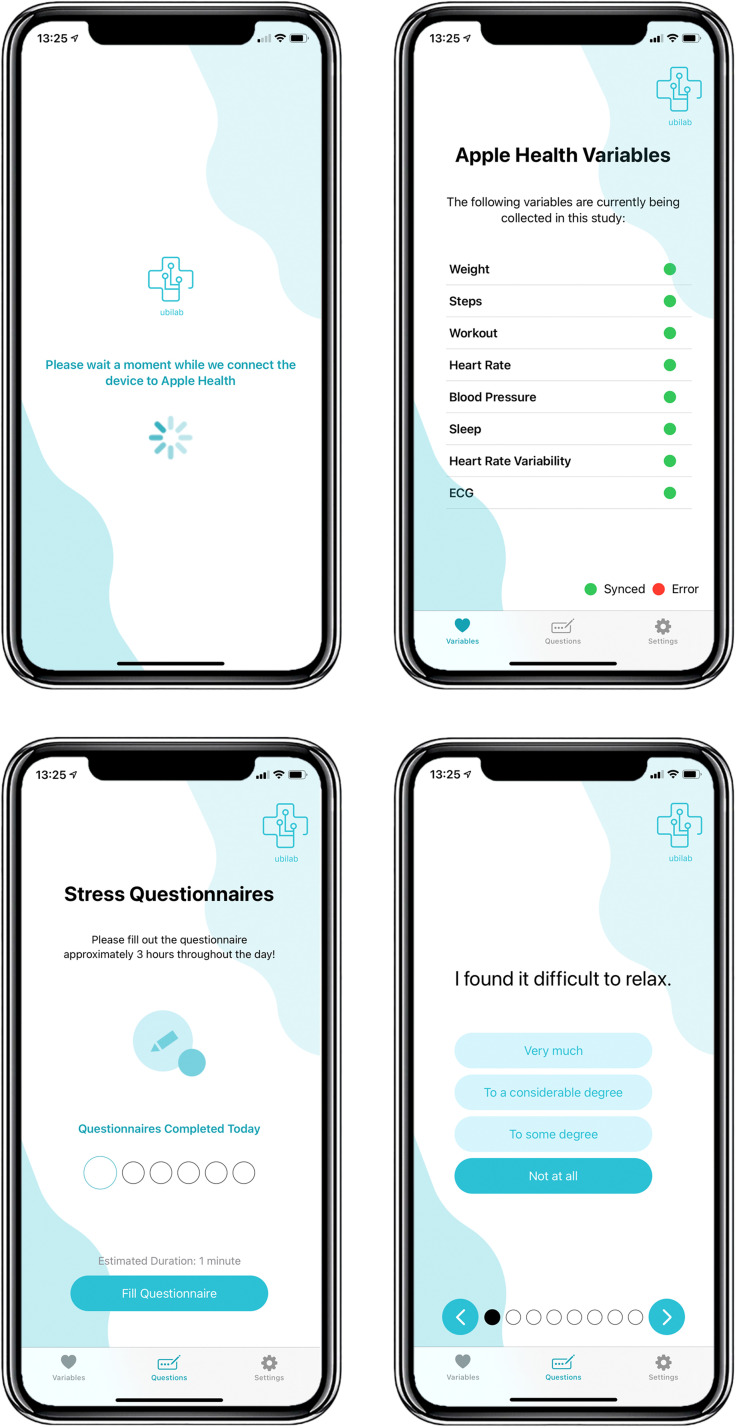
MHP interface.

Figure 2 shows the interface of the MHP, extracting Apple Health data automatically and allowing users to self-report their stress levels. The MHP is used as the data collection tool for the study: users open the app to answer the stress questionnaires (at which point all new Apple Health measurements are synced with the research database) and proceed to take the measurements, following the instructions in the User Manual in Appendix 3.

Notably, the HRV data on the Apple Watch is collected throughout the day based on user behaviour, but to trigger collection for our study, we used the Breathe app, an Apple Watch mindfulness application that asks users to breathe in and out for several minutes (more information in the User Manual).^53^ To avoid affecting stress levels, we asked users to do this as the last step in the data collection protocol.

In addition, while the Empatica E4 collects data continuously when active, we noticed that it constantly disconnected from the iPhone through its Bluetooth connection. Therefore, users were asked to constantly check if the Empatica was still active and, if not, to establish the connection again (see User Manual). However, as shall be described in further sections, this resulted in a lot of missing data from this device. Several participants encountered difficulties managing the study protocol with their daily life routines. In these cases, we asked participants to use the devices for additional days. This study was approved by the University Waterloo Research Ethics Board (REB [43612]). Data collection occurred between December 2021 and December 2022.

### Stress self-report

One of the challenges encountered when designing the study was a lack of validated stress questionnaires for EMA, as most stress questionnaires have a validated period of days or weeks. To mitigate this issue, we made use of the stress subscale of the Depression, Anxiety, and Stress Scale (DASS-21), comprised of seven questions related to stress. While the DASS-21 is usually applied over a week, there is promising evidence of using this questionnaire with EMA.^54^ In addition, Wang et al.^55^ used a single-item measure that, while lacking validation in the literature, was successfully applied for stress quantification and is moderately correlated with robust stress questionnaires.^55^

In our study, we used both questionnaires comprising the 8 questions that are asked to participants. Questions 1–7 are related to the DASS-21, and question 8 comprises the single-item measure used by Wang et al..^55^ The stress-related questions and respective questionnaires are shown in Table B1 in Appendix 2.

Following DASS-21 guidelines, the score is multiplied by 2 and, if it is above 14, we consider users as stressed in that moment.^56^ For the single-item measure, if the user answers ‘A little stressed’ or above, we consider users having stress. For each questionnaire filled, if the DASS-21 or the single-item measure (or both) show stress, that data collection period is marked as stress. In other words, if in the moment of data collection the DASS-21 score is marked as ‘stress’ and the single-item measure is marked as ‘no stress’, or vice versa, that data point will be labelled as ‘stress’. The questions are displayed to the user in a random order each time the questionnaire is filled. Figure A10 in Appendix 1 shows an example of the dataset.

### Data collection and pre-processing

For all variables and features, we evaluated if the participant had any data points missing. In the case of a missing data point, if that was an isolated event, that is if less than two data points were consecutively missed, we used the average between the next and previous data points for the participant. In case more than two data points were missing, that is two or more consecutive data points were missing, we used the k-nearest neighbors algorithm to estimate the value based on the proximity of features that are not missing. This was done using SciKit Learn's KNN Imputer method with number of neighboring samples set to 5.^57^ In addition, features were included if they had at least 30% of data for the specific user. This number was used to balance the amount of data used in the analyses while empirically assessing the KNNImputter behaviour and model performance for missing data.

Next, we describe the processing of variables extracted from each device in Table 1. An exhaustive list and description of all features used in the study are shown in Table B2 in Appendix 2.

#### Steps

For steps data, only Apple Watch information was used. Since data was collected through the HealthKit API differentiating between the Apple Watch and iPhone, it was not possible to integrate data from these two devices without avoiding duplication of information. From the Apple Watch steps data, we extracted the mean, maximum and minimum number of steps for the time interval between the start and end dates of the data point.

Unlike other data types, for the steps data we did not use averages or KNNImputer for missing data, as it was possible the user simply did not walk during the time period in question. Figure A11 in Appendix 1 shows example data points in the dataset for steps.

#### Heart rate

For HR data, we also focused on Apple Watch as the device collects data throughout the day over infrequent periods. The BPM Connect device only calculates HR when the participant is using the equipment, and Withings Sleep only collects data during the night. We calculated the mean, maximum and minimum heart rate for the time interval, measured as beats per minute.

In addition, we noticed that during the data collection protocol, when the user activates the Breathe or ECG apps, the device typically shortens the HR data collection period to milliseconds. Therefore, we also extracted a Short-Term HR feature in which we only consider the millisecond data close to the time the user filled out the stress self-report, rather than the entire HR data from the 3-hour time interval between the start and end dates of the data point. For the Short-Term features, we also extracted the mean, maximum and minimum. Finally, we used the data collected from the Apple Watch and for any other devices (e.g. Withings Sleep) during the night, that is after the last data point collected on day *t* - 1 and before the first data point on day *t*. Figure A12 shows a snapshot of the dataset with the Apple Watch HR features.

We also considered Empatica HR data. More specifically, we used the *BVP.csv* file supplied by Empatica, which provides raw data, to extract several HR and HRV features. This file was processed using the Kubios HRV Premium 3.5.0 software.^58^ More details on Kubios and HRV will be described in the next sub-section. For HR, Kubios provided the mean, maximum, minimum, and standard deviation of HR for the Empatica HR data during the time interval.

Finally, ECG data from the Apple Watch ECG app was also processed using Kubios. The HR output of ECG is similar to the Empatica, with the mean, maximum, minimum, and standard deviation of HR for the ECG data during the time interval

#### Heart rate variability/ECG

In AH, HRV is measured as the standard deviation of beat-to-beat measurements (SDNN). The Apple Watch measures SDNN using photoplethysmography (PPG), a technique in which a green LED light is used to detect the amount of blood flowing in the wrist,^59^ irregularly throughout the day. In addition to the passive collection of this metric with the Apple Watch, we also asked users to leave the Breathe app open for 5 minutes as the final step of the data collection process to trigger the HRV data collection, as previously mentioned. To process features, we calculated both the SDNN from the Breathe app and the SDNN collected throughout the day.

Despite not being able to control when the Apple Watch would collect the metric, we noticed that there was typically at least one SDNN data point collected per time interval. On the other hand, many users failed to activate the Breathe app during the data collection process, leading to missing data. Given that, in the real-life deployment of a system such as the MHP it is more feasible to depend on passively collected data, we decided to use the SDNN metric collected by the Apple Watch throughout the day rather than using the Breathe app as a trigger. This feature was named HRV-1 (see Table B2 in Appendix 2).

ECG data, composed of timestamps and voltage measurements which generate a 30-second measurement on the Apple Watch ECG app (see User Manual for more details), was processed using Kubios into HRV data. In terms of program parameters, Kubios automatic beat correction algorithm was used, and the automatic noise detection was set to medium.

It should be noted that there is limited evidence on the use of ultra-short HRV measurements^60,61^ such as the ones provided by the Apple Watch ECG app. To the best of our knowledge, this is one of the first works to use the Apple Watch ECG data in stress prediction. Following recommendations of the Task Force of The European Society of Cardiology and the North American Society of Pacing and Electrophysiology,^62^ we removed ECG several features, as follows:

- When it comes to time-domain measures, the RMSSD is highly correlated with the pNN50 and the NN50, and the RMSSD is preferred. Therefore, we removed pNN50 and NN50.
- TINN, HRV Tri Index, VLF and log measurements were removed as they seem to be more indicated for longer periods, and the ECG measurement is 30 s long.

Finally, Empatica E4 data was also processed into HRV data using the Kubios software, as shown in Table B2. To capture physiological states during the time of data collection, we used 10-minute intervals close to the data collection point. Ideally, the intervals started 5 minutes before the time of the stress self-report and continued for 5 minutes after, but due to the amount of noise in the data as well as missing data due to connectivity issues, this was not always possible. Therefore, data was processed as close to the time of data collection as possible. In case there were not 10 full minutes of quality data close to data collection time, we used a cut-off of 5 minutes, that is at least 5 minutes of data were required to be included in the study and processed in Kubios. We removed the same features as above with the exception of very low-frequency and log components which may capture relevant information for longer measurements.

Figure A13 shows the Apple Watch HRV-1 feature in the dataset; Figure A14 shows ECG HRV features; and Figure A15 shows Empatica HRV features.

### Weight, blood pressure, and temperature

Data on weight, blood pressure and temperature were included in this study. For blood pressure, in addition to systolic and diastolic pressure, the mean arterial pressure was used as a feature. While true MAP can only be obtained with intrusive devices, it is possible to estimate it with the following formula: (sys + 3* dys)/3.^49,63^

Due to the size and weight of the Withings Body + smart scale, participants found it difficult to bring this device with them during their daily routine. Therefore, they were instructed to only take weight measurements when they were at home and had access to the scale (which also reflects how such measurements would be taken in the real-world). For this reason, we included weight data in this study despite large gaps, using the KNNImputter to fill these. Figure A16 shows snapshots of weight, blood pressure and temperature features.

#### Sleep

As mentioned in the HR subsection, we calculated mean, maximum and minimum HR during the night (between the last data point collected from the previous day and the first from the current day).

In addition, we calculated sleep features from the Apple Watch and the Withings Sleep device. From both devices, we calculated the following sleep features: Total Time Asleep, Number of Wake-Ups, Time During Awake, Total Time in Bed, and Percentage of Time Asleep While in Bed. Withings Sleep also provided additional information on the time the participant spent in Light, Deep and REM stages, respectively (of note, the Apple Watch recently introduced an update on sleep monitoring that also collects data on sleep stages, but that was not available at the time of the study).^64^

To calculate Apple Watch sleep durations, we made use of both the Apple Watch and iPhone. The Apple Watch calculates times spent asleep. To calculate time in bed, Apple systems make use of the iPhone's sleep calendar feature. Users were asked to include an estimate of their sleep schedules on the iPhone (see User Manual). Those values are updated based on when participants are using the phone and were used to estimate when the user went to sleep.

Because the relationship between sleep and mental health is potentially bidirectional,^65,66^ we also created features offsetting the day for 2 additional days and 2 days before. For example, if a feature was collected at time *t,* the *t* - *2* equivalent would place this value 2 days before, *t* - 1 at 1 day before, *t *+ *1* at 1 day after, and *t + 2* at 2 days after. In order to not greatly increase our feature set, initial RF models were used to calculate the feature importance (based on mean decrease in impurity) of different offset days, with 2 days before and 2 days after being extremely prevalent among the most important features. Therefore, the majority of sleep features included were from *t* - 2*, t* + 2, and *t.* The features from other day offsets that were included, based on the tests, were *t* + 1 Apple Watch Mean HR, *t* + 1 Apple Watch Max HR, *t + 1* Apple Watch Min HR, *t + 1 Withings Total Time Asleep, t + 1* Apple Watch Number of Wake-Ups*, t + 1* Apple Watch Time During Awake*, t* - *1* Apple Watch Mean HR, *t* - *1* Apple Watch Max HR, *t* - *1* Apple Watch Min HR, *t* - *1 Withings Total Time Asleep, t* - *1 Apple Watch Total Time In Bed.*

Finally, because sleep data is collected at a different frequency than each of the EMA data collection – EMA is collected approximately 6 times a day while sleep data is collected once per day – sleep data were included for the entire day (e.g. every measurement of day *t* will have the same sleep features) to maximise the amount of granular stress information collected. Figure A17 shows sleep features for *t.*

### Feature selection and normalisation

In addition to the removal of features mentioned in the previous section, before every experiment, highly correlated features (with a Pearson correlation coefficient higher than 0.95) were removed. While RF is not affected by the difference in units, we normalised the data for input in the SVM models using *SciKit Learn's* Standard Scaler method.^67^

### Analyses/experiments

A number of experiments with different subsets of the data were conducted to allow us to better understand the predictive power of each device/manufactures (Empatica, Apple, Withings) for stress prediction. Further, we conducted the analyses with and without the sleep data due to its different data collection periods. We conducted the following experiments excluding sleep:

- Dataset with all features, D (*n* = 22)
- Dataset with only ECG features, DECG (*n* = 42)
- Dataset with only Apple features, DA (*n* = 42)
- Dataset with only Withings Features, DW (*n* = 44)
- Dataset with Apple and Withings Features, DAW (*n* = 41)
- Dataset with Only Empatica Features, DEmpatica (*n* = 27).

Adding sleep features to the datasets, we conducted the following experiments:

- Sleep Dataset with only Apple features, SDA (*n* = 34)
- Sleep Dataset with only Withings Features, SDW (*n* = 34)
- Sleep Dataset with Withings and Apple Features, SDAW (*n* = 27)
- Sleep Dataset with Withings and Apple Only Sleep Features, SDS (*n* = 27)

DECG and DA contain the same participants, with different features. In case a participant possessed a feature with less than 30% of the data, this participant was removed from datasets using the feature. For example, if a user possesses less than 30% of the Empatica features, they were not included in the D or DEmpatica sets, and similarly for other features in each dataset. For the datasets including sleep features, we did not include Empatica data as this would result in very small datasets. Table B3 shows the participant characteristics for each dataset, and each separate sampling is further discussed in the ‘Results’ section.

As mentioned in the ‘Related work’ section, there are many different ways to train, test and validate the models in the literature. For these analyses, given that in real-world deployment public health agencies would collect a large amount of data from populations, we elected to train generalised models, meaning using data from all participants. Randomly, 80% of the dataset is used for training/validation and hyper-parameter tuning with 10-fold cross-validation, while 20% is used for testing.

In addition, since data was collected in real-life environments, many users had a predominance of one class over another (e.g. with a lot of data points classified as *no stress* compared to *stress*, or vice-versa). For this reason, we conducted the analyses with imbalanced classes as well as with balanced classes using the SMOTE (Synthetic Minority Over-sampling Technique) method on *SciKit Learn,* which upsamples the minority class.^21,68^ The technique is applied only to the training sets in the examples described above before cross-validation to make sure the model is tested on real data.^21^ In other words, only the train sets are balanced.

Finally, due to the relationship between stress measures and factors such as sex, age, income, work, and health, we trained the following models^20^:

1. Total: comprised of data from all participants in each subset.
2. Age: models in the age range of 18–24, 25–34 and 35–44 were trained. We decided not to train models in the 45–64 range due to the scarcity of participants in this interval. By the same token, we did not train a model for participants aged above 65 as only one participant was in that category.
3. Sex: we trained models for male and female participants. We did not train a model for the participant that self-identified as gender fluid as only one participant was in that category.
4. Income: we trained models for participants belonging to low socioeconomic status (SES), comprising participants that earn less than CAD 30,000, and participants belonging to middle and high SES. The CAD 30,000 cut-off point was based on an approximation of the Canadian tax cut-off for low-income populations.^69^
5. Profession: we trained models for workers (full-time, part-time, and participants that are self-employed or classified as other) and students. We did not train a model for the retired participant as only one participant was in that category.
6. Healthy: we trained a model removing participants that reported chronic diseases, illnesses, frequent alcohol or drug use, or prescription drug use.

**Figure 3.**
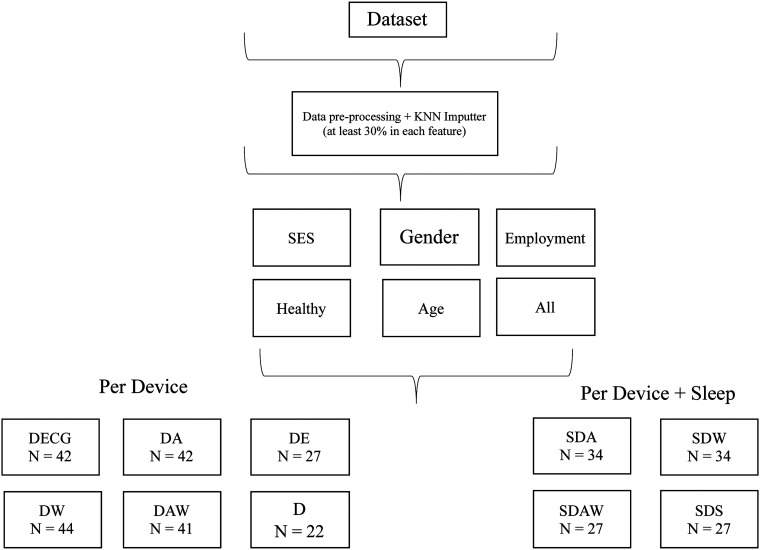
Division into datasets per device and per device + sleep.

**Figure 4.**
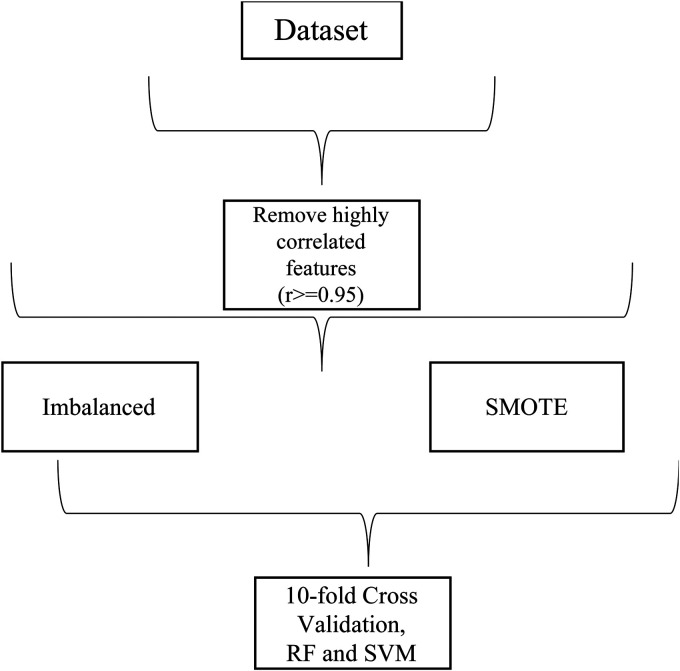
Training generalised models.

For each of these divisions, we trained the model with binary classification (*stress* vs *no stress*), reporting accuracy, f1-weighted and f1-macro score in tables B4 and B5 from Appendix 2. Finally, while the SVM model performs many transformations to fit the data, making it harder to obtain information on the importance of features, we calculated feature importance for the RF model using the mean decrease in impurity.^20^ A 100% purity in a node means the decision tree's node contains only one class, and by assessing the change in impurity between parent and child nodes we can calculate the best split in the tree and use it as a proxy for feature importance. Figure 3 and Figure 4 show the process of obtaining the different datasets and training the models.

## Results

### Population data characteristics

Table 2 details the full sample (*n* = 45) used in the study. Most participants are aged 44 years or younger (87%), female (67%), with low (44%) or medium (40%) income and workers (62%). In terms of ethnicity, a majority identified as white (33%), South Asian (24%) or Latin American (22%). Most participants (80%) did not have chronic diseases or illnesses, use prescription drugs or frequently consumed alcohol/smoke. The average of days a participant had in the study was 17.1 (±2.5), and participants had an average of 78.91 (±11.0) data points. Data quality did not visibly differ between participants based on number of days required for data collection.

**Table 2.** Participant characteristics.

Participants (*N* = 45)	Frequency	Percentage
*Age*		
18–24	13	29
25–34	14	31
35–44	12	27
45–64	5	11
Above 65	1	2
*Sex/Gender*		
Male	14	31
Female	30	67
Gender Fluid	1	2
*SES*		
Low (0-$30,000)	20	44
Medium ($30,000– $100,000)	18	40
High (Above $100,000)	4	9
Do not wish to disclose	3	7
*Profession*		
Full-time	21	47
Part-time	5	11
Student	16	36
Self-employed/Other	2	4
Retired	1	2
*Ethnicity*		
Black and Southeast Asian	1	2
Black or African American	3	7
Chinese	4	9
Indian	1	2
Latin American	10	22
South Asian	11	24
White	15	33
*Health Status*		
Healthy	36	80
Chronic Disease or Illness, Prescription Drug Use, Smoking or Alcohol	9	20

For the 45 participants, 43% of total answers were classified as stress (1539), while the remaining 57% (2012) were labelled as no stress. This proportion is maintained, approximately, for each of the subsamples used in the analyses (Table B3 in Appendix 2).

## Models

This section discusses the results of each of our models. The focus of this section is on the f1-macro metric, as accuracy may not reflect imbalance in classes (if one class is predicted well but another is not, the accuracy may still be high), so the f1-score which calculates the harmonic mean between precision and recall is preferred. Further, the macro average treats both classes as being of equal importance.

Table 3 shows the f1-score for the experiments. More detail on other metrics, including specifics for each class, accuracy, f1-weighted, precision and recall can be found in Table B4 (without sleep) and B5 (with sleep). Feature importance in each dataset is presented in Tables B7 to B16 in Appendix 2 for each dataset.

**Table 3.** Results for generalised models.

Dataset with all features (D)				
	**RF**	**SVM**	**RF-SMOTE**	**SVM-SMOTE**
All	0.66	0.65	0.68	0.65
Gender – Male	0.68	0.7	0.63	0.64
Gender – Female	0.68	0.61	0.66	0.65
Employment – Student	0.61	0.62	0.6	0.62
Employment – Worker	0.62	0.61	0.63	0.58
Income – Low	0.62	0.63	0.63	0.59
Income – Medium High	0.73	0.7	0.71	0.7
Age – 18–24	0.51	0.39	0.52	0.47
Age – 25–34	0.56	0.62	0.6	0.44
Age – 35–44	0.5	0.55	0.54	0.62
Healthy	0.68	0.68	0.67	0.62
**Dataset with only ECG features (DECG)**				
All	0.62	0.53	0.61	0.54
Gender – Male	0.61	0.6	0.62	0.59
Gender – Female	0.62	0.59	0.62	0.56
Employment – Student	0.64	0.6	0.62	0.6
Employment – Worker	0.6	0.56	0.59	0.47
Income – Low	0.53	0.55	0.53	0.46
Income – Medium High	0.67	0.61	0.67	0.58
Age – 18–24	0.61	0.58	0.57	0.51
Age – 25–34	0.59	0.59	0.59	0.51
Age – 35–44	0.58	0.56	0.61	0.49
Healthy	0.58	0.55	0.62	0.57
**Dataset with only Apple Features (DA)**				
All	0.63	0.58	0.63	0.53
Gender – Male	0.63	0.61	0.64	0.58
Gender – Female	0.59	0.53	0.59	0.58
Employment – Student	0.65	0.54	0.63	0.58
Employment – Worker	0.63	0.57	0.61	0.54
Income – Low	0.54	0.52	0.58	0.55
Income – Medium High	0.67	0.6	0.66	0.59
Age – 18–24	0.54	0.56	0.53	0.55
Age – 25–34	0.6	0.57	0.63	0.56
Age – 35–44	0.59	0.61	0.65	0.52
Healthy	0.6	0.56	0.62	0.57
**Dataset with Apple and Withings Features (DAW)**				
All	0.67	0.6	0.69	0.61
Gender – Male	0.73	0.7	0.71	0.7
Gender – Female	0.67	0.63	0.66	0.58
Employment – Student	0.65	0.64	0.62	0.61
Employment – Worker	0.66	0.63	0.67	0.64
Income – Low	0.53	0.58	0.59	0.56
Income – Medium High	0.72	0.65	0.71	0.67
Age – 18–24	0.6	0.59	0.63	0.6
Age – 25–34	0.69	0.63	0.66	0.58
Age – 35–44	0.61	0.69	0.67	0.57
Healthy	0.62	0.63	0.6	0.6
**Dataset with only Withings Features (DW)**				
All	0.69	0.62	0.66	0.64
Gender – Male	0.65	0.68	0.63	0.63
Gender – Female	0.63	0.57	0.64	0.62
Employment – Student	0.65	0.66	0.64	0.6
Employment – Worker	0.66	0.64	0.66	0.63
Income – Low	0.58	0.62	0.55	0.57
Income – Medium High	0.73	0.65	0.69	0.69
Age – 18–24	0.51	0.55	0.53	0.55
Age – 25–34	0.6	0.57	0.63	0.56
Age – 35–44	0.62	0.64	0.65	0.56
Healthy	0.62	0.58	0.61	0.58
**Dataset with only Empatica Features**				
All	0.64	0.6	0.65	0.61
Gender – Male	0.64	0.7	0.67	0.59
Gender – Female	0.68	0.66	0.67	0.66
Employment – Student	0.67	0.66	0.68	0.63
Employment – Worker	0.65	0.67	0.65	0.65
Income – Low	0.56	0.57	0.57	0.56
Income – Medium High	0.65	0.66	0.67	0.66
Age – 18–24	0.48	0.53	0.58	0.47
Age – 25–34	0.68	0.67	0.67	0.62
Age – 35–44	0.54	0.68	0.62	0.61
Healthy	0.61	0.61	0.63	0.6
**Sleep Dataset with only Apple Features (SDA)**				
All	0.7	0.65	0.73	0.66
Gender – Male	0.63	0.63	0.65	0.64
Gender – Female	0.71	0.66	0.71	0.64
Employment – Student	0.69	0.67	0.69	0.67
Employment – Worker	0.66	0.65	0.68	0.61
Income – Low	0.7	0.61	0.71	0.66
Income – Medium High	0.75	0.72	0.72	0.67
Age – 18–24	0.7	0.65	0.71	0.64
Age – 25–34	0.68	0.61	0.69	0.64
Age – 35–44	0.68	0.67	0.68	0.61
Healthy	0.72	0.67	0.71	0.64
**Sleep Dataset with Apple and Withings Features (SDAW)**				
All	0.73	0.69	0.72	0.71
Gender – Male	0.53	0.53	0.53	0.45
Gender – Female	0.7	0.67	0.71	0.66
Employment – Student	0.74	0.7	0.74	0.74
Employment – Worker	0.67	0.65	0.7	0.63
Income – Low	0.74	0.7	0.74	0.71
Income – Medium High	0.67	0.69	0.67	0.68
Age – 18–24	0.75	0.74	0.73	0.75
Age – 25–34	0.71	0.65	0.69	0.68
Age – 35–44	0.63	0.68	0.65	0.53
Healthy	0.69	0.67	0.7	0.7
**Sleep Dataset with Withings Features (SDW)**				
All	0.69	0.58	0.67	0.68
Gender – Male	0.79	0.81	0.8	0.8
Gender – Female	0.68	0.66	0.67	0.65
Employment – Student	0.71	0.73	0.73	0.73
Employment – Worker	0.67	0.65	0.68	0.64
Income – Low	0.67	0.68	0.69	0.68
Income – Medium High	0.71	0.73	0.71	0.73
Age – 18–24	0.67	0.72	0.65	0.67
Age – 25–34	0.73	0.75	0.7	0.74
Age – 35–44	0.72	0.73	0.73	0.68
Healthy	0.72	0.7	0.7	0.72
**Sleep Dataset with Withings and Apple Only Sleep Features (SDS)**				
All	0.7	0.69	0.7	0.71
Gender – Male	0.45	0.45	0.47	0.49
Gender – Female	0.71	0.71	0.71	0.71
Employment – Student	0.73	0.74	0.71	0.74
Employment – Worker	0.66	0.65	0.63	0.62
Income – Low	0.69	0.71	0.73	0.73
Income – Medium High	0.66	0.66	0.63	0.65
Age – 18–24	0.65	0.6	0.65	0.61
Age – 25–34	0.67	0.67	0.68	0.69
Age – 35–44	0.6	0.6	0.64	0.53
Healthy	0.73	0.73	0.73	0.74

### Generalised

#### Without sleep data

In this section, we discuss the results of the models for each of the datasets. As can be seen by Table 3, which shows the results for each dataset, most contain results above 60% when considering all participants in the specific samples. In particular, D, DAW and DW results for all participants are above 65% for RF, indicating that Withings features seem to be good predictors and that RF generally works better than SVMs, which generally has lower results. Datasets containing only Apple features, and in particular only ECG, perform worse compared to others, although several results are still above 60%.

Looking only at the healthy participants when compared to all, in most cases the f1-macro score varies slightly (e.g. dropping from 63% to 60% for RF in DA or improving from 66% to 68% for RF in D). In general, stratifying participants by gender and employment improves results, although that is also not always the case – especially with female participants. When stratifying according to income, the divisions with low-income participants typically perform worse, while divisions containing participants with medium to high-income show improvement, often with an f1-macro average above 70%. Finally, stratifying by age seems to worsen results in most cases.

SMOTE results show mild improvement over results without it in most cases, especially for RF. However, many examples indicate worsening results – especially for SVM – or do not demonstrate any improvement.

In terms of feature importance for the RF model, Tables B7 to B12 in Appendix 2 show the top 10 most important features and importance value – calculated as the mean decrease in impurity – for each dataset and stratification. Looking into each of these, we investigated the top 10 features that repeat across strata. For example, for gender, the only top 10 feature in the D dataset that appears in both male and female stratification is weight, as can be seen in Figure A1 in Appendix 1 with a frequency of 2 (meaning the feature appeared in the 2 gender-related models). We did the same for income (low/medium and high), employment (workers and students), age (18–24, 25–34 and 35–44) and finally, between datasets with all participants and datasets with only healthy participants. Figures A1 to A9 show this frequency for the stratifications in each dataset. DW was not included in these analyses as it only has 5 features.

When considering the importance of all features in D, a mix of features from different sources can be seen, including Apple data from the ECG (ECG_DC, ECG_AR_AbsolutePower_HF, ECG_AR_AbsolutePower_LF, ECG_Stress Index), Apple data from HRV (HRV-1) Withings blood pressure data (MAP, dia), and Empatica data (Empatica_AR_RelativePower_LF, Empatica_AR_LFHF). This mix of modalities is maintained throughout other stratifications in D, although with different features for different strata (such as sys and weight in male stratification). Interestingly, ECG_DC is present in several analyses and repeated in stratifications such as all/healthy, income, employment and age. This feature is also prominent in other datasets that it is present.

Withings features are very prominent in most datasets that mix features from different devices, ranking high among the most important features (e.g. MAP or weight are ranked among the top 2 most important features in several stratifications in D and DAW), and Withings features repeat among stratifications in these datasets. Also, of note, the User feature appears frequently in DECG, DA, DAW, and DEmpatica.

#### With sleep data

In general, adding sleep data to the dataset improves results, especially with the RF algorithm, with most results above 65% and an f1-macro score of 70% being commonplace. There were few cases in which the metrics worsened – the latter is mainly seen in SDAW on stratifications such as gender and income. In particular, male participants from SDAW showed a great decrease from f1-macro scores around 70% to results in the low fifties. This might be due to a decrease in the number of male participants from DAW to SDAW (29% to 22%, respectively), which may not have given the model enough data to be trained accurately. For several sleep datasets, upsampling classes using SMOTE resulted in slight improvements, although a worsening result can be seen in some cases. Since sleep data is repeated over a day, the SMOTE method possibly did not accurately synthesise minority class samples.

Finally, while the dataset using only sleep features, SDS, typically showed results on par with the other models, it also produced the worst results among the sleep datasets, notably on male participants (f1-macro score below 50%) and on participants aged 35 to 44 (53% f1-macro score with SVM-SMOTE), suggesting these features are more robust when used in conjunction with others.

When looking at feature importance, sleep features typically dominate the datasets in Tables B13 to B16. Interestingly, in datasets that mix Apple and Withings features (SDAW, SDS), there is still a predominance of Apple Watch sleep features, although several Withings non-related features (e.g. MAP, Weight, sys, temp) are prevalent among the top 10 most important features in each stratification. When looking at SDW, containing only Withings features, there is also a prevalence of non-sleep related features, and these features are generally repeated among stratifications.

The Apple Watch Consolidated Time During Awake feature, and its offsets for *t* + 1 and *t* + 2, also repeat among stratifications in SDA, SDS and SDAW.

## Discussion

### Stress models

While the use of different metrics for model evaluation in literature, as well as the different strategies for data collection and model training, difficult comparisons between works, the results of the Generalised models achieved good to great accuracy between 60% and 70% (Tables B4 and B5), which is in line with the state-of-the-art – particularly for studies that use real-life data (see Table B6 in Appendix 2 with studies labelled DDSR, meaning they were trained with daily life self-report labels). The promising f1-macro score values shown in Table 3 indicate the models are able to predict the two classes. This indicates that the MHP provided accurate and representative data and that a similar system could be deployed by public health agencies for data collection and monitoring of a condition in a population, such as stress. This is especially promising considering the models were built on data collected from personal, consumer-level, off-the-shelf devices rather than using data from research-grade equipment.

In terms of which features to collect, Apple Watch sleep features are very prominent among the sleep datasets, usually ranking higher than other features. Other features such as temperature, weight and blood pressure also appear as good predictors when looking at feature importance (all Withings related). Datasets that integrated sleep features achieved the best results, with an f1-macro score and accuracy typically above 70%. In addition, feature importance results suggest that offsets of the data should be considered. Specifically, *t* + 2 and *t* - 2 demonstrated good results, although offsetting for a day also provided important features in specific cases. Since this study, Apple has updated its sleep data collection to include additional features such as sleep stages,^70^ which could improve performance even more – especially as time Spent in REM was one of the few Withings sleep features that appeared repeatedly among stratifications.

In terms of non-sleep data, datasets that contained Withings data (D, DW, DAW) typically performed better than others. Coupled with the prevalence of Withings non-sleep features among the important features in the sleep datasets, this suggests that using these devices to collect temperature, weight and blood pressure would be an interesting avenue of research to follow.

Encouragingly, Empatica E4 data did not seem to greatly affect the models, as they usually performed well without this data, specially sleep-related models. Given this, public health agencies could potentially leverage data from personal, consumer-level devices, rather than having to resort to medical-grade wearables such as the Empatica E4.

When looking into Apple Watch ECG data alone, the metrics worsen compared to other datasets, although they are generally above 60%. This is an improvement over previous work by the authors using the ECG dataset without any missing data imputation and with only a subset of the data.^20^ Much like in the previous work, ECG_DC and, to a lesser extent, ECG_AC, featured prominently in ECG-related models. In the present case, ECG_DC also features in datasets containing other data modalities in addition to ECG. Empatica_DC was also a prominent feature when looking at the Empatica data and among some stratifications. Therefore, the heart's deceleration (DC) and even the heart's acceleration (AC) seem to be valuable HRV metrics for the models, being constantly present among the top 10 most important features. AC and DC are relatively new indicators in HRV studies, and it would be interesting to conduct further research into stress using AC and DC to establish if they can be robustly used to differentiate stress states.

When considering the f1-scores for each class in Tables B4 and B5, generally, the ‘no stress’ class seems to outperform the ‘stress’ class, especially for the non-sleep datasets (Table B4). This suggests that the models typically have higher specificity than sensitivity.

### Performances for different samples in datasets

Given the different samples use for each dataset, it is worthwhile to look at how the datasets differ regarding these samples and how this may affect results (Table B3 in Appendix 2). D contains fewer participants in the 18–24, 25–34 and 35–44 ranges, which may account for this dataset generally having poor results in these stratifications when compared to others. However, D has good results on the male/female and low/medium and high-income stratifications despite possessing half the number of men, women and participants in low to middle income (with high-income participants removed entirely). Therefore, it is likely that the division of fewer participants into three distinct age categories did not provide enough information to train the model, and future research collapsing the age category into fewer divisions (e.g. young vs. old) as opposed to the intervals presented here could lead to better results.

D also has lower results than other datasets for profession, likely due to the removal of several workers from the dataset. Interestingly, while D has a 7% increase in the proportion of unhealthy participants, it also has the highest non-sleep related metric for the healthy stratification. With a decrease from 36 healthy participants in the full sample to 16 in D, a higher homogenisation of participants likely led to better metrics. In particular, of the healthy participants in D, 63% were female, 50% were students and 56% were low income. This suggests that creating models for more than one stratification (e.g. healthy women) may lead to better results. On the other hand, it will reduce the dataset even further, which may affect the model's effectiveness. Future work, with more purposeful sampling could lead to further insights into this avenue of research.

DEmpatica is another dataset that had a large number of participants excluded, and similar observations to D can be made: results in the age stratifications were poor, with other stratifications performing better. Stratifying by profession fared better in this dataset, with a larger number of workers and students. The number of healthy participants also increased, leading to lower metrics – likely due to more heterogeneous participants. When looking at demographics, DEmpatica has a higher proportion of healthy females (65%), a slight decrease in healthy low-income participants (55%), and a higher decrease in students (40%), suggesting that future sampling of healthy participants by profession could lead to additional insights.

DECG/DA contain the same sample of participants, with very similar proportions to the entire sample. These datasets did not generally perform as well as other models containing Withings features, likely due to lower predictive power in non-sleep related Apple features. Indeed, DAW – with 4 fewer participants than the total sample of 45 while still maintaining similar proportions – performed better, reflecting the higher feature importance of Withings features. DW has very similar proportions to DAW with only 1 participant removed from the total sample and, when comparing this dataset to DAW, we can see that using only Withings features led to good results, sometimes better than using Apple and Withings features combined.

In general, regardless of how the samples varied – and indeed the sleep datasets had more users removed due to missing data – sleep datasets performed better than datasets without sleep features, highlighting their importance. SDA had major changes regarding income (increase from 44% to 50% in low-income and 9% to 0% in high-income participants). Interestingly, these reductions led to slightly better results in general when compared to other datasets, likely due to increased homogenisation of participants. Most low-income participants in this dataset were female (71%) students (77%) aged 18 to 24 (53%) while most medium-income participants were female (64%) workers (94%), aged 25 to 34 (50%).

Despite having fewer participants, SDAW maintained good results, indicating the importance of both sleep-related features and non-sleep related Withings features. This is reflected also in SDS, which uses the same sample but with worsening results due to the removal of non-sleep Withings features. Both SDAW and SDS had a 9% decrease in male participants; given the already low prevalence of men, this may explain the poor results for these stratifications. Finally, as we saw in the previous subsection, Withings features are particularly important for SDW.

### Limitations, lessons learned and implications for public health

In this section, we describe empirical lessons derived from deploying the MHP prototype in real-life and developing the ML models and discuss implications for the use of a similar system in public health. In the process, we also discuss study limitations as well as mitigation strategies where applicable.

First, a limitation of this study is that, due to convenience sampling, most participants were female, typically white and young. Looking at sex/gender in particular, most datasets contain approximately 30% men and 70% women, and their model results metrics are similar. As mentioned, on SDW and SDS, the male stratification performs poorly; in these datasets, the proportion of male participants is closer to 20%, which could indicate there is a lack of data from these participants to accurately train the models. The same may have happened for other stratifications, such as income and age, which performed poorly in several cases. As mentioned, future studies with more purposeful sampling could lead to better stratifications and further insights into how models will perform for different traits.

The RF model in general performed better than the SVM model. The SMOTE method was used in this work with mixed results to handle class imbalances, and careful consideration must be taken to generate synthetic data for public health decision-making. However, if a system similar to the MHP is deployed in the real-world, it would potentially capture much larger datasets from each user – for example, by collecting data points for longer periods – generating datasets with more examples in each class that would balance the collected dataset, thus mitigating this issue. Since models in general have a higher specificity than sensitivity, real-world deployment should take into account the fact that the prediction models may be better at identifying when individuals are not stresses as opposed to stressed. To mitigate this, it would be interesting to investigate the use of reinforcement learning, in which the model learns from mistakes through a reward and punishment approach.^71^ For example, the mobile application could display the prediction result to the user and ask if this prediction is correct. This feedback will provide rewards or punishment to the model, improving future predictions.

In terms of feature importance, Apple Watch sleep features as well as Withings non-sleep related features (such as temperature, weight and blood pressure) were shown to be important for models and should be prioritised. On that note, a limitation of the data collection method was the amount of missing data. 10 participants had over 70% missing Apple Watch sleep data, and an additional 10 had missing Withings sleep data. While the missing Apple Watch sleep data was likely due to participants not wearing the device while sleeping, the Withings Sleep device is placed below the mattress, and so should be available at all times when connected to a plug. Discussing the device with participants during the video call and looking at the data, it seems this error is more likely due to limitations with the device.

Indeed, several challenges occurred with the Withings Sleep. First, the device needs to connect to a Wi-Fi network. One participant could not use the device as their network was part of a university, and a certificate was needed to connect. Since the certificate could not be downloaded onto Withings Sleep, the device did not work for this individual. In addition, the integration between Withings Sleep and Apple Health did not work consistently. The MHP collects data from the Apple Health app, and the Withings Sleep device is synced to the Withings proprietary Health Mate app (see User Manual for more details on this app). Health Mate, then, integrates with Apple Health after the data-sharing option is selected in the app. Despite this, in many situations Health Mate did not share sleep data with Apple Health, requiring the data sharing option on Health Mate to be turned on and off by the researcher or participants. There were also many situations where researchers did not observe sleep data being synced from the Withings Sleep device and, after asking participants to reset the iPhone, data collection proceeded as normal. One Withings Sleep device also broke during the study. Finally, although the Withings Sleep device was reset to factory mode before being provided to a participant, there were many cases during the video call where participants were required to reset it to this mode again. Given these issues, and that Withings Sleep data was not shown to be among the most important predictive features, Apple Watch sleep data can be prioritised in further deployments.

The Empatica E4 device also demonstrated issues during data collection. To collect real-life data, the device had to be used in Bluetooth mode, which required a connection with the E4 Realtime app on the iPhone (see User Manual in Appendix 3). However, if the device was out of range, no alert was given when the device disconnected and the data collection stream interrupted. To mitigate this issue, participants were asked to monitor the E4 Realtime app and reconnect the device as needed. In addition, we also experienced technical issues with the Empatica devices, as during the 1-year data collection period, 3 out of 4 available devices broke and had to be fixed before collection could proceed. Movement also introduced a lot of noise in the data, making a lot of it unusable. For this reason, half of participants (28 out of 45) did not have at least 30% of the necessary Empatica data to use in the analyses. As discussed, given that Empatica features were not among the most important features, this device does not need to be included in future work to complement data from personal devices.

The prototype version of the MHP did not have any backup features in case data sharing between the device and the research database did not work (e.g. due to low connectivity). To mitigate this issue, manually exported data from Apple Health was compared to the MHP data to make sure missing information due to issues in data sharing is considered in the analysis. Future versions of the MHP should contain a failsafe in case the connection does not work. In particular, the Information Property List Files (*info.plist*) in the device might be an interesting solution: *Info.plist* is a structured text file, available for edits in Apple's iOS development software XCode,^72^ that contains information describing the app's configuration, and data can be stored in it using a dictionary format.^73^ Storing information that could not be shared with the database on this file, retrieving it and sending it again would be a potential backup solution that would not require any online storage.

The prototype MHP contained data types that were hardcoded into the source code. In case researchers need to collect additional data types, the code for requesting additional authorisations in Apple Health and for additional HealthKit queries needs to be developed. One interesting update to future versions would be to allow researchers to customise which data they want to collect. By hardcoding queries for Apple Health data types and allowing users to activate them through the app's interface, the MHP would be flexible in enabling public health scientists to collect different types of data depending on their need. On the same token, the devices used in the study were hardcoded into the app prior to data collection, but a real-world deployment could enrol new devices belonging to each user by obtaining device information from the data sources in Apple Health obtained through HealthKit.

Of note, the MHP queries also enabled data collection in the background, that is, while the app is not terminated but also not being currently used. However, if the app is not regularly used, the background queries will not be triggered constantly and may be terminated by the iPhone's operating system. Our solution to this was creating new queries every time the app is opened and terminating the app every time it is placed in the background to make sure the queries are triggered. Ideally, users would constantly utilise the system, triggering background data collection. While out of the scope of this work, to encourage people to use the MHP, there are many possible strategies, such as gamification^74^ or developing an interface that allows users to monitor and manage their health in addition to collecting the data.^75^ Finally, public health agencies can use such a system not only for monitoring but also for intervention. For example, if a user's stress levels are high, the MHP could trigger a meditation app (potentially the Breathe app on the Apple Watch for Apple systems). This feedback and intervention loop can reach a lot of people in real-time and fulfill the mission of public health – improving the quality of life of populations.

In terms of study design, the data collection protocols required several devices and apps to be used during participants’ daily life, which can be quite intrusive and demanding. In particular, many had difficulties leaving the Breathe app open for 5 minutes to collect HRV data without making any movements, which would stop data collection. For this reason, we decided not to use the 5-minute HRV data, instead using HRV collected randomly throughout the day (HRV-1). Interestingly, HRV-1 is an effective feature, appearing among the top 10 most important features in many stratifications in datasets containing Apple data.

In the case of real-world deployment, however, it cannot be expected that users will possess all devices described in the study or that they will collect data constantly. To mitigate this issue, as we discussed, the Apple Watch was the most important device used in this study, particularly its sleep data, which produced better models. Weight was also shown to be an important feature – even though weight data was only collected when participants were at home – and it is very common for individuals to have scales as a regular household item. Even if they are not wireless devices, manual input of weight into the phone can still be done. The same can be said for thermometers, and to a lesser extent blood pressure cuffs. Therefore, it should not be hard to obtain data for the most important features. Further, to reduce the burden on users, it might be more useful to ask for fewer data points over a longer period (e.g. once in the morning and once at night for several months) rather than for several data collected throughout the day. As discussed, this could help with obtaining datasets with more examples of each prediction class. In this way, public health researchers can obtain large datasets to build models and study individual health while placing less burdens on users.

## Conclusions

In this study, we developed an MHP that collects data from Apple Health – which in turn integrates data from mobile and wearable devices – to be used in public health data collection efforts. To test its efficacy, we predicted the stress states of individuals using ML models of RF and SVM based on Apple Health data gathered throughout participants’ daily routines and collected using a MHP.

Additional future work should implement improvements on the MHP such as backup in case of an error in data sharing, customisation of data collection and encouragement for people to use the platform (e.g. gamification, health management features). In addition, further validation of the models in more controlled environments (such as in a lab, where stressors can be applied and controlled to generate balanced labeled data) would allow more robust evidence of their efficacy. Purposeful sampling will also allow the generation of more robust models for different stratifications.

The development of the MHP and stress prediction models indicates that mobile systems can be successfully used as a data collection tool. RF models perform well, and sleep data from the Apple Watch as well as Withings features, such as weight and temperature, are important predictors. This work suggests that the iOS system and Apple Health can be used to monitor data for public health surveillance, and Apple and Withings devices can be used to study and predict conditions in a population such as stress. The platform presented here represents a step towards a future in which smart technologies can be used in conjunction with self-report data collection methods to enable new insights into the health of populations.

## Supplemental Material

sj-docx-1-dhj-10.1177_20552076241249931 - Supplemental material for Application of a mobile health data platform for public health surveillance: A case study in stress monitoring and prediction Supplemental material, sj-docx-1-dhj-10.1177_20552076241249931 for Application of a mobile health data platform for public health surveillance: A case study in stress monitoring and prediction by Pedro Elkind Velmovitsky, Paulo Alencar, Scott T Leatherdale, Donald Cowan and Plinio Pelegrini Morita in DIGITAL HEALTH

sj-docx-2-dhj-10.1177_20552076241249931 - Supplemental material for Application of a mobile health data platform for public health surveillance: A case study in stress monitoring and prediction Supplemental material, sj-docx-2-dhj-10.1177_20552076241249931 for Application of a mobile health data platform for public health surveillance: A case study in stress monitoring and prediction by Pedro Elkind Velmovitsky, Paulo Alencar, Scott T Leatherdale, Donald Cowan and Plinio Pelegrini Morita in DIGITAL HEALTH

sj-docx-3-dhj-10.1177_20552076241249931 - Supplemental material for Application of a mobile health data platform for public health surveillance: A case study in stress monitoring and prediction Supplemental material, sj-docx-3-dhj-10.1177_20552076241249931 for Application of a mobile health data platform for public health surveillance: A case study in stress monitoring and prediction by Pedro Elkind Velmovitsky, Paulo Alencar, Scott T Leatherdale, Donald Cowan and Plinio Pelegrini Morita in DIGITAL HEALTH

## Abbreviations

AH: Apple Health
API: Application Programming Interface
BP: Blood Pressure
COVID-19: Coronavirus
DASS-21: Depression, Anxiety and Stress Scale
ECG: Electrocardiogram
HR: Heart Rate
HRV: Heart Rate Variability
IoT: Internet of Things
JMIR: Journal of Medical Internet Research
kNN: k-Nearest Neighbours
LOPO: Leave-One-Person-Out
LR: Logistic Regression
ML: Machine Learning
MAP: Mean Arterial Pressure
MHP: mobile health platform
NN50: Number of successive RR interval pairs that differ more than 50 ms
pNN50: NN50 divided by the number of RR intervals
RCT: randomised controlled trial
RF: random forest
RMSSD: Root Mean Square of Standard Deviation of Intervals
SMOTE: Synthetic Minority Over-sampling Technique
SVM: Support Vector Machine
UML: Unified Modeling Language
