# Supplementary material for: Application of a mobile health data platform for public health surveillance: A case study in stress monitoring and prediction
Source: Digit Health. 2024 Jun 8;10:20552076241249931. doi: 10.1177/20552076241249931 (PMC11394344; doi:10.1177/20552076241249931)
Supplement: sj-docx-1-dhj-10.1177_20552076241249931 - Supplemental material for Application of a mobile health data platform for public health surveillance: A case study in stress monitoring and prediction [file sj-docx-1-dhj-10.1177_20552076241249931.docx]

## Supplementary Material

## Appendix A – Additional Figures

Figure A1: Frequency of top 10 features in each stratification, dataset D

Figure A2: Frequency of top 10 features in each stratification, dataset DECG

###

Figure A3: Frequency of top 10 features in each stratification, dataset DA

Figure A4: Frequency of top 10 features in each stratification, dataset DAW

Figure A5: Frequency of top 10 features in each stratification, dataset DEmpatica

Figure A6: Frequency of top 10 features in each stratification, dataset SDA

Figure A7: Frequency of top 10 features in each stratification, dataset SDAW

Figure A8: Frequency of top 10 features in each stratification, dataset SDW

Figure A9: Frequency of top 10 features in each stratification, dataset SDS (no features repeated for Gender)


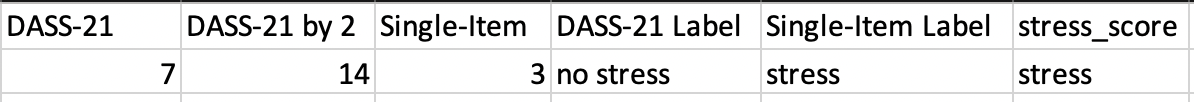
Figure Figure A10: Example of Stress Scoring


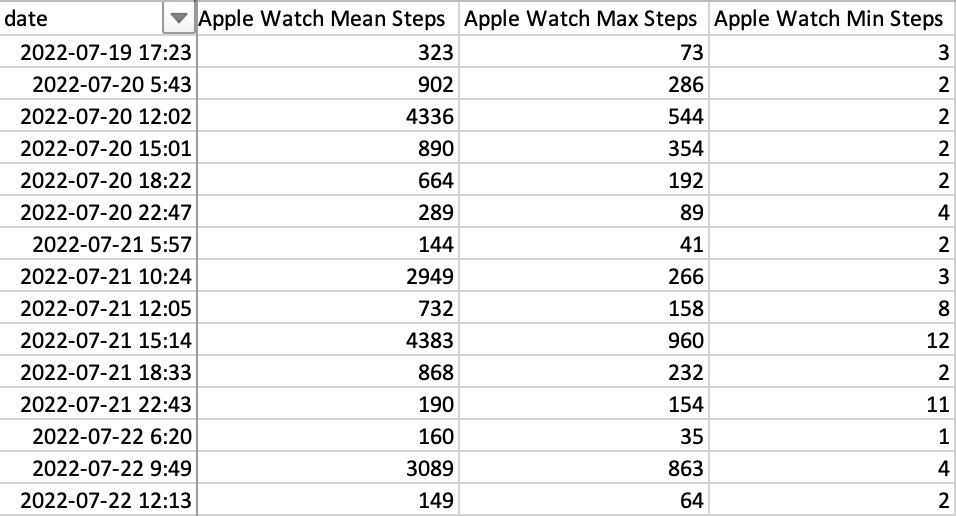


Figure A11: Snapshot of Steps Features


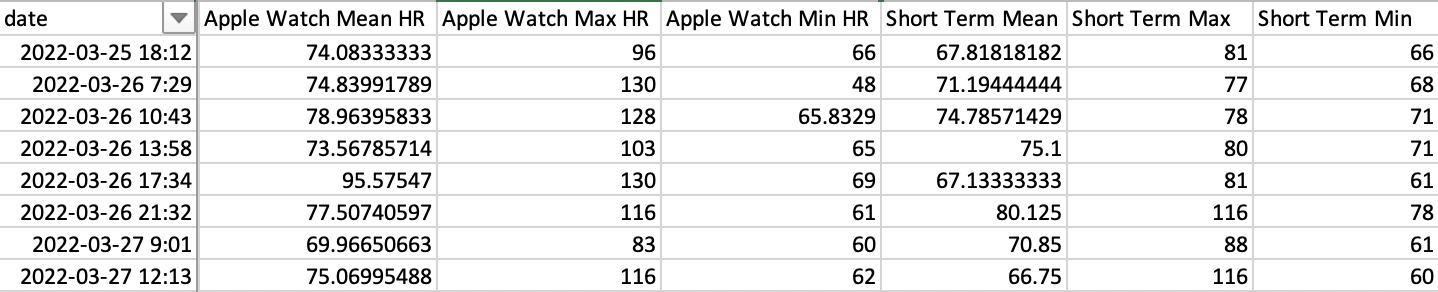


Figure A12: Snapshot of Apple Watch HR features


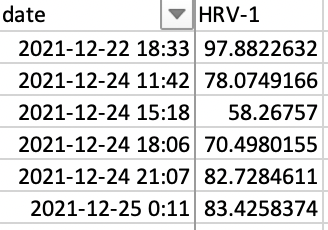


Figure A13: HRV-1 feature in dataset

**
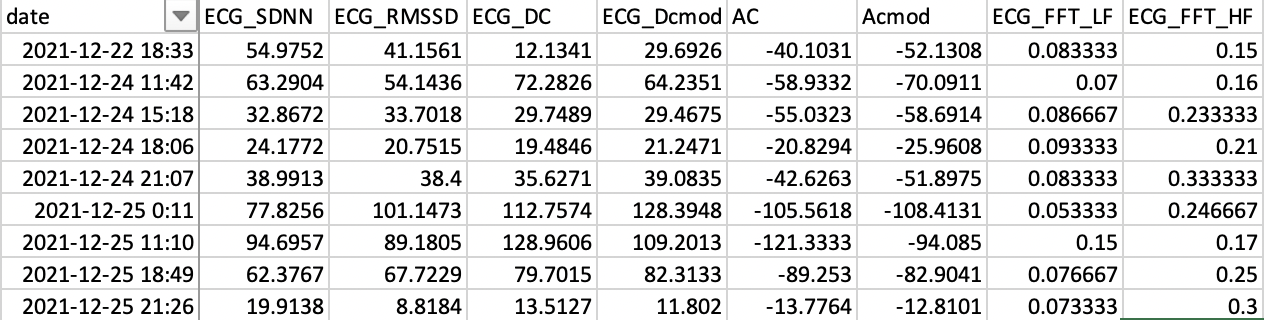
**

Figure A14: Snapshot of ECG HRV features

**
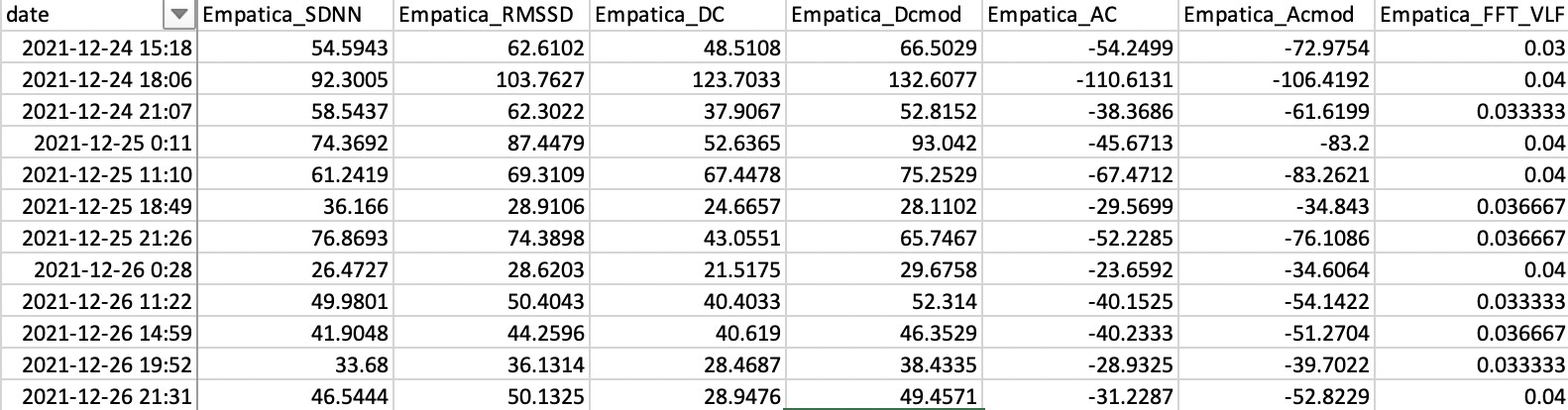
**

Figure A15: Snapshot of Empatica HRV features


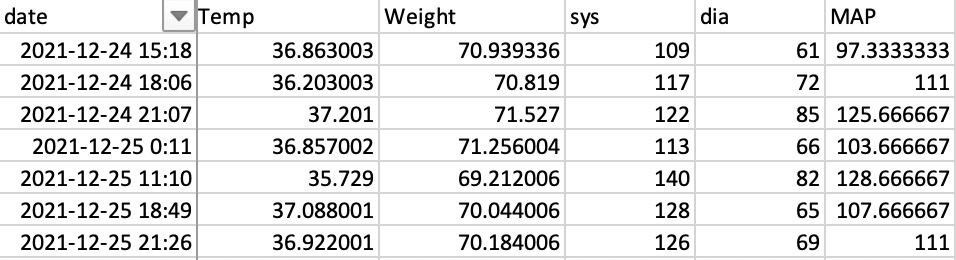


Figure A16: Snapshot of Temperature, Weight and Blood Pressure Features


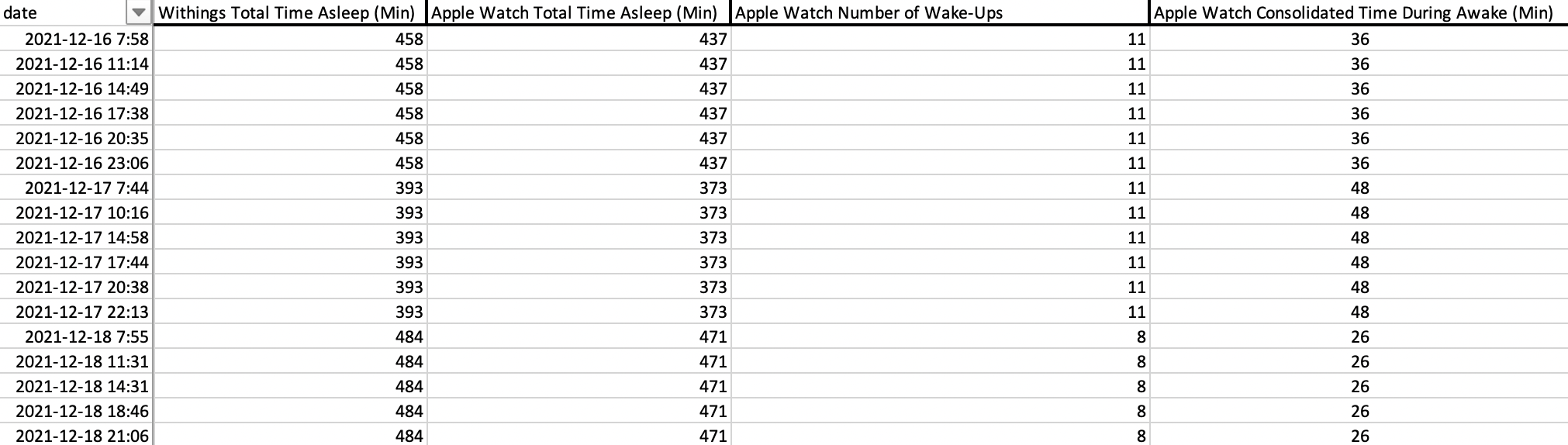


Figure A17: Snapshot of Sleep Features in the Dataset
