# Supplementary material for: Application of a mobile health data platform for public health surveillance: A case study in stress monitoring and prediction
Source: Digit Health. 2024 Jun 8;10:20552076241249931. doi: 10.1177/20552076241249931 (PMC11394344; doi:10.1177/20552076241249931)
Supplement: sj-docx-2-dhj-10.1177_20552076241249931 - Supplemental material for Application of a mobile health data platform for public health surveillance: A case study in stress monitoring and prediction [file sj-docx-2-dhj-10.1177_20552076241249931.docx]

## Supplementary Material

## Appendix 2 – Additional Tables

Table B1: Questionnaire Questions

| Questionnaire | Question | Answer Options |
| --- | --- | --- |
| DASS-21 | I found it hard to wind down; | 0 – Not at all  1 – To Some Degree  2 – To a Considerable Degree  3 – Very Much |
| DASS-21 | I felt that I was using a lot of nervous energy; | 0 – Not at all  1 – To Some Degree  2 – To a Considerable Degree  3 – Very Much |
| DASS-21 | I found myself getting agitated; | 0 – Not at all  1 – To Some Degree  2 – To a Considerable Degree  3 – Very Much |
| DASS-21 | I found it difficult to relax; | 0 – Not at all  1 – To Some Degree  2 – To a Considerable Degree  3 – Very Much |
| DASS-21 | I tended to over-react to situations; | 0 – Not at all  1 – To Some Degree  2 – To a Considerable Degree  3 – Very Much |
| DASS-21 | I was intolerant of anything that kept me from getting on with what I was doing; | 0 – Not at all  1 – To Some Degree  2 – To a Considerable Degree  3 – Very Much |
| DASS-21 | I felt that I was rather touchy; | 0 – Not at all  1 – To Some Degree  2 – To a Considerable Degree  3 – Very Much |
| Single-Item | Right now, I am… | 1 – Feeling Great  2 – Feeling Good  3 – A little stressed  4 – Definitely Stressed  5 – Stressed Out |

Table B2: Features used in the study

| **Manufacturer** | **Variable** | **Feature** | Description (unit) | Dataset |
| --- | --- | --- | --- | --- |
| Apple | Steps | Apple Watch Mean Steps | Mean of steps for the time interval | D, DA, DAW, SDA, SDAW |
| Apple | Steps | Apple Watch Max Steps | Maximum of steps for the time interval | D, DA, DAW, SDA, SDAW |
| Apple | Steps | Apple Watch Min Steps | Minimum HR for the time interval (bpm) | D, DA, DAW, SDA, SDAW |
| Apple | HR | Apple Watch Mean HR - Interval | Mean HR for the time interval (bpm) | D, DA, DAW, SDA, SDAW |
| Apple | HR | Apple Watch Max HR – Interval | Maximum HR for the time interval (bpm) | D, DA, DAW, SDA, SDAW |
| Apple | HR | Apple Watch Min HR - Interval | Minimum of steps for the time interval | D, DA, DAW, SDA, SDAW |
| Apple | HR | Short Term Mean | Mean HR for the millisecond time interval close to data collection (bpm) | D, DA, DAW, SDA, SDAW |
| Apple | HR | Short Term Max | Maximum HR for the millisecond time interval close to data collection (bpm) | D, DA, DAW, SDA, SDAW |
| Apple | HR | Short Term Min | Minimum HR for the millisecond time interval close to data collection (bpm) | D, DA, DAW, SDA, SDAW |
| Apple | HR | ECG_Mean HR | Mean of heart rate from ECG(ms) | D, DECG, DA, DAW, SDA, SDAW |
| Apple | HR | ECG_ SD HR | Standard deviation of instantaneous heart rate from ECG (1/min) | D, DECG, DA, DAW, SDA, SDAW |
| Apple | HR | ECG_Min HR | Minimum instantaneous heart rate calculated using 5 beat moving average from ECG(1/min) | D, DECG, DA, DAW, SDA, SDAW |
| Apple | HR | ECG_Max HR | Maximum instantaneous heart rate calculated using 5 beat moving average from ECG (1/min) | D, DECG, DA, DAW, SDA, SDAW |
| Empatica | HR | Empatica_Mean HR | Mean of heart rate from Empatica device (ms) | D, DEmpatica |
| Empatica | HR | Empatica_ SD HR | Standard deviation of instantaneous heart rate from Empatica device (1/min) | D, DEmpatica |
| Empatica | HR | Empatica_Min HR | Minimum instantaneous heart rate calculated using 5 beat moving average from Empatica device (1/min) | D, DEmpatica |
| Empatica | HR | Empatica_Max HR | Maximum instantaneous heart rate calculated using 5 beat moving average from Empatica device (1/min) | D, DEmpatica |
| Apple | HRV | HRV-1 | Heart rate variability collected as SDNN with the Apple Watch | D, DA, DAW, SDA, SDAW |
| Apple | HRV | ECG_PNS Index | Parasympathetic nervous system activity compared to normal resting values | D, DECG, DA, DAW, SDA, SDAW |
| Apple | HRV | ECG_SNS Index | Sympathetic nervous system activity compared to normal resting values | D, DECG, DA, DAW, SDA, SDAW |
| Apple | HRV | ECG_Stress Index | Square root of Baevsky’s stress index | D, DECG, DA, DAW, SDA, SDAW |
| Apple | HRV | ECG_Mean RR | Mean of R-R intervals (ms) | D, DECG, DA, DAW, SDA, SDAW |
| Apple | HRV | ECG_SDNN | Standard deviation of R-R intervals (ms) | D, DECG, DA, DAW, SDA, SDAW |
| Apple | HRV | ECG_RMSSD | Square root of the mean squared differences between successive RR intervals f(ms) | D, DECG, DA, DAW, SDA, SDAW |
| Apple | HRV | ECG_DC | Heart rate deceleration capacity (ms) | D, DECG, DA, DAW, SDA, SDAW |
| Apple | HRV | ECG_DCMod | Modified DC computer as a two-point difference (ms) | D, DECG, DA, DAW, SDA, SDAW |
| Apple | HRV | ECG_AC | Heart rate acceleration capacity (ms) | D, DECG, DA, DAW, SDA, SDAW |
| Apple | HRV | ECG_ACMod | Modified AC computer as a two-point difference (ms) | D, DECG, DA, DAW, SDA, SDAW |
| Apple | HRV | ECG_FFT LF | Fast Fourier Transform Low Frequency band components (Hz) | D, DECG, DA, DAW, SDA, SDAW |
| Apple | HRV | ECG_FFT HF | Fast Fourier Transform High Frequency band components (Hz) | D, DECG, DA, DAW, SDA, SDAW |
| Apple | HRV | ECG_AR LF | Autoregressive Low Frequency band components (Hz) | D, DECG, DA, DAW, SDA, SDAW |
| Apple | HRV | ECG_AR HF | Autoregressive High Frequency band components (Hz) | D, DECG, DA, DAW, SDA, SDAW |
| Apple | HRV | ECG_FFT Absolute Power LF | Fast Fourier Transform Absolute Power of Low Frequency band components (ms2) | D, DECG, DA, DAW, SDA, SDAW |
| Apple | HRV | ECG_FFT Absolute Power HF | Fast Fourier Transform Absolute Power of High Frequency band components (ms2) | D, DECG, DA, DAW, SDA, SDAW |
| Apple | HRV | ECG_AR Absolute Power LF | Autoregressive Absolute Power of Low Frequency band components (ms2) | D, DECG, DA, DAW, SDA, SDAW |
| Apple | HRV | ECG_AR Absolute Power HF | Autoregressive Absolute Power of High Frequency band components (ms2) | D, DECG, DA, DAW, SDA, SDAW |
| Apple | HRV | ECG_FFT Relative Power LF | Fast Fourier Transform Relative Power of Low Frequency band components (%) | D, DECG, DA, DAW, SDA, SDAW |
| Apple | HRV | ECG_FFT Relative Power HF | Fast Fourier Transform Relative Power of High Frequency band components (%) | D, DECG, DA, DAW, SDA, SDAW |
| Apple | HRV | ECG_AR Relative Power LF | Autoregressive Relative Power of Low Frequency band components (%) | D, DECG, DA, DAW, SDA, SDAW |
| Apple | HRV | ECG_AR Relative Power HF | Autoregressive Relative Power of High Frequency band components (%) | D, DECG, DA, DAW, SDA, SDAW |
| Apple | HRV | ECG_FFT Normalized Power LF | Fast Fourier Transform Normalized Power of Low Frequency band components (n.u) | D, DECG, DA, DAW, SDA, SDAW |
| Apple | HRV | ECG_FFT Normalized Power HF | Fast Fourier Transform Normalized Power of High Frequency band components (n.u) | D, DECG, DA, DAW, SDA, SDAW |
| Apple | HRV | ECG_FFT Total Power | Fast Fourier Transform Total Power (ms2) | D, DECG, DA, DAW, SDA, SDAW |
| Apple | HRV | ECG_FFT LF/HF | Fast Fourier Transform ratio between low and high frequency | D, DECG, DA, DAW, SDA, SDAW |
| Apple | HRV | ECG_AR Normalized Power LF | Autoregressive Normalized Power of Low Frequency band components (n.u) | D, DECG, DA, DAW, SDA, SDAW |
| Apple | HRV | ECG_AR Normalized Power HF | Autoregressive Normalized Power of High Frequency band components (n.u) | D, DECG, DA, DAW, SDA, SDAW |
| Apple | HRV | ECG_AR Total Power | Autoregressive Total Power (ms2) | D, DECG, DA, DAW, SDA, SDAW |
| Apple | HRV | ECG_AR LF/HF | Autoregressive ratio between low and high frequency | D, DECG, DA, DAW, SDA, SDAW |
| Apple | HRV | ECG_SD1 | The standard deviation perpendicular to the line-of-identity in Poincaré plot (ms) | D, DECG, DA, DAW, SDA, SDAW |
| Apple | HRV | ECG_SD2 | The standard deviation along the line-of-identity in Poincaré plot (ms) | D, DECG, DA, DAW, SDA, SDAW |
| Apple | HRV | ECG_SD2/SD1 | Ratio between SD2 and SD1 (ms) | D, DECG, DA, DAW, SDA, SDAW |
| Empatica | HRV | Empatica_PNS Index | Parasympathetic nervous system activity compared to normal resting values | D, DEmpatica |
| Empatica | HRV | Empatica_SNS Index | Sympathetic nervous system activity compared to normal resting values | D, DEmpatica |
| Empatica | HRV | Empatica_Stress Index | Square root of Baevsky’s stress index | D, DEmpatica |
| Empatica | HRV | Empatica_Mean RR | Mean of R-R intervals (ms) | D, DEmpatica |
| Empatica | HRV | Empatica_SDNN | Standard deviation of R-R intervals (ms) | D, DEmpatica |
| Empatica | HRV | Empatica_RMSSD | Square root of the mean squared differences between successive RR intervals (ms) | D, DEmpatica |
| Empatica | HRV | Empatica_DC | Heart rate deceleration capacity (ms) | D, DEmpatica |
| Empatica | HRV | Empatica_DCMod | Modified DC computer as a two-point difference (ms) | D, DEmpatica |
| Empatica | HRV | Empatica_AC | Heart rate acceleration capacity (ms) | D, DEmpatica |
| Empatica | HRV | Empatica_ACMod | Modified AC computer as a two-point difference (ms) | D, DEmpatica |
| Empatica | HRV | Empatica_FFT VLF | Fast Fourier Transform Very Low Frequency band components (Hz) | D, DEmpatica |
| Empatica | HRV | Empatica_FFT LF | Fast Fourier Transform Low Frequency band components (Hz) | D, DEmpatica |
| Empatica | HRV | Empatica_FFT HF | Fast Fourier Transform High Frequency band components (Hz) | D, DEmpatica |
| Empatica | HRV | Empatica_AR_VLF | Autoregressive Very Low Frequency band components (Hz) | D, DEmpatica |
| Empatica | HRV | Empatica_AR LF | Autoregressive Low Frequency band components (Hz) | D, DEmpatica |
| Empatica | HRV | Empatica_AR HF | Autoregressive High Frequency band components (Hz) | D, DEmpatica |
| Empatica | HRV | Empatica_FFT Absolute Power VLF | Fast Fourier Transform Absolute Power of Very Low Frequency band components (ms2) | D, DEmpatica |
| Empatica | HRV | Empatica_FFT Absolute Power LF | Fast Fourier Transform Absolute Power of Low Frequency band components (ms2) | D, DEmpatica |
| Empatica | HRV | Empatica_FFT Absolute Power HF | Fast Fourier Transform Absolute Power of High Frequency band components (ms2) | D, DEmpatica |
| Empatica | HRV | Empatica_AR Absolute Power VLF | Autoregressive Absolute Power of Very Low Frequency band components (ms2) | D, DEmpatica |
| Empatica | HRV | Empatica_AR Absolute Power LF | Autoregressive Absolute Power of Low Frequency band components (ms2) | D, DEmpatica |
| Empatica | HRV | Empatica_AR Absolute Power HF | Autoregressive Absolute Power of High Frequency band components (ms2) | D, DEmpatica |
| Empatica | HRV | Empatica_FFT Relative Power VLF | Fast Fourier Transform Relative Power of Very Low Frequency band components (%) | D, DEmpatica |
| Empatica | HRV | Empatica_FFT Relative Power LF | Fast Fourier Transform Relative Power of Low Frequency band components (%) | D, DEmpatica |
| Empatica | HRV | Empatica_FFT Relative Power HF | Fast Fourier Transform Relative Power of High Frequency band components (%) | D, DEmpatica |
| Empatica | HRV | Empatica_AR Relative Power VLF | Autoregressive Relative Power of Very Low Frequency band components (%) | D, DEmpatica |
| Empatica | HRV | Empatica_AR Relative Power LF | Autoregressive Relative Power of Low Frequency band components (%) | D, DEmpatica |
| Empatica | HRV | Empatica_AR Relative Power HF | Autoregressive Relative Power of High Frequency band components (%) | D, DEmpatica |
| Empatica | HRV | Empatica_FFT Normalized Power LF | Fast Fourier Transform Normalized Power of Low Frequency band components (n.u) | D, DEmpatica |
| Empatica | HRV | Empatica_FFT Normalized Power HF | Fast Fourier Transform Normalized Power of High Frequency band components (n.u) | D, DEmpatica |
| Empatica | HRV | Empatica_FFT Total Power | Fast Fourier Transform Total Power (ms2) | D, DEmpatica |
| Empatica | HRV | Empatica_FFT LF/HF | Fast Fourier Transform ratio between low and high frequency | D, DEmpatica |
| Empatica | HRV | Empatica_resp | Respiration rate (Hz) | D, DEmpatica |
| Empatica | HRV | Empatica_AR Normalized Power LF | Autoregressive Normalized Power of Low Frequency band components (n.u) | D, DEmpatica |
| Empatica | HRV | Empatica_AR Normalized Power HF | Autoregressive Normalized Power of High Frequency band components (n.u) | D, DEmpatica |
| Empatica | HRV | Empatica_AR Total Power | Autoregressive Total Power (ms2) | D, DEmpatica |
| Empatica | HRV | Empatica_AR LF/HF | Autoregressive ratio between low and high frequency | D, DEmpatica |
| Empatica | HRV | Empatica_SD1 | The standard deviation perpendicular to the line-of-identity in Poincaré plot (ms) | D, DEmpatica |
| Empatica | HRV | Empatica_SD2 | The standard deviation along the line-of-identity in Poincaré plot (ms) | D, DEmpatica |
| Empatica | HRV | Empatica_SD2/SD1 | Ratio between SD2 and SD1 (ms) | D, DEmpatica |
| Empatica | HRV | Empatica_ApEn | Approximate entropy | D, DEmpatica |
| Empatica | HRV | Empatica_SampEn | Sample entropy | D, DEmpatica |
| Empatica | HRV | Empatica_alpha1 | In detrended fluctuation, short term fluctuation slope | D, DEmpatica |
| Empatica | HRV | Empatica_alpha2 | In detrended fluctuation, long term fluctuation slope | D, DEmpatica |
| Empatica | HRV | Empatica_D2 | Correlation dimension | D, DEmpatica |
| Empatica | HRV | Empatica_Mean line length | Mean line length of the recurrent plot analysis | D, DEmpatica |
| Empatica | HRV | Empatica_Max line length | Max line length of the recurrent plot analysis | D, DEmpatica |
| Empatica | HRV | Empatica_REC | Recurrence rate of the recurrent plot analysis | D, DEmpatica |
| Empatica | HRV | Empatica_DET | Determinism of the recurrent plot analysis | D, DEmpatica |
| Empatica | HRV | Empatica_Shannon | Shannon entropy of the recurrent plot analysis | D, DEmpatica |
| Empatica | HRV | Empatica_MSE1 | Multiscale entropy for scale factor 1 | D, DEmpatica |
| Empatica | HRV | Empatica_MSE2 | Multiscale entropy for scale factor 2 | D, DEmpatica |
| Empatica | HRV | Empatica_MSE3 | Multiscale entropy for scale factor 3 | D, DEmpatica |
| Empatica | HRV | Empatica_MSE4 | Multiscale entropy for scale factor 4 | D, DEmpatica |
| Empatica | HRV | Empatica_MSE5 | Multiscale entropy for scale factor 5 | D, DEmpatica |
| Empatica | HRV | Empatica_MSE6 | Multiscale entropy for scale factor 6 | D, DEmpatica |
| Empatica | HRV | Empatica_MSE7 | Multiscale entropy for scale factor 7 | D, DEmpatica |
| Empatica | HRV | Empatica_MSE8 | Multiscale entropy for scale factor 8 | D, DEmpatica |
| Empatica | HRV | Empatica_MSE9 | Multiscale entropy for scale factor 9 | D, DEmpatica |
| Empatica | HRV | Empatica_MSE10 | Multiscale entropy for scale factor 10 | D, DEmpatica |
| Empatica | HRV | Empatica_MSE11 | Multiscale entropy for scale factor 11 | D, DEmpatica |
| Empatica | HRV | Empatica_MSE12 | Multiscale entropy for scale factor 12 | D, DEmpatica |
| Empatica | HRV | Empatica_MSE13 | Multiscale entropy for scale factor 13 | D, DEmpatica |
| Empatica | HRV | Empatica_MSE14 | Multiscale entropy for scale factor 14 | D, DEmpatica |
| Empatica | HRV | Empatica_MSE15 | Multiscale entropy for scale factor 15 | D, DEmpatica |
| Empatica | HRV | Empatica_MSE16 | Multiscale entropy for scale factor 16 | D, DEmpatica |
| Empatica | HRV | Empatica_MSE17 | Multiscale entropy for scale factor 17 | D, DEmpatica |
| Empatica | HRV | Empatica_MSE18 | Multiscale entropy for scale factor 18 | D, DEmpatica |
| Empatica | HRV | Empatica_MSE19 | Multiscale entropy for scale factor 19 | D, DEmpatica |
| Empatica | HRV | Empatica_MSE20 | Multiscale entropy for scale factor 20 | D, DEmpatica |
| Withings | Temperature | Temp | Temperature (Celsius) | DAW, DW, SDAW, SDW |
| Withings | Weight | Weight | Weight (kg) | DAW, DW, SDAW, SDW |
| Withings | Systolic blood pressure | sys | Systolic Blood Pressure (mmHg) | DAW, DW, SDAW, SDW |
| Withings | Diastolic blood pressure | dia | Diastolic Blood Pressure (mmHg) | DAW, DW, SDAW, SDW |
| Withings | Mean arterial pressure | MAP | Mean Arterial Pressure calculated as (sys + 3* dys)/3 (mmHg) | DAW, DW, SDAW, SDW |
| Apple | Sleep/Steps | Apple Watch Mean Steps (also included offset by: *t+2, t-2*) | Mean of steps for the night from last measure of previous day to first measure of day | SDA, SDAW, SDS |
| Apple | Sleep/Steps | Apple Watch Max Steps (also included offset by: *t+2, t-2*) | Maximum of steps from last measure of previous day to first measure of day | SDA, SDAW, SDS |
| Apple | Sleep/Steps | Apple Watch Min Steps (also included offset by: *t+2, t-2*) | Minimum HR for the time interval from last measure of previous day to first measure of day (bpm) | SDA, SDAW, SDS |
| Apple | Sleep/HR | Apple Watch Mean HR – Interval (also included offset by: *t+2, t-2, t+1, t-1*) | Mean HR from last measure of previous day to first measure of day (bpm) | SDA, SDAW, SDS |
| Apple | Sleep/HR | Apple Watch Max HR – Interval (also included offset by: *t+2, t-2, t+1, t-1*) | Maximum HR from last measure of previous day to first measure of day (bpm) | SDA, SDAW, SDS |
| Apple | Sleep/HR | Apple Watch Min HR – Interval (also included offset by: *t+2, t-2, t+1, t-1*) | Minimum of steps from last measure of previous day to first measure of day (bpm) | SDA, SDAW, SDS |
| Apple | Sleep | Apple Watch Total Time Asleep (also included offset by: *t+2, t-2*) | Total time asleep calculated with the Apple Watch (min) | SDA, SDAW, SDS |
| Apple | Sleep | Apple Watch Number of Wake-Ups (also included offset by: *t+2, t-2, t+1*) | Number of wake-ups in the night calculated with the Apple Watch | SDA, SDAW, SDS |
| Apple | Sleep | Apple Watch Consolidated Time During Awake (also included offset by: *t+2, t-2, t+1*) | Aggregated time duration of wake-ups (min) calculated with the Apple Watch | SDA, SDAW, SDS |
| Apple | Sleep | Apple Watch Total Time In Bed (also included offset by: *t+2, t-2, t-1*) | Total time spent in bed, awake or asleep (min) calculated with the Apple Watch | SDA, SDAW, SDS |
| Apple | Sleep | Apple Watch % of Time Asleep While In Bed (also included offset by: *t+2, t-2*) | Percentage of time spent asleep compared to total time in bed calculated with the Apple Watch | SDA, SDAW, SDS |
| Withings | Sleep | Withings Total Time Asleep (also included offset by: *t+2, t-2, t+1, t-1*) | Total time asleep calculated with Withings Sleep (min) | SDW, SDAW, SDS |
| Withings | Sleep | Withings Number of Wake-Ups (also included offset by: *t+2, t-2*) | Number of wake-ups in the night calculated with the Withings Sleep | SDW, SDAW, SDS |
| Withings | Sleep | Withings Consolidated Time During Awake (also included offset by: *t+2, t-2*) | Aggregated time duration of wake-ups calculated with the Withings Sleep (min) | SDW, SDAW, SDS |
| Withings | Sleep | Withings Total Time In Bed (also included offset by: *t+2, t-2*) | Total time spent in bed, awake or asleep calculated with the Apple Watch (min) | SDW, SDAW, SDS |
| Withings | Sleep | Withings % of Time Asleep While In Bed (also included offset by: *t+2, t-2*) | Percentage of time spent asleep compared to total time in bed calculated with the Withings Sleep (min) | SDW, SDAW, SDS |
| Withings | Sleep | Total Time Spent in Light Stage (also included offset by: *t+2, t-2*) | Time spent in light sleep stage calculated with Withings Sleep (min) | SDW, SDAW, SDS |
| Withings | Sleep | Total Time Spent in Deep Stage (also included offset by: *t+2, t-2*) | Time spent in deep sleep stage calculated with Withings Sleep (min) | SDW, SDAW, SDS |
| Withings | Sleep | Total Time Spent in REM Stage (also included offset by: *t+2, t-2*) | Time spent in REM sleep stage calculated with Withings Sleep (min) | SDW, SDAW, SDS |

Table B3: Participant Characteristics in Each Dataset

**Dataset with All Features (D), N = 22**

*Participants Frequency Percentage*

*Age*

18-24 5 23.

25-34 9 41

35-44 5 23

*Sex/Gender*

Male 7 32

Female 14 64

Gender Fluid 1 5

*SES*

Low (0-$30,000) 12 54

Medium ($30,000– $100,000) 10 46

High (Above $100,000) 0 0

Do not wish to disclose 0 0

*Profession*

Full-time 8 36

Part-time 1 5

Student 11 50

Self-employed/Other 1 5

Retired 1 5

*Health Status*

Healthy 16 73

Chronic Disease or Illness, 6 27

Prescription Drug Use,

Smoking or Alcohol

*Classes*

Stress 874 49

No Stress 926 51

**Dataset with Only ECG Features (DECG) / Dataset with Only Apple Features (DA), N = 42**

*Age*

18-24 12 29

25-34 13 31

35-44 12 29

*Sex/Gender*

Male 13 31

Female 28 67

Gender Fluid 1 2

*SES*

Low (0-$30,000) 19 45

Medium ($30,000– $100,000) 6 38

High (Above $100,000) 4 10

Do not wish to disclose 3 7

*Profession*

Full-time 20 48

Part-time 3 7

Student 16 38

Self-employed/Other 2 4

Retired 1 2

*Health Status*

Healthy 34 81

Chronic Disease or Illness, 8 19

Prescription Drug Use,

Smoking or Alcohol

*Classes*

Stress 1470 44

No Stress 1861 56

**Dataset with Apple and Withings Features (DAW), N = 41**

*Age*

18-24 12 29

25-34 13 32

35-44 11 27

*Sex/Gender*

Male 12 29

Female 28 68

Gender Fluid 1 2

*SES*

Low (0-$30,000) 19 46

Medium ($30,000– $100,000) 15 37

High (Above $100,000) 4 10

Do not wish to disclose 3 7

*Profession*

Full-time 19 46

Part-time 3 7

Student 16 39

Self-employed/Other 2 4

Retired 1 2

*Health Status*

Healthy 33 80

Chronic Disease or Illness, 8 20

Prescription Drug Use,

Smoking or Alcohol

*Classes*

Stress 1416 44

No Stress 1835 56

**Dataset with Only Withings Features (DW), N = 44**

*Age*

18-24 13 30

25-34 14 32

35-44 11 25

*Sex/Gender*

Male 13 30

Female 30 68

Gender Fluid 1 2

*SES*

Low (0-$30,000) 20 45

Medium ($30,000– $100,000) 17 39

High (Above $100,000) 4 9

Do not wish to disclose 3 7

*Profession*

Full-time 20 45

Part-time 5 11

Student 16 36

Self-employed/Other 2 5

Retired 1 3

*Health Status*

Healthy 35 80

Chronic Disease or Illness, 9 20

Prescription Drug Use,

Smoking or Alcohol

*Classes*

Stress 1485 43

No Stress 1986 57

**Dataset with Only Empatica Features (DEmpatica), N = 27**

*Age*

18-24 7 26

25-34 10 37

35-44 6 22

*Sex/Gender*

Male 8 30

Female 18 67

Gender Fluid 1 4

*SES*

Low (0-$30,000) 14 52

Medium ($30,000– $100,000) 13 48

High (Above $100,000) 0 0

Do not wish to disclose 0 0

*Profession*

Full-time 9 33

Part-time 4 15

Student 11 41

Self-employed/Other 2 8

Retired 1 4

*Health Status*

Healthy 20 74

Chronic Disease or Illness, 7 26

Prescription Drug Use,

Smoking or Alcohol

*Classes*

Stress 982 45

No Stress 1189 55

**Sleep Dataset with only Apple Features (SDA), N = 34**

*Age*

18-24 11 32

25-34 11 32

35-44 9 26

*Sex/Gender*

Male 10 29

Female 23 68

Gender Fluid 1 3

*SES*

Low (0-$30,000) 17 50

Medium ($30,000– $100,000) 14 41

High (Above $100,000) 0 0

Do not wish to disclose 3 9

*Profession*

Full-time 15 44

Part-time 1 3

Student 16 47

Self-employed/Other 2 6

Retired 0 0

*Health Status*

Healthy 28 82

Chronic Disease or Illness, 7 18

Prescription Drug Use,

Smoking or Alcohol

*Classes*

Stress 1192 44

No Stress 1543 56

**Sleep Dataset with Apple and Withings Features (SDAW), N = 27**

*Age*

18-24 8 30

25-34 9 33

35-44 7 26

*Sex/Gender*

Male 6 22

Female 20 74

Gender Fluid 1 4

*SES*

Low (0-$30,000) 13 48

Medium ($30,000– $100,000) 11 41

High (Above $100,000) 1 4

Do not wish to disclose 2 7

*Profession*

Full-time 12 44

Part-time 1 4

Student 12 44

Self-employed/Other 2 8

Retired 0 0

*Health Status*

Healthy 20 74

Chronic Disease or Illness, 7 26

Prescription Drug Use,

Smoking or Alcohol

*Classes*

Stress 894 42

No Stress 1245 58

**Sleep Dataset with Withings Features (SDW), N = 34**

*Age*

18-24 9 26

25-34 10 29

35-44 10 29

*Sex/Gender*

Male 9 26

Female 24 71

Gender Fluid 1 3

*SES*

Low (0-$30,000) 15 44

Medium ($30,000– $100,000) 6 38

High (Above $100,000) 4 12

Do not wish to disclose 2 6

*Profession*

Full-time 16 47

Part-time 3 9

Student 12 35

Self-employed/Other 2 6

Retired 1 3

*Health Status*

Healthy 8 82

Chronic Disease or Illness, 28 18

Prescription Drug Use,

Smoking or Alcohol

*Classes*

Stress 1136 42

No Stress 1536 58

**Sleep Dataset with Withings and Apple Only Sleep Features (SDS), N = 27**

*Age*

18-24 8 30

25-34 9 33

35-44 7 26

*Sex/Gender*

Male 6 22

Female 20 74

Gender Fluid 1 4

*SES*

Low (0-$30,000) 13 48

Medium ($30,000– $100,000) 11 41

High (Above $100,000) 1 4

Do not wish to disclose 2 7

*Profession*

Full-time 12 44

Part-time 1 4

Student 12 44

Self-employed/Other 2 8

Retired 0 0

*Health Status*

Healthy 7 26

Chronic Disease or Illness, 20 74

Prescription Drug Use,

Smoking or Alcohol

*Classes*

Stress 894 42

No Stress 1245 58

Table B4: Precision, Recall, Accuracy, F1-Score for Non-Sleep Datasets, Generalized

| **D** | **RF** | | | | | | **SVM** | | | **Support** |
| --- | --- | --- | --- | --- | --- | --- | --- | --- | --- | --- |
|  | **Items** | ***Precision*** | | ***Recall*** | | ***F1-Score*** | ***Precision*** | ***Recall*** | ***F1-Score*** | ***Support*** |
| Complete Dataset | *No Stress* | 0.67 | | 0.67 | | 0.67 | 0.66 | 0.65 | 0.66 | 185 |
|  | *Stress* | 0.65 | | 0.65 | | 0.65 | 0.64 | 0.65 | 0.64 | 175 |
|  | *Accuracy* | - | | - | | 0.66 | - | - | 0.65 | 360 |
|  | *Weighted Average* | 0.66 | | 0.66 | | 0.66 | 0.65 | 0.65 | 0.65 | 360 |
|  | *Macro Average* | 0.66 | | 0.66 | | 0.66 | 0.65 | 0.65 | 0.65 | 360 |
| Complete Dataset (SMOTE) | *No Stress* | 0.70 | | 0.67 | | 0.68 | 0.67 | 0.63 | 0.65 | 185 |
|  | *Stress* | 0.66 | | 0.69 | | 0.68 | 0.63 | 0.67 | 0.65 | 175 |
|  | *Accuracy* | - | | - | | 0.68 | - | - | 0.65 | 360 |
|  | *Weighted Average* | 0.68 | | 0.68 | | 0.68 | 0.65 | 0.65 | 0.65 | 360 |
|  | *Macro Average* | 0.68 | | 0.68 | | 0.68 | 0.65 | 0.65 | 0.65 | 360 |
| Gender - Male | *No Stress* | 0.69 | | 0.85 | | 0.76 | 0.71 | 0.82 | 0.76 | 67 |
|  | *Stress* | 0.72 | | 0.50 | | 0.59 | 0.71 | 0.58 | 0.64 | 52 |
|  | *Accuracy* |  | |  | | 0.70 |  |  | 0.71 | 119 |
|  | *Weighted Average* | 0.70 | | 0.70 | | 0.69 | 0.71 | 0.71 | 0.71 | 119 |
|  | *Macro Average* | 0.70 | | 0.68 | | 0.68 | 0.71 | 0.70 | 0.70 | 119 |
| Gender – Male (SMOTE) | *No Stress* | 0.66 | | 0.76 | | 0.71 | 0.67 | 0.73 | 0.70 | 67 |
|  | *Stress* | 0.62 | | 0.50 | | 0.55 | 0.61 | 0.54 | 0.57 | 52 |
|  | *Accuracy* |  | |  | | 0.65 |  |  | 0.65 | 119 |
|  | *Weighted Average* | 0.64 | | 0.65 | | 0.64 | 0.64 | 0.65 | 0.64 | 119 |
|  | *Macro Average* | 0.64 | | 0.63 | | 0.63 | 0.64 | 0.63 | 0.64 | 119 |
| Gender – Female | *No Stress* | 0.76 | | 0.52 | | 0.62 | 0.64 | 0.46 | 0.54 | 108 |
|  | *Stress* | 0.66 | | 0.85 | | 0.74 | 0.61 | 0.76 | 0.68 | 118 |
|  | *Accuracy* |  | |  | | 0.69 |  |  | 0.62 | 226 |
|  | *Weighted Average* | 0.71 | | 0.69 | | 0.68 | 0.62 | 0.62 | 0.61 | 226 |
|  | *Macro Average* | 0.71 | | 0.68 | | 0.68 | 0.62 | 0.61 | 0.61 | 226 |
| Gender – Female (SMOTE) | *No Stress* | 0.68 | | 0.56 | | 0.62 | 0.66 | 0.56 | 0.61 | 108 |
|  | *Stress* | 0.65 | | 0.75 | | 0.70 | 0.65 | 0.74 | 0.69 | 118 |
|  | *Accuracy* |  | |  | | 0.66 |  |  | 0.65 | 226 |
|  | *Weighted Average* | 0.67 | | 0.66 | | 0.66 | 0.66 | 0.65 | 0.65 | 226 |
|  | *Macro Average* | 0.67 | | 0.66 | | 0.66 | 0.66 | 0.65 | 0.65 | 226 |
| Employment - Student | *No Stress* | 0.65 | | 0.55 | | 0.60 | 0.66 | 0.60 | 0.63 | 53 |
|  | *Stress* | 0.57 | | 0.67 | | 0.61 | 0.59 | 0.64 | 0.61 | 63 |
|  | *Accuracy* |  | |  | | 0.61 |  |  | 0.62 | 116 |
|  | *Weighted Average* | 0.61 | | 0.61 | | 0.61 | 0.63 | 0.62 | 0.62 | 116 |
|  | *Macro Average* | 0.61 | | 0.61 | | 0.61 | 0.62 | 0.62 | 0.62 | 116 |
| Employment – Student (SMOTE) | *No Stress* | 0.65 | | 0.54 | | 0.59 | 0.66 | 0.59 | 0.63 | 53 |
|  | *Stress* | 0.56 | | 0.67 | | 0.61 | 0.59 | 0.65 | 0.62 | 63 |
|  | *Accuracy* |  | |  | | 0.60 |  |  | 0.62 | 116 |
|  | *Weighted Average* | 0.61 | | 0.60 | | 0.60 | 0.63 | 0.62 | 0.62 | 116 |
|  | *Macro Average* | 0.60 | | 0.60 | | 0.60 | 0.62 | 0.62 | 0.62 | 116 |
| Employment – Worker | *No Stress* | 0.63 | | 0.51 | | 0.57 | 0.58 | 0.57 | 0.57 | 76 |
|  | *Stress* | 0.64 | | 0.74 | | 0.68 | 0.63 | 0.65 | 0.64 | 88 |
|  | *Accuracy* |  | |  | | 0.63 |  |  | 0.61 | 164 |
|  | *Weighted Average* | 0.63 | | 0.63 | | 0.63 | 0.61 | 0.61 | 0.61 | 164 |
|  | *Macro Average* | 0.63 | | 0.63 | | 0.62 | 0.61 | 0.61 | 0.61 | 164 |
| Employment – Worker (SMOTE) | *No Stress* | 0.61 | | 0.59 | | 0.60 | 0.55 | 0.54 | 0.54 | 76 |
|  | *Stress* | 0.66 | | 0.67 | | 0.66 | 0.61 | 0.61 | 0.61 | 88 |
|  | *Accuracy* |  | |  | | 0.63 |  |  | 0.58 | 164 |
|  | *Weighted Average* | 0.63 | | 0.63 | | 0.63 | 0.58 | 0.58 | 0.58 | 164 |
|  | *Macro Average* | 0.63 | | 0.63 | | 0.63 | 0.58 | 0.58 | 0.58 | 164 |
| Income - Low | *No Stress* | 0.67 | | 0.74 | | 0.70 | 0.68 | 0.68 | 0.68 | 112 |
|  | *Stress* | 0.59 | | 0.50 | | 0.54 | 0.57 | 0.57 | 0.57 | 82 |
|  | *Accuracy* |  | |  | | 0.64 |  |  | 0.63 | 194 |
|  | *Weighted Average* | 0.63 | | 0.64 | | 0.63 | 0.63 | 0.63 | 0.63 | 194 |
|  | *Macro Average* | 0.63 | | 0.62 | | 0.62 | 0.63 | 0.63 | 0.63 | 194 |
| Income – Low (SMOTE) | *No Stress* | 0.68 | | 0.68 | | 0.68 | 0.64 | 0.91 | 0.75 | 112 |
|  | *Stress* | 0.57 | | 0.57 | | 0.57 | 0.71 | 0.30 | 0.43 | 82 |
|  | *Accuracy* |  | |  | | 0.63 |  |  | 0.65 | 194 |
|  | *Weighted Average* | 0.63 | | 0.63 | | 0.63 | 0.67 | 0.65 | 0.62 | 194 |
|  | *Macro Average* | 0.63 | | 0.63 | | 0.63 | 0.68 | 0.61 | 0.59 | 194 |
| Income – Medium High | *No Stress* | 0.82 | | 0.55 | | 0.66 | 0.82 | 0.49 | 0.62 | 73 |
|  | *Stress* | 0.72 | | 0.90 | | 0.80 | 0.70 | 0.91 | 0.79 | 94 |
|  | *Accuracy* |  | |  | | 0.75 |  |  | 0.73 | 167 |
|  | *Weighted Average* | 0.76 | | 0.75 | | 0.74 | 0.75 | 0.73 | 0.72 | 167 |
|  | *Macro Average* | 0.77 | | 0.73 | | 0.73 | 0.76 | 0.70 | 0.70 | 167 |
| Income – Medium High (SMOTE) | *No Stress* | 0.71 | | 0.62 | | 0.66 | 0.70 | 0.60 | 0.65 | 73 |
|  | *Stress* | 0.73 | | 0.81 | | 0.77 | 0.72 | 0.80 | 0.76 | 94 |
|  | *Accuracy* |  | |  | | 0.72 |  |  | 0.71 | 167 |
|  | *Weighted Average* | 0.72 | | 0.72 | | 0.72 | 0.71 | 0.71 | 0.71 | 167 |
|  | *Macro Average* | 0.72 | | 0.71 | | 0.71 | 0.71 | 0.70 | 0.70 | 167 |
| Age – 18-24 | *No Stress* | 0.67 | | 0.94 | | 0.78 | 0.65 | 1.00 | 0.79 | 52 |
|  | *Stress* | 0.57 | | 0.14 | | 0.23 | 0.00 | 0.00 | 0.00 | 28 |
|  | *Accuracy* |  | |  | | 0.66 |  |  | 0.65 | 80 |
|  | *Weighted Average* | 0.64 | | 0.66 | | 0.59 | 0.42 | 0.65 | 0.51 | 80 |
|  | *Macro Average* | 0.62 | | 0.54 | | 0.51 | 0.33 | 0.50 | 0.39 | 80 |
| Age – 18-24 (SMOTE) | *No Stress* | 0.67 | | 0.81 | | 0.73 | 0.65 | 1.00 | 0.79 | 52 |
|  | *Stress* | 0.41 | | 0.25 | | 0.31 | 0.00 | 0.00 | 0.00 | 28 |
|  | *Accuracy* |  | |  | | 0.61 |  |  | 0.65 | 80 |
|  | *Weighted Average* | 0.58 | | 0.61 | | 0.58 | 0.42 | 0.65 | 0.51 | 80 |
|  | *Macro Average* | 0.54 | | 0.53 | | 0.52 | 0.33 | 0.50 | 0.39 | 80 |
| Age 25-34 | *No Stress* | 0.56 | | 0.27 | | 0.36 | 0.56 | 0.41 | 0.47 | 56 |
|  | *Stress* | 0.67 | | 0.88 | | 0.76 | 0.71 | 0.81 | 0.76 | 97 |
|  | *Accuracy* |  | |  | | 0.65 |  |  | 0.67 | 153 |
|  | *Weighted Average* | 0.63 | | 0.65 | | 0.62 | 0.65 | 0.67 | 0.65 | 153 |
|  | *Macro Average* | 0.62 | | 0.57 | | 0.56 | 0.63 | 0.61 | 0.62 | 153 |
| Age 25-34 (SMOTE) | *No Stress* | 0.50 | | 0.45 | | 0.47 | 0.40 | 0.07 | 0.12 | 56 |
|  | *Stress* | 0.70 | | 0.74 | | 0.72 | 0.64 | 0.94 | 0.76 | 97 |
|  | *Accuracy* |  | |  | | 0.63 |  |  | 0.62 | 153 |
|  | *Weighted Average* | 0.63 | | 0.63 | | 0.63 | 0.55 | 0.62 | 0.53 | 153 |
|  | *Macro Average* | 0.60 | | 0.59 | | 0.60 | 0.52 | 0.50 | 0.44 | 153 |
| Age 35-44 | *No Stress* | 0.60 | | 0.78 | | 0.68 | 0.63 | 0.89 | 0.74 | 46 |
|  | *Stress* | 0.44 | | 0.25 | | 0.32 | 0.62 | 0.25 | 0.36 | 32 |
|  | *Accuracy* |  | |  | | 0.56 |  |  | 0.63 | 78 |
|  | *Weighted Average* | 0.54 | | 0.56 | | 0.53 | 0.62 | 0.63 | 0.58 | 78 |
|  | *Macro Average* | 0.52 | | 0.52 | | 0.50 | 0.62 | 0.57 | 0.55 | 78 |
| Age 35-44 (SMOTE) | *No Stress* | 0.62 | | 0.78 | | 0.69 | 0.67 | 0.80 | 0.73 | 46 |
|  | *Stress* | 0.50 | | 0.31 | | 0.38 | 0.61 | 0.44 | 0.51 | 32 |
|  | *Accuracy* |  | |  | | 0.59 |  |  | 0.65 | 78 |
|  | *Weighted Average* | 0.57 | | 0.59 | | 0.57 | 0.65 | 0.65 | 0.64 | 78 |
|  | *Macro Average* | 0.56 | | 0.55 | | 0.54 | 0.64 | 0.62 | 0.62 | 78 |
| Age 45-64 | *No Stress* | 0.65 | | 0.61 | | 0.63 | 0.75 | 0.67 | 0.71 | 18 |
|  | *Stress* | 0.59 | | 0.62 | | 0.61 | 0.67 | 0.75 | 0.71 | 16 |
|  | *Accuracy* |  | |  | | 0.62 |  |  | 0.71 | 34 |
|  | *Weighted Average* | 0.62 | | 0.62 | | 0.62 | 0.71 | 0.71 | 0.71 | 34 |
|  | *Macro Average* | 0.62 | | 0.62 | | 0.62 | 0.71 | 0.71 | 0.71 | 34 |
| Age 45-64 (SMOTE) | *No Stress* | 0.65 | | 0.61 | | 0.63 | 0.71 | 0.67 | 0.69 | 18 |
|  | *Stress* | 0.59 | | 0.62 | | 0.61 | 0.65 | 0.69 | 0.67 | 16 |
|  | *Accuracy* |  | |  | | 0.62 |  |  | 0.68 | 34 |
|  | *Weighted Average* | 0.62 | | 0.62 | | 0.62 | 0.68 | 0.68 | 0.68 | 34 |
|  | *Macro Average* | 0.62 | | 0.62 | | 0.62 | 0.68 | 0.68 | 0.68 | 34 |
| Healthy | *No Stress* | 0.68 | | 0.78 | | 0.72 | 0.69 | 0.73 | 0.71 | 139 |
|  | *Stress* | 0.69 | | 0.57 | | 0.63 | 0.67 | 0.62 | 0.65 | 120 |
|  | *Accuracy* |  | |  | | 0.68 |  |  | 0.68 | 259 |
|  | *Weighted Average* | 0.68 | | 0.68 | | 0.68 | 0.68 | 0.68 | 0.68 | 259 |
|  | *Macro Average* | 0.68 | | 0.68 | | 0.68 | 0.68 | 0.68 | 0.68 | 259 |
| Healthy - SMOTE | *No Stress* | 0.69 | | 0.71 | | 0.70 | 0.64 | 0.66 | 0.65 | 139 |
|  | *Stress* | 0.65 | | 0.62 | | 0.64 | 0.59 | 0.57 | 0.58 | 120 |
|  | *Accuracy* |  | |  | | 0.67 |  |  | 0.62 | 259 |
|  | *Weighted Average* | 0.67 | | 0.67 | | 0.67 | 0.62 | 0.62 | 0.62 | 259 |
|  | *Macro Average* | 0.67 | | 0.67 | | 0.67 | 0.62 | 0.62 | 0.62 | 259 |
| **DECG** | **RF** | | | | | | **SVM** | | |  |
|  | **Items** | ***Precision*** | | ***Recall*** | | ***F1-Score*** | ***Precision*** | ***Recall*** | ***F1-Score*** | **Support** |
| Complete Dataset | *No Stress* | 0.64 | | 0.74 | | 0.69 | 0.58 | 0.66 | 0.62 | 373 |
|  | *Stress* | 0.59 | | 0.48 | | 0.53 | 0.48 | 0.40 | 0.44 | 294 |
|  | *Accuracy* | - | | - | | 0.63 | - | - | 0.55 | 667 |
|  | *Weighted Average* | 0.62 | | 0.61 | | 0.61 | 0.54 | 0.55 | 0.54 | 667 |
|  | *Macro Average* | 0.62 | | 0.63 | | 0.62 | 0.53 | 0.53 | 0.53 | 667 |
| Complete Dataset (SMOTE) | *No Stress* | 0.65 | | 0.66 | | 0.66 | 0.59 | 0.76 | 0.67 | 373 |
|  | *Stress* | 0.56 | | 0.55 | | 0.56 | 0.52 | 0.34 | 0.41 | 294 |
|  | *Accuracy* | - | | - | | 0.61 | - | - | 0.57 | 667 |
|  | *Weighted Average* | 0.61 | | 0.61 | | 0.61 | 0.56 | 0.57 | 0.55 | 667 |
|  | *Macro Average* | 0.61 | | 0.61 | | 0.61 | 0.56 | 0.55 | 0.54 | 667 |
| Gender - Male | *No Stress* | 0.65 | | 0.73 | | 0.69 | 0.65 | 0.70 | 0.68 | 114 |
|  | *Stress* | 0.58 | | 0.49 | | 0.53 | 0.56 | 0.50 | 0.53 | 86 |
|  | *Accuracy* |  | |  | | 0.62 |  |  | 0.61 | 200 |
|  | *Weighted Average* | 0.62 | | 0.62 | | 0.62 | 0.61 | 0.61 | 0.61 | 200 |
|  | *Macro Average* | 0.61 | | 0.61 | | 0.61 | 0.60 | 0.60 | 0.60 | 200 |
| Gender – Male (SMOTE) | *No Stress* | 0.67 | | 0.67 | | 0.67 | 0.65 | 0.64 | 0.65 | 114 |
|  | *Stress* | 0.56 | | 0.57 | | 0.57 | 0.53 | 0.55 | 0.54 | 86 |
|  | *Accuracy* |  | |  | | 0.62 |  |  | 0.60 | 200 |
|  | *Weighted Average* | 0.63 | | 0.62 | | 0.63 | 0.60 | 0.60 | 0.60 | 200 |
|  | *Macro Average* | 0.62 | | 0.62 | | 0.62 | 0.59 | 0.59 | 0.59 | 200 |
| Gender – Female | *No Stress* | 0.65 | | 0.69 | | 0.67 | 0.63 | 0.65 | 0.64 | 247 |
|  | *Stress* | 0.60 | | 0.55 | | 0.57 | 0.56 | 0.53 | 0.55 | 204 |
|  | *Accuracy* |  | |  | | 0.63 |  |  | 0.60 | 451 |
|  | *Weighted Average* | 0.63 | | 0.63 | | 0.63 | 0.60 | 0.60 | 0.60 | 451 |
|  | *Macro Average* | 0.62 | | 0.62 | | 0.62 | 0.59 | 0.59 | 0.59 | 451 |
| Gender – Female (SMOTE) | *No Stress* | 0.66 | | 0.64 | | 0.65 | 0.61 | 0.58 | 0.59 | 247 |
|  | *Stress* | 0.58 | | 0.60 | | 0.59 | 0.52 | 0.55 | 0.53 | 204 |
|  | *Accuracy* |  | |  | | 0.62 |  |  | 0.57 | 451 |
|  | *Weighted Average* | 0.62 | | 0.62 | | 0.62 | 0.57 | 0.57 | 0.57 | 451 |
|  | *Macro Average* | 0.62 | | 0.62 | | 0.62 | 0.56 | 0.56 | 0.56 | 451 |
| Employment - Student | *No Stress* | 0.69 | | 0.58 | | 0.63 | 0.61 | 0.64 | 0.63 | 135 |
|  | *Stress* | 0.60 | | 0.71 | | 0.65 | 0.58 | 0.55 | 0.57 | 121 |
|  | *Accuracy* |  | |  | | 0.64 |  |  | 0.60 | 256 |
|  | *Weighted Average* | 0.65 | | 0.64 | | 0.64 | 0.60 | 0.60 | 0.60 | 256 |
|  | *Macro Average* | 0.65 | | 0.64 | | 0.64 | 0.60 | 0.60 | 0.60 | 256 |
| Employment – Student (SMOTE) | *No Stress* | 0.66 | | 0.59 | | 0.62 | 0.62 | 0.64 | 0.63 | 135 |
|  | *Stress* | 0.59 | | 0.67 | | 0.63 | 0.58 | 0.56 | 0.57 | 121 |
|  | *Accuracy* |  | |  | | 0.62 |  |  | 0.60 | 256 |
|  | *Weighted Average* | 0.63 | | 0.62 | | 0.62 | 0.60 | 0.60 | 0.60 | 256 |
|  | *Macro Average* | 0.63 | | 0.63 | | 0.62 | 0.60 | 0.60 | 0.60 | 256 |
| Employment – Worker | *No Stress* | 0.63 | | 0.75 | | 0.68 | 0.61 | 0.77 | 0.68 | 224 |
|  | *Stress* | 0.56 | | 0.43 | | 0.49 | 0.54 | 0.36 | 0.44 | 171 |
|  | *Accuracy* |  | |  | | 0.61 |  |  | 0.59 | 395 |
|  | *Weighted Average* | 0.60 | | 0.59 | | 0.59 | 0.58 | 0.59 | 0.57 | 395 |
|  | *Macro Average* | 0.60 | | 0.61 | | 0.60 | 0.58 | 0.57 | 0.56 | 395 |
| Employment – Worker (SMOTE) | *No Stress* | 0.64 | | 0.64 | | 0.64 | 0.57 | 0.81 | 0.67 | 224 |
|  | *Stress* | 0.53 | | 0.54 | | 0.53 | 0.44 | 0.20 | 0.27 | 171 |
|  | *Accuracy* |  | |  | | 0.59 |  |  | 0.54 | 395 |
|  | *Weighted Average* | 0.60 | | 0.59 | | 0.60 | 0.51 | 0.54 | 0.50 | 395 |
|  | *Macro Average* | 0.59 | | 0.59 | | 0.59 | 0.51 | 0.50 | 0.47 | 395 |
| Income - Low | *No Stress* | 0.63 | | 0.79 | | 0.70 | 0.64 | 0.67 | 0.65 | 224 |
|  | *Stress* | 0.47 | | 0.29 | | 0.36 | 0.45 | 0.43 | 0.44 | 171 |
|  | *Accuracy* |  | |  | | 0.59 |  |  | 0.57 | 395 |
|  | *Weighted Average* | 0.57 | | 0.59 | | 0.57 | 0.57 | 0.57 | 0.57 | 395 |
|  | *Macro Average* | 0.55 | | 0.54 | | 0.53 | 0.55 | 0.55 | 0.55 | 395 |
| Income – Low (SMOTE) | *No Stress* | 0.63 | | 0.65 | | 0.64 | 0.60 | 0.82 | 0.69 | 224 |
|  | *Stress* | 0.43 | | 0.41 | | 0.42 | 0.38 | 0.17 | 0.24 | 171 |
|  | *Accuracy* |  | |  | | 0.56 |  |  | 0.56 | 395 |
|  | *Weighted Average* | 0.55 | | 0.56 | | 0.55 | 0.51 | 0.56 | 0.51 | 395 |
|  | *Macro Average* | 0.53 | | 0.53 | | 0.53 | 0.49 | 0.49 | 0.46 | 395 |
| Income – Medium High | *No Stress* | 0.73 | | 0.58 | | 0.65 | 0.64 | 0.57 | 0.60 | 166 |
|  | *Stress* | 0.64 | | 0.77 | | 0.70 | 0.60 | 0.67 | 0.63 | 159 |
|  | *Accuracy* |  | |  | | 0.68 |  |  | 0.62 | 325 |
|  | *Weighted Average* | 0.69 | | 0.68 | | 0.67 | 0.62 | 0.62 | 0.61 | 325 |
|  | *Macro Average* | 0.68 | | 0.68 | | 0.67 | 0.62 | 0.62 | 0.61 | 325 |
| Income – Medium High (SMOTE) | *No Stress* | 0.70 | | 0.63 | | 0.66 | 0.62 | 0.49 | 0.55 | 166 |
|  | *Stress* | 0.65 | | 0.72 | | 0.68 | 0.56 | 0.68 | 0.62 | 159 |
|  | *Accuracy* |  | |  | | 0.67 |  |  | 0.58 | 325 |
|  | *Weighted Average* | 0.68 | | 0.67 | | 0.67 | 0.59 | 0.59 | 0.58 | 325 |
|  | *Macro Average* | 0.68 | | 0.67 | | 0.67 | 0.59 | 0.58 | 0.58 | 325 |
| Age – 18-24 | *No Stress* | 0.68 | | 0.84 | | 0.75 | 0.67 | 0.71 | 0.69 | 166 |
|  | *Stress* | 0.61 | | 0.39 | | 0.47 | 0.50 | 0.46 | 0.48 | 159 |
|  | *Accuracy* |  | |  | | 0.66 |  |  | 0.61 | 325 |
|  | *Weighted Average* | 0.65 | | 0.66 | | 0.65 | 0.61 | 0.61 | 0.61 | 325 |
|  | *Macro Average* | 0.65 | | 0.61 | | 0.61 | 0.59 | 0.58 | 0.58 | 325 |
| Age – 18-24 (SMOTE) | *No Stress* | 0.66 | | 0.66 | | 0.66 | 0.63 | 0.85 | 0.72 | 166 |
|  | *Stress* | 0.47 | | 0.47 | | 0.47 | 0.48 | 0.22 | 0.30 | 159 |
|  | *Accuracy* |  | |  | | 0.59 |  |  | 0.61 | 325 |
|  | *Weighted Average* | 0.59 | | 0.59 | | 0.59 | 0.57 | 0.61 | 0.56 | 325 |
|  | *Macro Average* | 0.57 | | 0.57 | | 0.57 | 0.56 | 0.54 | 0.51 | 325 |
| Age 25-34 | *No Stress* | 0.63 | | 0.35 | | 0.45 | 0.58 | 0.38 | 0.46 | 91 |
|  | *Stress* | 0.64 | | 0.85 | | 0.73 | 0.64 | 0.80 | 0.71 | 124 |
|  | *Accuracy* |  | |  | | 0.64 |  |  | 0.62 | 215 |
|  | *Weighted Average* | 0.63 | | 0.60 | | 0.59 | 0.62 | 0.62 | 0.61 | 215 |
|  | *Macro Average* | 0.63 | | 0.60 | | 0.59 | 0.61 | 0.59 | 0.59 | 215 |
| Age 25-34 (SMOTE) | *No Stress* | 0.54 | | 0.48 | | 0.51 | 0.43 | 0.44 | 0.44 | 91 |
|  | *Stress* | 0.65 | | 0.70 | | 0.67 | 0.59 | 0.58 | 0.58 | 124 |
|  | *Accuracy* |  | |  | | 0.61 |  |  | 0.52 | 215 |
|  | *Weighted Average* | 0.60 | | 0.61 | | 0.61 | 0.52 | 0.52 | 0.52 | 215 |
|  | *Macro Average* | 0.60 | | 0.59 | | 0.59 | 0.51 | 0.51 | 0.51 | 215 |
| Age 35-44 | *No Stress* | 0.67 | | 0.84 | | 0.75 | 0.66 | 0.69 | 0.67 | 113 |
|  | *Stress* | 0.57 | | 0.34 | | 0.42 | 0.46 | 0.42 | 0.44 | 71 |
|  | *Accuracy* |  | |  | | 0.65 |  |  | 0.59 | 184 |
|  | *Weighted Average* | 0.63 | | 0.65 | | 0.62 | 0.58 | 0.59 | 0.58 | 184 |
|  | *Macro Average* | 0.62 | | 0.59 | | 0.58 | 0.56 | 0.56 | 0.56 | 184 |
| Age 35-44 (SMOTE) | *No Stress* | 0.69 | | 0.72 | | 0.70 | 0.62 | 0.83 | 0.71 | 113 |
|  | *Stress* | 0.52 | | 0.49 | | 0.51 | 0.42 | 0.20 | 0.27 | 71 |
|  | *Accuracy* |  | |  | | 0.63 |  |  | 0.59 | 184 |
|  | *Weighted Average* | 0.63 | | 0.63 | | 0.63 | 0.55 | 0.59 | 0.54 | 184 |
|  | *Macro Average* | 0.61 | | 0.60 | | 0.61 | 0.52 | 0.51 | 0.49 | 184 |
| Healthy | *No Stress* | 0.64 | | 0.75 | | 0.69 | 0.62 | 0.72 | 0.66 | 308 |
|  | *Stress* | 0.55 | | 0.42 | | 0.48 | 0.50 | 0.39 | 0.44 | 224 |
|  | *Accuracy* |  | |  | | 0.61 |  |  | 0.58 | 532 |
|  | *Weighted Average* | 0.60 | | 0.61 | | 0.60 | 0.57 | 0.58 | 0.57 | 532 |
|  | *Macro Average* | 0.59 | | 0.58 | | 0.58 | 0.56 | 0.56 | 0.55 | 532 |
| Healthy (SMOTE) | *No Stress* | 0.68 | | 0.68 | | 0.68 | 0.64 | 0.65 | 0.64 | 308 |
|  | *Stress* | 0.56 | | 0.55 | | 0.55 | 0.50 | 0.49 | 0.50 | 224 |
|  | *Accuracy* |  | |  | | 0.63 |  |  | 0.58 | 532 |
|  | *Weighted Average* | 0.63 | | 0.63 | | 0.63 | 0.58 | 0.58 | 0.58 | 532 |
|  | *Macro Average* | 0.62 | | 0.62 | | 0.62 | 0.57 | 0.57 | 0.57 | 532 |
| **DA** | **RF** | | | | | | **SVM** | | |  |
|  | **Items** | ***Precision*** | | ***Recall*** | | ***F1-Score*** | ***Precision*** | ***Recall*** | ***F1-Score*** | ***Support*** |
| Complete Dataset | *No Stress* | 0.66 | | 0.72 | | 0.69 | 0.62 | 0.73 | 0.67 | 373 |
|  | *Stress* | 0.60 | | 0.53 | | 0.56 | 0.56 | 0.44 | 0.49 | 294 |
|  | *Accuracy* | - | | - | | 0.65 | - | - | 0.60 | 667 |
|  | *Weighted Average* | 0.63 | | 0.64 | | 0.63 | 0.60 | 0.60 | 0.59 | 667 |
|  | *Macro Average* | 0.63 | | 0.63 | | 0.63 | 0.59 | 0.58 | 0.58 | 667 |
| Complete Dataset (SMOTE) | *No Stress* | 0.69 | | 0.64 | | 0.66 | 0.59 | 0.61 | 0.60 | 373 |
|  | *Stress* | 0.58 | | 0.63 | | 0.60 | 0.48 | 0.46 | 0.47 | 294 |
|  | *Accuracy* | - | | - | | 0.63 | - | - | 0.54 | 667 |
|  | *Weighted Average* | 0.64 | | 0.63 | | 0.64 | 0.54 | 0.54 | 0.54 | 667 |
|  | *Macro Average* | 0.63 | | 0.63 | | 0.63 | 0.53 | 0.53 | 0.53 | 667 |
| Gender - Male | *No Stress* | 0.67 | | 0.76 | | 0.71 | 0.65 | 0.71 | 0.68 | 114 |
|  | *Stress* | 0.61 | | 0.50 | | 0.55 | 0.57 | 0.50 | 0.53 | 86 |
|  | *Accuracy* |  | |  | | 0.65 |  |  | 0.62 | 200 |
|  | *Weighted Average* | 0.65 | | 0.65 | | 0.64 | 0.62 | 0.62 | 0.62 | 200 |
|  | *Macro Average* | 0.64 | | 0.63 | | 0.63 | 0.61 | 0.61 | 0.61 | 200 |
| Gender – Male (SMOTE) | *No Stress* | 0.68 | | 0.72 | | 0.70 | 0.63 | 0.64 | 0.64 | 114 |
|  | *Stress* | 0.60 | | 0.56 | | 0.58 | 0.52 | 0.51 | 0.51 | 86 |
|  | *Accuracy* |  | |  | | 0.65 |  |  | 0.58 | 200 |
|  | *Weighted Average* | 0.65 | | 0.65 | | 0.65 | 0.58 | 0.58 | 0.58 | 200 |
|  | *Macro Average* | 0.64 | | 0.64 | | 0.64 | 0.58 | 0.58 | 0.58 | 200 |
| Gender – Female | *No Stress* | 0.62 | | 0.68 | | 0.65 | 0.57 | 0.63 | 0.60 | 247 |
|  | *Stress* | 0.56 | | 0.50 | | 0.53 | 0.49 | 0.43 | 0.46 | 204 |
|  | *Accuracy* |  | |  | | 0.60 |  |  | 0.54 | 451 |
|  | *Weighted Average* | 0.60 | | 0.60 | | 0.60 | 0.54 | 0.54 | 0.54 | 451 |
|  | *Macro Average* | 0.59 | | 0.59 | | 0.59 | 0.53 | 0.53 | 0.53 | 451 |
| Gender – Female (SMOTE) | *No Stress* | 0.64 | | 0.56 | | 0.60 | 0.63 | 0.57 | 0.60 | 247 |
|  | *Stress* | 0.54 | | 0.62 | | 0.58 | 0.53 | 0.59 | 0.56 | 204 |
|  | *Accuracy* |  | |  | | 0.59 |  |  | 0.58 | 451 |
|  | *Weighted Average* | 0.60 | | 0.59 | | 0.59 | 0.58 | 0.58 | 0.58 | 451 |
|  | *Macro Average* | 0.59 | | 0.59 | | 0.59 | 0.58 | 0.58 | 0.58 | 451 |
| Employment - Student | *No Stress* | 0.71 | | 0.58 | | 0.64 | 0.57 | 0.55 | 0.56 | 135 |
|  | *Stress* | 0.61 | | 0.74 | | 0.67 | 0.52 | 0.54 | 0.53 | 121 |
|  | *Accuracy* |  | |  | | 0.65 |  |  | 0.54 | 256 |
|  | *Weighted Average* | 0.66 | | 0.65 | | 0.65 | 0.54 | 0.54 | 0.54 | 256 |
|  | *Macro Average* | 0.66 | | 0.66 | | 0.65 | 0.54 | 0.54 | 0.54 | 256 |
| Employment – Student (SMOTE) | *No Stress* | 0.71 | | 0.52 | | 0.60 | 0.60 | 0.60 | 0.60 | 135 |
|  | *Stress* | 0.59 | | 0.77 | | 0.67 | 0.55 | 0.55 | 0.55 | 121 |
|  | *Accuracy* |  | |  | | 0.64 |  |  | 0.58 | 256 |
|  | *Weighted Average* | 0.65 | | 0.64 | | 0.63 | 0.58 | 0.58 | 0.58 | 256 |
|  | *Macro Average* | 0.65 | | 0.64 | | 0.63 | 0.58 | 0.58 | 0.58 | 256 |
| Employment – Worker | *No Stress* | 0.66 | | 0.75 | | 0.71 | 0.62 | 0.65 | 0.63 | 224 |
|  | *Stress* | 0.61 | | 0.50 | | 0.55 | 0.51 | 0.49 | 0.50 | 171 |
|  | *Accuracy* |  | |  | | 0.64 |  |  | 0.58 | 395 |
|  | *Weighted Average* | 0.64 | | 0.64 | | 0.64 | 0.58 | 0.58 | 0.58 | 395 |
|  | *Macro Average* | 0.63 | | 0.63 | | 0.63 | 0.57 | 0.57 | 0.57 | 395 |
| Employment – Worker (SMOTE) | *No Stress* | 0.66 | | 0.66 | | 0.66 | 0.60 | 0.62 | 0.61 | 224 |
|  | *Stress* | 0.56 | | 0.56 | | 0.56 | 0.48 | 0.45 | 0.46 | 171 |
|  | *Accuracy* |  | |  | | 0.62 |  |  | 0.55 | 395 |
|  | *Weighted Average* | 0.62 | | 0.62 | | 0.62 | 0.55 | 0.55 | 0.55 | 395 |
|  | *Macro Average* | 0.61 | | 0.61 | | 0.61 | 0.54 | 0.54 | 0.54 | 395 |
| Income - Low | *No Stress* | 0.64 | | 0.84 | | 0.73 | 0.63 | 0.73 | 0.68 | 224 |
|  | *Stress* | 0.52 | | 0.27 | | 0.36 | 0.44 | 0.32 | 0.37 | 171 |
|  | *Accuracy* |  | |  | | 0.62 |  |  | 0.57 | 395 |
|  | *Weighted Average* | 0.59 | | 0.62 | | 0.58 | 0.55 | 0.57 | 0.56 | 395 |
|  | *Macro Average* | 0.58 | | 0.56 | | 0.54 | 0.53 | 0.53 | 0.52 | 395 |
| Income – Low (SMOTE) | *No Stress* | 0.67 | | 0.69 | | 0.68 | 0.64 | 0.67 | 0.66 | 224 |
|  | *Stress* | 0.50 | | 0.47 | | 0.48 | 0.45 | 0.42 | 0.44 | 171 |
|  | *Accuracy* |  | |  | | 0.61 |  |  | 0.57 | 395 |
|  | *Weighted Average* | 0.60 | | 0.61 | | 0.60 | 0.57 | 0.57 | 0.57 | 395 |
|  | *Macro Average* | 0.58 | | 0.58 | | 0.58 | 0.55 | 0.55 | 0.55 | 395 |
| Income – Medium High | *No Stress* | 0.69 | | 0.63 | | 0.66 | 0.60 | 0.64 | 0.62 | 166 |
|  | *Stress* | 0.65 | | 0.70 | | 0.67 | 0.59 | 0.55 | 0.57 | 159 |
|  | *Accuracy* |  | |  | | 0.67 |  |  | 0.60 | 325 |
|  | *Weighted Average* | 0.67 | | 0.67 | | 0.67 | 0.60 | 0.60 | 0.60 | 325 |
|  | *Macro Average* | 0.67 | | 0.67 | | 0.67 | 0.60 | 0.60 | 0.60 | 325 |
| Income – Medium High (SMOTE) | *No Stress* | 0.68 | | 0.61 | | 0.65 | 0.60 | 0.61 | 0.60 | 166 |
|  | *Stress* | 0.64 | | 0.70 | | 0.67 | 0.59 | 0.58 | 0.58 | 159 |
|  | *Accuracy* |  | |  | | 0.66 |  |  | 0.59 | 325 |
|  | *Weighted Average* | 0.66 | | 0.66 | | 0.66 | 0.59 | 0.59 | 0.59 | 325 |
|  | *Macro Average* | 0.66 | | 0.66 | | 0.66 | 0.59 | 0.59 | 0.59 | 325 |
| Age – 18-24 | *No Stress* | 0.64 | | 0.81 | | 0.72 | 0.66 | 0.66 | 0.66 | 166 |
|  | *Stress* | 0.50 | | 0.29 | | 0.37 | 0.46 | 0.46 | 0.46 | 159 |
|  | *Accuracy* |  | |  | | 0.61 |  |  | 0.58 | 325 |
|  | *Weighted Average* | 0.59 | | 0.61 | | 0.58 | 0.58 | 0.58 | 0.58 | 325 |
|  | *Macro Average* | 0.57 | | 0.55 | | 0.54 | 0.56 | 0.56 | 0.56 | 325 |
| Age – 18-24 (SMOTE) | *No Stress* | 0.63 | | 0.65 | | 0.64 | 0.65 | 0.63 | 0.64 | 166 |
|  | *Stress* | 0.43 | | 0.40 | | 0.41 | 0.45 | 0.47 | 0.46 | 159 |
|  | *Accuracy* |  | |  | | 0.56 |  |  | 0.57 | 325 |
|  | *Weighted Average* | 0.55 | | 0.56 | | 0.56 | 0.57 | 0.57 | 0.57 | 325 |
|  | *Macro Average* | 0.53 | | 0.53 | | 0.53 | 0.55 | 0.55 | 0.55 | 325 |
| Age 25-34 | *No Stress* | 0.65 | | 0.35 | | 0.46 | 0.58 | 0.34 | 0.43 | 91 |
|  | *Stress* | 0.64 | | 0.86 | | 0.74 | 0.63 | 0.82 | 0.71 | 124 |
|  | *Accuracy* |  | |  | | 0.65 |  |  | 0.62 | 215 |
|  | *Weighted Average* | 0.65 | | 0.65 | | 0.62 | 0.61 | 0.62 | 0.59 | 215 |
|  | *Macro Average* | 0.65 | | 0.61 | | 0.60 | 0.61 | 0.58 | 0.57 | 215 |
| Age 25-34 (SMOTE) | *No Stress* | 0.60 | | 0.52 | | 0.56 | 0.50 | 0.46 | 0.48 | 91 |
|  | *Stress* | 0.68 | | 0.75 | | 0.71 | 0.63 | 0.66 | 0.64 | 124 |
|  | *Accuracy* |  | |  | | 0.65 |  |  | 0.58 | 215 |
|  | *Weighted Average* | 0.65 | | 0.65 | | 0.65 | 0.57 | 0.58 | 0.57 | 215 |
|  | *Macro Average* | 0.64 | | 0.63 | | 0.63 | 0.56 | 0.56 | 0.56 | 215 |
| Age 35-44 | *No Stress* | 0.67 | | 0.89 | | 0.77 | 0.66 | 0.82 | 0.74 | 113 |
|  | *Stress* | 0.65 | | 0.31 | | 0.42 | 0.55 | 0.34 | 0.42 | 71 |
|  | *Accuracy* |  | |  | | 0.67 |  |  | 0.64 | 184 |
|  | *Weighted Average* | 0.66 | | 0.67 | | 0.63 | 0.60 | 0.58 | 0.58 | 184 |
|  | *Macro Average* | 0.66 | | 0.60 | | 0.59 | 0.60 | 0.64 | 0.61 | 184 |
| Age 35-44 (SMOTE) | *No Stress* | 0.73 | | 0.75 | | 0.74 | 0.63 | 0.69 | 0.66 | 113 |
|  | *Stress* | 0.58 | | 0.55 | | 0.57 | 0.42 | 0.35 | 0.38 | 71 |
|  | *Accuracy* |  | |  | | 0.67 |  |  | 0.56 | 184 |
|  | *Weighted Average* | 0.67 | | 0.67 | | 0.67 | 0.55 | 0.56 | 0.55 | 184 |
|  | *Macro Average* | 0.65 | | 0.65 | | 0.65 | 0.52 | 0.52 | 0.52 | 184 |
| Healthy | *No Stress* | 0.65 | | 0.79 | | 0.71 | 0.62 | 0.77 | 0.69 | 308 |
|  | *Stress* | 0.59 | | 0.42 | | 0.49 | 0.54 | 0.36 | 0.43 | 224 |
|  | *Accuracy* |  | |  | | 0.63 |  |  | 0.60 | 532 |
|  | *Weighted Average* | 0.63 | | 0.63 | | 0.62 | 0.59 | 0.60 | 0.58 | 532 |
|  | *Macro Average* | 0.62 | | 0.60 | | 0.60 | 0.58 | 0.57 | 0.56 | 532 |
| Healthy (SMOTE) | *No Stress* | 0.68 | | 0.71 | | 0.69 | 0.65 | 0.57 | 0.61 | 308 |
|  | *Stress* | 0.57 | | 0.54 | | 0.56 | 0.49 | 0.58 | 0.53 | 224 |
|  | *Accuracy* |  | |  | | 0.64 |  |  | 0.57 | 532 |
|  | *Weighted Average* | 0.63 | | 0.64 | | 0.64 | 0.58 | 0.57 | 0.57 | 532 |
|  | *Macro Average* | 0.63 | | 0.62 | | 0.62 | 0.57 | 0.57 | 0.57 | 532 |
| **DAW** | **RF** | | | | | | **SVM** | | |  |
|  | **Items** | ***Precision*** | ***Recall*** | | ***F1-Score*** | | ***Precision*** | ***Recall*** | ***F1-Score*** | ***Support*** |
| Complete Dataset | *No Stress* | 0.69 | 0.82 | | 0.75 | | 0.64 | 0.69 | 0.66 | 367 |
|  | *Stress* | 0.70 | 0.52 | | 0.60 | | 0.55 | 0.50 | 0.53 | 284 |
|  | *Accuracy* | - | - | | 0.69 | | - | - | 0.61 | 651 |
|  | *Weighted Average* | 0.69 | 0.69 | | 0.68 | | 0.60 | 0.60 | 0.60 | 651 |
|  | *Macro Average* | 0.69 | 0.67 | | 0.67 | | 0.60 | 0.61 | 0.60 | 651 |
| Complete Dataset (SMOTE) | *No Stress* | 0.72 | 0.77 | | 0.74 | | 0.64 | 0.69 | 0.67 | 367 |
|  | *Stress* | 0.67 | 0.60 | | 0.64 | | 0.56 | 0.51 | 0.53 | 284 |
|  | *Accuracy* | - | - | | 0.70 | | - | - | 0.61 | 651 |
|  | *Weighted Average* | 0.70 | 0.70 | | 0.70 | | 0.60 | 0.60 | 0.60 | 651 |
|  | *Macro Average* | 0.69 | 0.69 | | 0.69 | | 0.61 | 0.61 | 0.61 | 651 |
| Gender - Male | *No Stress* | 0.74 | 0.92 | | 0.82 | | 0.74 | 0.80 | 0.77 | 109 |
|  | *Stress* | 0.81 | 0.52 | | 0.63 | | 0.67 | 0.59 | 0.62 | 75 |
|  | *Accuracy* |  |  | | 0.76 | |  |  | 0.71 | 184 |
|  | *Weighted Average* | 0.77 | 0.76 | | 0.74 | | 0.71 | 0.71 | 0.71 | 184 |
|  | *Macro Average* | 0.77 | 0.72 | | 0.73 | | 0.70 | 0.69 | 0.70 | 184 |
| Gender – Male (SMOTE) | *No Stress* | 0.75 | 0.82 | | 0.78 | | 0.72 | 0.83 | 0.77 | 109 |
|  | *Stress* | 0.69 | 0.60 | | 0.64 | | 0.68 | 0.53 | 0.60 | 75 |
|  | *Accuracy* |  |  | | 0.73 | |  |  | 0.71 | 184 |
|  | *Weighted Average* | 0.73 | 0.73 | | 0.72 | | 0.70 | 0.68 | 0.68 | 184 |
|  | *Macro Average* | 0.72 | 0.71 | | 0.71 | | 0.70 | 0.71 | 0.70 | 184 |
| Gender – Female | *No Stress* | 0.68 | 0.79 | | 0.73 | | 0.65 | 0.70 | 0.68 | 247 |
|  | *Stress* | 0.68 | 0.54 | | 0.60 | | 0.61 | 0.55 | 0.58 | 204 |
|  | *Accuracy* |  |  | | 0.68 | |  |  | 0.63 | 451 |
|  | *Weighted Average* | 0.68 | 0.68 | | 0.67 | | 0.63 | 0.63 | 0.63 | 451 |
|  | *Macro Average* | 0.68 | 0.67 | | 0.67 | | 0.63 | 0.63 | 0.63 | 451 |
| Gender – Female (SMOTE) | *No Stress* | 0.70 | 0.68 | | 0.69 | | 0.61 | 0.67 | 0.64 | 247 |
|  | *Stress* | 0.62 | 0.64 | | 0.63 | | 0.55 | 0.49 | 0.51 | 204 |
|  | *Accuracy* |  |  | | 0.66 | |  |  | 0.59 | 451 |
|  | *Weighted Average* | 0.66 | 0.66 | | 0.66 | | 0.58 | 0.58 | 0.58 | 451 |
|  | *Macro Average* | 0.66 | 0.66 | | 0.66 | | 0.58 | 0.59 | 0.58 | 451 |
| Employment - Student | *No Stress* | 0.69 | 0.61 | | 0.65 | | 0.66 | 0.66 | 0.66 | 135 |
|  | *Stress* | 0.62 | 0.70 | | 0.66 | | 0.62 | 0.63 | 0.63 | 121 |
|  | *Accuracy* |  |  | | 0.65 | |  |  | 0.64 | 256 |
|  | *Weighted Average* | 0.66 | 0.65 | | 0.65 | | 0.64 | 0.64 | 0.64 | 256 |
|  | *Macro Average* | 0.66 | 0.65 | | 0.65 | | 0.64 | 0.64 | 0.64 | 256 |
| Employment – Student (SMOTE) | *No Stress* | 0.69 | 0.52 | | 0.59 | | 0.62 | 0.69 | 0.65 | 135 |
|  | *Stress* | 0.58 | 0.74 | | 0.65 | | 0.61 | 0.55 | 0.57 | 121 |
|  | *Accuracy* |  |  | | 0.62 | |  |  | 0.62 | 256 |
|  | *Weighted Average* | 0.64 | 0.62 | | 0.62 | | 0.62 | 0.62 | 0.61 | 256 |
|  | *Macro Average* | 0.63 | 0.63 | | 0.62 | | 0.62 | 0.61 | 0.61 | 256 |
| Employment – Worker | *No Stress* | 0.69 | 0.81 | | 0.75 | | 0.68 | 0.74 | 0.71 | 219 |
|  | *Stress* | 0.66 | 0.50 | | 0.57 | | 0.59 | 0.51 | 0.55 | 160 |
|  | *Accuracy* |  |  | | 0.68 | |  |  | 0.65 | 379 |
|  | *Weighted Average* | 0.68 | 0.68 | | 0.67 | | 0.64 | 0.65 | 0.64 | 379 |
|  | *Macro Average* | 0.68 | 0.66 | | 0.66 | | 0.64 | 0.63 | 0.63 | 379 |
| Employment – Worker (SMOTE) | *No Stress* | 0.71 | 0.74 | | 0.73 | | 0.67 | 0.74 | 0.71 | 219 |
|  | *Stress* | 0.63 | 0.59 | | 0.61 | | 0.59 | 0.51 | 0.55 | 160 |
|  | *Accuracy* |  |  | | 0.68 | |  |  | 0.64 | 379 |
|  | *Weighted Average* | 0.68 | 0.68 | | 0.68 | | 0.63 | 0.63 | 0.63 | 379 |
|  | *Macro Average* | 0.67 | 0.67 | | 0.67 | | 0.64 | 0.64 | 0.64 | 379 |
| Income - Low | *No Stress* | 0.64 | 0.85 | | 0.73 | | 0.67 | 0.79 | 0.73 | 162 |
|  | *Stress* | 0.50 | 0.25 | | 0.33 | | 0.53 | 0.38 | 0.44 | 101 |
|  | *Accuracy* |  |  | | 0.62 | |  |  | 0.63 | 263 |
|  | *Weighted Average* | 0.59 | 0.62 | | 0.58 | | 0.62 | 0.63 | 0.62 | 263 |
|  | *Macro Average* | 0.57 | 0.55 | | 0.53 | | 0.60 | 0.58 | 0.58 | 263 |
| Income – Low (SMOTE) | *No Stress* | 0.68 | 0.74 | | 0.71 | | 0.66 | 0.73 | 0.69 | 162 |
|  | *Stress* | 0.52 | 0.45 | | 0.48 | | 0.48 | 0.39 | 0.43 | 101 |
|  | *Accuracy* |  |  | | 0.63 | |  |  | 0.60 | 263 |
|  | *Weighted Average* | 0.62 | 0.63 | | 0.62 | | 0.59 | 0.60 | 0.59 | 263 |
|  | *Macro Average* | 0.60 | 0.59 | | 0.59 | | 0.57 | 0.56 | 0.56 | 263 |
| Income – Medium High | *No Stress* | 0.75 | 0.68 | | 0.71 | | 0.66 | 0.68 | 0.67 | 161 |
|  | *Stress* | 0.68 | 0.76 | | 0.72 | | 0.64 | 0.62 | 0.63 | 148 |
|  | *Accuracy* |  |  | | 0.72 | |  |  | 0.65 | 309 |
|  | *Weighted Average* | 0.72 | 0.72 | | 0.72 | | 0.65 | 0.65 | 0.65 | 309 |
|  | *Macro Average* | 0.72 | 0.72 | | 0.72 | | 0.65 | 0.65 | 0.65 | 309 |
| Income – Medium High (SMOTE) | *No Stress* | 0.75 | 0.65 | | 0.70 | | 0.69 | 0.68 | 0.68 | 161 |
|  | *Stress* | 0.67 | 0.76 | | 0.71 | | 0.66 | 0.67 | 0.66 | 148 |
|  | *Accuracy* |  |  | | 0.71 | |  |  | 0.67 | 309 |
|  | *Weighted Average* | 0.71 | 0.71 | | 0.70 | | 0.67 | 0.67 | 0.67 | 309 |
|  | *Macro Average* | 0.71 | 0.71 | | 0.71 | | 0.67 | 0.67 | 0.67 | 309 |
| Age – 18-24 | *No Stress* | 0.68 | 0.85 | | 0.75 | | 0.67 | 0.79 | 0.72 | 113 |
|  | *Stress* | 0.60 | 0.36 | | 0.45 | | 0.54 | 0.39 | 0.45 | 72 |
|  | *Accuracy* |  |  | | 0.66 | |  |  | 0.63 | 185 |
|  | *Weighted Average* | 0.65 | 0.66 | | 0.64 | | 0.62 | 0.63 | 0.62 | 185 |
|  | *Macro Average* | 0.64 | 0.61 | | 0.60 | | 0.60 | 0.59 | 0.59 | 185 |
| Age – 18-24 (SMOTE) | *No Stress* | 0.71 | 0.74 | | 0.72 | | 0.68 | 0.79 | 0.73 | 113 |
|  | *Stress* | 0.56 | 0.51 | | 0.54 | | 0.56 | 0.42 | 0.48 | 72 |
|  | *Accuracy* |  |  | | 0.65 | |  |  | 0.64 | 185 |
|  | *Weighted Average* | 0.65 | 0.65 | | 0.65 | | 0.63 | 0.64 | 0.63 | 185 |
|  | *Macro Average* | 0.63 | 0.63 | | 0.63 | | 0.62 | 0.60 | 0.60 | 185 |
| Age 25-34 | *No Stress* | 0.73 | 0.52 | | 0.61 | | 0.57 | 0.58 | 0.58 | 91 |
|  | *Stress* | 0.71 | 0.86 | | 0.78 | | 0.69 | 0.68 | 0.68 | 124 |
|  | *Accuracy* |  |  | | 0.72 | |  |  | 0.64 | 215 |
|  | *Weighted Average* | 0.72 | 0.72 | | 0.71 | | 0.64 | 0.64 | 0.64 | 215 |
|  | *Macro Average* | 0.72 | 0.69 | | 0.69 | | 0.63 | 0.63 | 0.63 | 215 |
| Age 25-34 (SMOTE) | *No Stress* | 0.64 | 0.54 | | 0.59 | | 0.54 | 0.45 | 0.49 | 91 |
|  | *Stress* | 0.70 | 0.78 | | 0.74 | | 0.64 | 0.72 | 0.68 | 124 |
|  | *Accuracy* |  |  | | 0.68 | |  |  | 0.60 | 215 |
|  | *Weighted Average* | 0.68 | 0.68 | | 0.67 | | 0.60 | 0.60 | 0.60 | 215 |
|  | *Macro Average* | 0.67 | 0.66 | | 0.66 | | 0.59 | 0.58 | 0.58 | 215 |
| Age 35-44 | *No Stress* | 0.71 | 0.91 | | 0.79 | | 0.76 | 0.83 | 0.80 | 108 |
|  | *Stress* | 0.66 | 0.32 | | 0.43 | | 0.64 | 0.53 | 0.58 | 60 |
|  | *Accuracy* |  |  | | 0.70 | |  |  | 0.73 | 168 |
|  | *Weighted Average* | 0.69 | 0.70 | | 0.66 | | 0.72 | 0.73 | 0.72 | 168 |
|  | *Macro Average* | 0.68 | 0.61 | | 0.61 | | 0.70 | 0.68 | 0.69 | 168 |
| Age 35-44 (SMOTE) | *No Stress* | 0.75 | 0.84 | | 0.79 | | 0.69 | 0.81 | 0.74 | 108 |
|  | *Stress* | 0.63 | 0.48 | | 0.55 | | 0.49 | 0.33 | 0.40 | 60 |
|  | *Accuracy* |  |  | | 0.71 | |  |  | 0.64 | 168 |
|  | *Weighted Average* | 0.70 | 0.71 | | 0.70 | | 0.61 | 0.64 | 0.62 | 168 |
|  | *Macro Average* | 0.69 | 0.66 | | 0.67 | | 0.59 | 0.57 | 0.57 | 168 |
| Healthy | *No Stress* | 0.67 | 0.82 | | 0.74 | | 0.68 | 0.79 | 0.73 | 303 |
|  | *Stress* | 0.62 | 0.42 | | 0.50 | | 0.61 | 0.46 | 0.52 | 213 |
|  | *Accuracy* |  |  | | 0.66 | |  |  | 0.66 | 516 |
|  | *Weighted Average* | 0.65 | 0.66 | | 0.64 | | 0.65 | 0.65 | 0.64 | 516 |
|  | *Macro Average* | 0.64 | 0.62 | | 0.62 | | 0.64 | 0.63 | 0.63 | 516 |
| Healthy (SMOTE) | *No Stress* | 0.67 | 0.70 | | 0.68 | | 0.66 | 0.66 | 0.66 | 303 |
|  | *Stress* | 0.54 | 0.51 | | 0.53 | | 0.52 | 0.52 | 0.52 | 213 |
|  | *Accuracy* |  |  | | 0.62 | |  |  | 0.60 | 516 |
|  | *Weighted Average* | 0.62 | 0.62 | | 0.62 | | 0.59 | 0.59 | 0.59 | 516 |
|  | *Macro Average* | 0.61 | 0.60 | | 0.60 | | 0.60 | 0.60 | 0.60 | 516 |

| **DW** | **RF** | | | | **SVM** | | |  |
| --- | --- | --- | --- | --- | --- | --- | --- | --- |
|  | **Items** | ***Precision*** | ***Recall*** | ***F1-Score*** | ***Precision*** | ***Recall*** | ***F1-Score*** | ***Support*** |
| Complete Dataset | *No Stress* | 0.72 | 0.81 | 0.76 | 0.66 | 0.76 | 0.71 | 398 |
|  | *Stress* | 0.69 | 0.57 | 0.62 | 0.60 | 0.48 | 0.54 | 297 |
|  | *Accuracy* | - | - | 0.71 | - | - | 0.64 | 695 |
|  | *Weighted Average* | 0.70 | 0.71 | 0.70 | 0.64 | 0.64 | 0.64 | 695 |
|  | *Macro Average* | 0.70 | 0.69 | 0.69 | 0.63 | 0.62 | 0.62 | 695 |
| Complete Dataset (SMOTE) | *No Stress* | 0.72 | 0.68 | 0.70 | 0.71 | 0.64 | 0.67 | 398 |
|  | *Stress* | 0.60 | 0.64 | 0.62 | 0.57 | 0.65 | 0.61 | 297 |
|  | *Accuracy* | - | - | 0.66 | - | - | 0.64 | 695 |
|  | *Weighted Average* | 0.66 | 0.66 | 0.66 | 0.65 | 0.64 | 0.65 | 695 |
|  | *Macro Average* | 0.67 | 0.66 | 0.66 | 0.64 | 0.65 | 0.64 | 695 |
| Gender - Male | *No Stress* | 0.71 | 0.87 | 0.78 | 0.72 | 0.89 | 0.80 | 124 |
|  | *Stress* | 0.67 | 0.42 | 0.51 | 0.71 | 0.45 | 0.56 | 77 |
|  | *Accuracy* |  |  | 0.70 |  |  | 0.72 | 201 |
|  | *Weighted Average* | 0.69 | 0.70 | 0.68 | 0.72 | 0.72 | 0.70 | 201 |
|  | *Macro Average* | 0.69 | 0.64 | 0.65 | 0.72 | 0.67 | 0.68 | 201 |
| Gender – Male (SMOTE) | *No Stress* | 0.71 | 0.74 | 0.72 | 0.72 | 0.69 | 0.70 | 124 |
|  | *Stress* | 0.55 | 0.51 | 0.53 | 0.53 | 0.57 | 0.55 | 77 |
|  | *Accuracy* |  |  | 0.65 |  |  | 0.64 | 201 |
|  | *Weighted Average* | 0.65 | 0.65 | 0.65 | 0.65 | 0.64 | 0.64 | 201 |
|  | *Macro Average* | 0.63 | 0.62 | 0.63 | 0.63 | 0.63 | 0.63 | 201 |
| Gender – Female | *No Stress* | 0.65 | 0.76 | 0.70 | 0.61 | 0.72 | 0.66 | 251 |
|  | *Stress* | 0.63 | 0.50 | 0.56 | 0.55 | 0.43 | 0.48 | 203 |
|  | *Accuracy* |  |  | 0.65 |  |  | 0.59 | 454 |
|  | *Weighted Average* | 0.64 | 0.65 | 0.64 | 0.58 | 0.59 | 0.58 | 454 |
|  | *Macro Average* | 0.64 | 0.63 | 0.63 | 0.58 | 0.57 | 0.57 | 454 |
| Gender – Female (SMOTE) | *No Stress* | 0.67 | 0.72 | 0.69 | 0.65 | 0.67 | 0.66 | 251 |
|  | *Stress* | 0.62 | 0.56 | 0.59 | 0.58 | 0.56 | 0.57 | 203 |
|  | *Accuracy* |  |  | 0.65 |  |  | 0.62 | 454 |
|  | *Weighted Average* | 0.65 | 0.65 | 0.65 | 0.62 | 0.62 | 0.62 | 454 |
|  | *Macro Average* | 0.64 | 0.64 | 0.64 | 0.62 | 0.62 | 0.62 | 454 |
| Employment - Student | *No Stress* | 0.70 | 0.61 | 0.65 | 0.68 | 0.65 | 0.67 | 135 |
|  | *Stress* | 0.62 | 0.70 | 0.66 | 0.63 | 0.66 | 0.65 | 121 |
|  | *Accuracy* |  |  | 0.66 |  |  | 0.66 | 256 |
|  | *Weighted Average* | 0.66 | 0.66 | 0.66 | 0.66 | 0.66 | 0.66 | 256 |
|  | *Macro Average* | 0.66 | 0.65 | 0.65 | 0.66 | 0.66 | 0.66 | 256 |
| Employment – Student (SMOTE) | *No Stress* | 0.68 | 0.58 | 0.63 | 0.66 | 0.52 | 0.58 | 135 |
|  | *Stress* | 0.60 | 0.70 | 0.65 | 0.57 | 0.70 | 0.63 | 121 |
|  | *Accuracy* |  |  | 0.64 |  |  | 0.61 | 256 |
|  | *Weighted Average* | 0.64 | 0.64 | 0.64 | 0.61 | 0.61 | 0.60 | 256 |
|  | *Macro Average* | 0.64 | 0.64 | 0.64 | 0.62 | 0.61 | 0.60 | 256 |
| Employment – Worker | *No Stress* | 0.69 | 0.85 | 0.76 | 0.68 | 0.79 | 0.73 | 249 |
|  | *Stress* | 0.68 | 0.47 | 0.55 | 0.61 | 0.46 | 0.52 | 174 |
|  | *Accuracy* |  |  | 0.69 |  |  | 0.65 | 423 |
|  | *Weighted Average* | 0.69 | 0.69 | 0.68 | 0.64 | 0.63 | 0.63 | 423 |
|  | *Macro Average* | 0.69 | 0.66 | 0.66 | 0.65 | 0.65 | 0.64 | 423 |
| Employment – Worker (SMOTE) | *No Stress* | 0.70 | 0.74 | 0.72 | 0.71 | 0.63 | 0.67 | 249 |
|  | *Stress* | 0.60 | 0.55 | 0.57 | 0.54 | 0.63 | 0.59 | 174 |
|  | *Accuracy* |  |  | 0.66 |  |  | 0.63 | 423 |
|  | *Weighted Average* | 0.65 | 0.65 | 0.65 | 0.63 | 0.63 | 0.63 | 423 |
|  | *Macro Average* | 0.66 | 0.66 | 0.66 | 0.63 | 0.63 | 0.63 | 423 |
| Income - Low | *No Stress* | 0.66 | 0.77 | 0.71 | 0.67 | 0.74 | 0.71 | 189 |
|  | *Stress* | 0.52 | 0.39 | 0.44 | 0.52 | 0.44 | 0.48 | 121 |
|  | *Accuracy* |  |  | 0.62 |  |  | 0.62 | 310 |
|  | *Weighted Average* | 0.61 | 0.62 | 0.61 | 0.60 | 0.59 | 0.59 | 310 |
|  | *Macro Average* | 0.59 | 0.58 | 0.58 | 0.61 | 0.62 | 0.62 | 310 |
| Income – Low (SMOTE) | *No Stress* | 0.65 | 0.61 | 0.63 | 0.67 | 0.57 | 0.61 | 189 |
|  | *Stress* | 0.45 | 0.49 | 0.47 | 0.46 | 0.57 | 0.51 | 121 |
|  | *Accuracy* |  |  | 0.56 |  |  | 0.57 | 310 |
|  | *Weighted Average* | 0.57 | 0.56 | 0.57 | 0.56 | 0.57 | 0.56 | 310 |
|  | *Macro Average* | 0.55 | 0.55 | 0.55 | 0.59 | 0.57 | 0.57 | 310 |
| Income – Medium High | *No Stress* | 0.75 | 0.74 | 0.74 | 0.71 | 0.73 | 0.72 | 182 |
|  | *Stress* | 0.70 | 0.72 | 0.71 | 0.67 | 0.66 | 0.66 | 157 |
|  | *Accuracy* |  |  | 0.73 |  |  | 0.69 | 339 |
|  | *Weighted Average* | 0.73 | 0.73 | 0.73 | 0.69 | 0.69 | 0.69 | 339 |
|  | *Macro Average* | 0.73 | 0.73 | 0.73 | 0.65 | 0.65 | 0.65 | 339 |
| Income – Medium High (SMOTE) | *No Stress* | 0.73 | 0.67 | 0.70 | 0.72 | 0.68 | 0.70 | 182 |
|  | *Stress* | 0.65 | 0.71 | 0.68 | 0.65 | 0.69 | 0.67 | 157 |
|  | *Accuracy* |  |  | 0.69 |  |  | 0.69 | 339 |
|  | *Weighted Average* | 0.69 | 0.69 | 0.69 | 0.69 | 0.69 | 0.69 | 339 |
|  | *Macro Average* | 0.69 | 0.69 | 0.69 | 0.69 | 0.69 | 0.69 | 339 |
| Age – 18-24 | *No Stress* | 0.64 | 0.89 | 0.74 | 0.65 | 0.82 | 0.73 | 122 |
|  | *Stress* | 0.52 | 0.20 | 0.29 | 0.51 | 0.30 | 0.38 | 76 |
|  | *Accuracy* |  |  | 0.62 |  |  | 0.62 | 198 |
|  | *Weighted Average* | 0.59 | 0.62 | 0.57 | 0.60 | 0.62 | 0.59 | 198 |
|  | *Macro Average* | 0.58 | 0.54 | 0.51 | 0.58 | 0.56 | 0.55 | 198 |
| Age – 18-24 (SMOTE) | *No Stress* | 0.64 | 0.69 | 0.66 | 0.64 | 0.61 | 0.62 | 122 |
|  | *Stress* | 0.42 | 0.37 | 0.39 | 0.42 | 0.46 | 0.44 | 76 |
|  | *Accuracy* |  |  | 0.57 |  |  | 0.55 | 198 |
|  | *Weighted Average* | 0.55 | 0.57 | 0.56 | 0.53 | 0.53 | 0.53 | 198 |
|  | *Macro Average* | 0.53 | 0.53 | 0.53 | 0.56 | 0.55 | 0.55 | 198 |
| Age 25-34 | *No Stress* | 0.65 | 0.35 | 0.46 | 0.58 | 0.34 | 0.43 | 91 |
|  | *Stress* | 0.64 | 0.86 | 0.74 | 0.63 | 0.82 | 0.71 | 124 |
|  | *Accuracy* |  |  | 0.65 |  |  | 0.62 | 215 |
|  | *Weighted Average* | 0.65 | 0.65 | 0.62 | 0.61 | 0.62 | 0.59 | 215 |
|  | *Macro Average* | 0.65 | 0.61 | 0.60 | 0.61 | 0.58 | 0.57 | 215 |
| Age 25-34 (SMOTE) | *No Stress* | 0.60 | 0.52 | 0.56 | 0.50 | 0.46 | 0.48 | 91 |
|  | *Stress* | 0.68 | 0.75 | 0.71 | 0.63 | 0.66 | 0.64 | 124 |
|  | *Accuracy* |  |  | 0.65 |  |  | 0.58 | 215 |
|  | *Weighted Average* | 0.65 | 0.65 | 0.65 | 0.57 | 0.58 | 0.57 | 215 |
|  | *Macro Average* | 0.64 | 0.63 | 0.63 | 0.56 | 0.56 | 0.56 | 215 |
| Age 35-44 | *No Stress* | 0.71 | 0.87 | 0.78 | 0.72 | 0.88 | 0.79 | 108 |
|  | *Stress* | 0.61 | 0.37 | 0.46 | 0.64 | 0.38 | 0.48 | 60 |
|  | *Accuracy* |  |  | 0.69 |  |  | 0.70 | 168 |
|  | *Weighted Average* | 0.68 | 0.69 | 0.67 | 0.69 | 0.70 | 0.68 | 168 |
|  | *Macro Average* | 0.66 | 0.62 | 0.62 | 0.68 | 0.63 | 0.64 | 168 |
| Age 35-44 (SMOTE) | *No Stress* | 0.74 | 0.79 | 0.76 | 0.69 | 0.69 | 0.69 | 108 |
|  | *Stress* | 0.57 | 0.50 | 0.53 | 0.44 | 0.43 | 0.44 | 60 |
|  | *Accuracy* |  |  | 0.68 |  |  | 0.60 | 168 |
|  | *Weighted Average* | 0.68 | 0.68 | 0.68 | 0.60 | 0.60 | 0.60 | 168 |
|  | *Macro Average* | 0.65 | 0.64 | 0.65 | 0.56 | 0.56 | 0.56 | 168 |
| Healthy | *No Stress* | 0.68 | 0.77 | 0.72 | 0.66 | 0.80 | 0.72 | 327 |
|  | *Stress* | 0.58 | 0.47 | 0.52 | 0.56 | 0.38 | 0.45 | 220 |
|  | *Accuracy* |  |  | 0.65 |  |  | 0.63 | 547 |
|  | *Weighted Average* | 0.64 | 0.65 | 0.64 | 0.62 | 0.63 | 0.61 | 547 |
|  | *Macro Average* | 0.63 | 0.62 | 0.62 | 0.61 | 0.59 | 0.58 | 547 |
| Healthy (SMOTE) | *No Stress* | 0.69 | 0.68 | 0.68 | 0.66 | 0.65 | 0.66 | 327 |
|  | *Stress* | 0.54 | 0.55 | 0.54 | 0.49 | 0.50 | 0.50 | 220 |
|  | *Accuracy* |  |  | 0.63 |  |  | 0.59 | 547 |
|  | *Weighted Average* | 0.63 | 0.63 | 0.63 | 0.59 | 0.59 | 0.59 | 547 |
|  | *Macro Average* | 0.61 | 0.62 | 0.61 | 0.58 | 0.58 | 0.58 | 547 |
| **DEmpatica** | **RF** | | | | **SVM** | | |  |
|  | **Items** | ***Precision*** | ***Recall*** | ***F1-Score*** | ***Precision*** | ***Recall*** | ***F1-Score*** | **Support** |
| Complete Dataset | *No Stress* | 0.64 | 0.79 | 0.71 | 0.63 | 0.67 | 0.65 | 238 |
|  | *Stress* | 0.65 | 0.47 | 0.55 | 0.57 | 0.53 | 0.55 | 197 |
|  | *Accuracy* | - | - | 0.65 | - | - | 0.61 | 435 |
|  | *Weighted Average* | 0.65 | 0.63 | 0.63 | 0.60 | 0.61 | 0.61 | 435 |
|  | *Macro Average* | 0.65 | 0.65 | 0.64 | 0.60 | 0.60 | 0.60 | 435 |
| Complete Dataset (SMOTE) | *No Stress* | 0.68 | 0.69 | 0.68 | 0.64 | 0.67 | 0.65 | 238 |
|  | *Stress* | 0.62 | 0.60 | 0.61 | 0.58 | 0.55 | 0.56 | 197 |
|  | *Accuracy* | - | - | 0.65 | - | - | 0.61 | 435 |
|  | *Weighted Average* | 0.65 | 0.65 | 0.65 | 0.61 | 0.61 | 0.61 | 435 |
|  | *Macro Average* | 0.65 | 0.65 | 0.65 | 0.61 | 0.61 | 0.61 | 435 |
| Gender - Male | *No Stress* | 0.69 | 0.87 | 0.77 | 0.74 | 0.83 | 0.78 | 82 |
|  | *Stress* | 0.67 | 0.41 | 0.51 | 0.68 | 0.56 | 0.61 | 54 |
|  | *Accuracy* |  |  | 0.68 |  |  | 0.72 | 136 |
|  | *Weighted Average* | 0.68 | 0.68 | 0.66 | 0.72 | 0.72 | 0.71 | 136 |
|  | *Macro Average* | 0.68 | 0.64 | 0.64 | 0.71 | 0.69 | 0.70 | 136 |
| Gender – Male (SMOTE) | *No Stress* | 0.73 | 0.78 | 0.75 | 0.66 | 0.91 | 0.77 | 82 |
|  | *Stress* | 0.62 | 0.56 | 0.59 | 0.70 | 0.30 | 0.42 | 54 |
|  | *Accuracy* |  |  | 0.69 |  |  | 0.67 | 136 |
|  | *Weighted Average* | 0.69 | 0.69 | 0.69 | 0.68 | 0.61 | 0.59 | 136 |
|  | *Macro Average* | 0.68 | 0.67 | 0.67 | 0.68 | 0.61 | 0.59 | 136 |
| Gender – Female | *No Stress* | 0.70 | 0.66 | 0.68 | 0.66 | 0.72 | 0.69 | 145 |
|  | *Stress* | 0.66 | 0.70 | 0.68 | 0.67 | 0.60 | 0.63 | 137 |
|  | *Accuracy* |  |  | 0.68 |  |  | 0.66 | 282 |
|  | *Weighted Average* | 0.68 | 0.68 | 0.68 | 0.66 | 0.66 | 0.66 | 282 |
|  | *Macro Average* | 0.68 | 0.68 | 0.68 | 0.66 | 0.66 | 0.66 | 282 |
| Gender – Female (SMOTE) | *No Stress* | 0.70 | 0.62 | 0.66 | 0.66 | 0.70 | 0.68 | 145 |
|  | *Stress* | 0.64 | 0.72 | 0.68 | 0.66 | 0.61 | 0.64 | 137 |
|  | *Accuracy* |  |  | 0.67 |  |  | 0.66 | 282 |
|  | *Weighted Average* | 0.67 | 0.67 | 0.67 | 0.62 | 0.66 | 0.66 | 282 |
|  | *Macro Average* | 0.67 | 0.67 | 0.67 | 0.62 | 0.66 | 0.66 | 282 |
| Employment - Student | *No Stress* | 0.71 | 0.66 | 0.68 | 0.70 | 0.62 | 0.66 | 96 |
|  | *Stress* | 0.64 | 0.69 | 0.66 | 0.62 | 0.69 | 0.65 | 84 |
|  | *Accuracy* |  |  | 0.67 |  |  | 0.66 | 180 |
|  | *Weighted Average* | 0.67 | 0.67 | 0.67 | 0.66 | 0.66 | 0.66 | 180 |
|  | *Macro Average* | 0.67 | 0.67 | 0.67 | 0.66 | 0.66 | 0.66 | 180 |
| Employment – Student (SMOTE) | *No Stress* | 0.72 | 0.66 | 0.69 | 0.64 | 0.77 | 0.70 | 96 |
|  | *Stress* | 0.65 | 0.71 | 0.68 | 0.66 | 0.50 | 0.57 | 84 |
|  | *Accuracy* |  |  | 0.68 |  |  | 0.64 | 180 |
|  | *Weighted Average* | 0.69 | 0.68 | 0.68 | 0.65 | 0.64 | 0.64 | 180 |
|  | *Macro Average* | 0.68 | 0.69 | 0.68 | 0.65 | 0.64 | 0.63 | 180 |
| Employment – Worker | *No Stress* | 0.66 | 0.73 | 0.70 | 0.67 | 0.77 | 0.72 | 122 |
|  | *Stress* | 0.64 | 0.56 | 0.60 | 0.67 | 0.55 | 0.61 | 103 |
|  | *Accuracy* |  |  | 0.65 |  |  | 0.67 | 225 |
|  | *Weighted Average* | 0.65 | 0.65 | 0.65 | 0.67 | 0.66 | 0.66 | 225 |
|  | *Macro Average* | 0.65 | 0.65 | 0.65 | 0.67 | 0.67 | 0.67 | 225 |
| Employment – Worker (SMOTE) | *No Stress* | 0.67 | 0.69 | 0.68 | 0.66 | 0.75 | 0.70 | 122 |
|  | *Stress* | 0.62 | 0.60 | 0.61 | 0.65 | 0.54 | 0.59 | 103 |
|  | *Accuracy* |  |  | 0.65 |  |  | 0.66 | 225 |
|  | *Weighted Average* | 0.65 | 0.65 | 0.65 | 0.66 | 0.65 | 0.65 | 225 |
|  | *Macro Average* | 0.65 | 0.65 | 0.65 | 0.66 | 0.66 | 0.65 | 225 |
| Income - Low | *No Stress* | 0.64 | 0.82 | 0.72 | 0.65 | 0.70 | 0.67 | 131 |
|  | *Stress* | 0.55 | 0.33 | 0.41 | 0.50 | 0.44 | 0.47 | 89 |
|  | *Accuracy* |  |  | 0.62 |  |  | 0.60 | 220 |
|  | *Weighted Average* | 0.60 | 0.62 | 0.59 | 0.59 | 0.60 | 0.59 | 220 |
|  | *Macro Average* | 0.59 | 0.57 | 0.56 | 0.57 | 0.57 | 0.57 | 220 |
| Income – Low (SMOTE) | *No Stress* | 0.66 | 0.63 | 0.64 | 0.64 | 0.86 | 0.74 | 131 |
|  | *Stress* | 0.48 | 0.52 | 0.50 | 0.59 | 0.29 | 0.39 | 89 |
|  | *Accuracy* |  |  | 0.58 |  |  | 0.63 | 220 |
|  | *Weighted Average* | 0.59 | 0.58 | 0.58 | 0.62 | 0.63 | 0.60 | 220 |
|  | *Macro Average* | 0.57 | 0.57 | 0.57 | 0.62 | 0.58 | 0.56 | 220 |
| Income – Medium High | *No Stress* | 0.64 | 0.66 | 0.65 | 0.67 | 0.62 | 0.64 | 107 |
|  | *Stress* | 0.65 | 0.63 | 0.64 | 0.65 | 0.69 | 0.67 | 108 |
|  | *Accuracy* |  |  | 0.65 |  |  | 0.66 | 215 |
|  | *Weighted Average* | 0.65 | 0.65 | 0.65 | 0.66 | 0.66 | 0.66 | 215 |
|  | *Macro Average* | 0.65 | 0.65 | 0.65 | 0.66 | 0.66 | 0.66 | 215 |
| Income – Medium High (SMOTE) | *No Stress* | 0.68 | 0.64 | 0.66 | 0.67 | 0.62 | 0.64 | 107 |
|  | *Stress* | 0.67 | 0.70 | 0.68 | 0.65 | 0.69 | 0.67 | 108 |
|  | *Accuracy* |  |  | 0.67 |  |  | 0.66 | 215 |
|  | *Weighted Average* | 0.67 | 0.67 | 0.67 | 0.66 | 0.66 | 0.66 | 215 |
|  | *Macro Average* | 0.67 | 0.67 | 0.67 | 0.66 | 0.66 | 0.66 | 215 |
| Age – 18-24 | *No Stress* | 0.68 | 0.95 | 0.79 | 0.69 | 0.77 | 0.72 | 69 |
|  | *Stress* | 0.50 | 0.10 | 0.17 | 0.39 | 0.30 | 0.34 | 30 |
|  | *Accuracy* |  |  | 0.67 |  |  | 0.61 | 99 |
|  | *Weighted Average* | 0.62 | 0.67 | 0.58 | 0.59 | 0.61 | 0.60 | 99 |
|  | *Macro Average* | 0.59 | 0.53 | 0.48 | 0.54 | 0.53 | 0.53 | 99 |
| Age – 18-24 (SMOTE) | *No Stress* | 0.69 | 0.67 | 0.68 | 0.66 | 0.80 | 0.72 | 69 |
|  | *Stress* | 0.38 | 0.40 | 0.39 | 0.29 | 0.17 | 0.21 | 30 |
|  | *Accuracy* |  |  | 0.58 |  |  | 0.59 | 99 |
|  | *Weighted Average* | 0.53 | 0.53 | 0.53 | 0.54 | 0.59 | 0.55 | 99 |
|  | *Macro Average* | 0.58 | 0.58 | 0.58 | 0.48 | 0.48 | 0.47 | 99 |
| Age 25-34 | *No Stress* | 0.79 | 0.44 | 0.56 | 0.64 | 0.52 | 0.57 | 71 |
|  | *Stress* | 0.70 | 0.92 | 0.79 | 0.70 | 0.79 | 0.74 | 100 |
|  | *Accuracy* |  |  | 0.72 |  |  | 0.68 | 171 |
|  | *Weighted Average* | 0.74 | 0.72 | 0.70 | 0.67 | 0.68 | 0.67 | 171 |
|  | *Macro Average* | 0.75 | 0.68 | 0.68 | 0.67 | 0.68 | 0.67 | 171 |
| Age 25-34 (SMOTE) | *No Stress* | 0.63 | 0.56 | 0.60 | 0.63 | 0.41 | 0.50 | 71 |
|  | *Stress* | 0.71 | 0.77 | 0.74 | 0.66 | 0.83 | 0.74 | 100 |
|  | *Accuracy* |  |  | 0.68 |  |  | 0.65 | 171 |
|  | *Weighted Average* | 0.68 | 0.68 | 0.68 | 0.65 | 0.65 | 0.64 | 171 |
|  | *Macro Average* | 0.67 | 0.67 | 0.67 | 0.65 | 0.62 | 0.62 | 171 |
| Age 35-44 | *No Stress* | 0.65 | 0.95 | 0.77 | 0.72 | 0.88 | 0.79 | 59 |
|  | *Stress* | 0.70 | 0.19 | 0.30 | 0.71 | 0.46 | 0.56 | 37 |
|  | *Accuracy* |  |  | 0.66 |  |  | 0.72 | 96 |
|  | *Weighted Average* | 0.67 | 0.66 | 0.59 | 0.72 | 0.72 | 0.70 | 96 |
|  | *Macro Average* | 0.68 | 0.57 | 0.54 | 0.72 | 0.67 | 0.68 | 96 |
| Age 35-44 (SMOTE) | *No Stress* | 0.67 | 0.83 | 0.74 | 0.67 | 0.75 | 0.70 | 59 |
|  | *Stress* | 0.57 | 0.35 | 0.43 | 0.50 | 0.41 | 0.45 | 37 |
|  | *Accuracy* |  |  | 0.65 |  |  | 0.61 | 96 |
|  | *Weighted Average* | 0.63 | 0.65 | 0.62 | 0.60 | 0.61 | 0.61 | 96 |
|  | *Macro Average* | 0.62 | 0.59 | 0.59 | 0.58 | 0.61 | 0.61 | 168 |
| Healthy | *No Stress* | 0.65 | 0.81 | 0.72 | 0.66 | 0.76 | 0.71 | 186 |
|  | *Stress* | 0.61 | 0.41 | 0.59 | 0.58 | 0.45 | 0.51 | 135 |
|  | *Accuracy* |  |  | 0.65 |  |  | 0.63 | 321 |
|  | *Weighted Average* | 0.64 | 0.64 | 0.63 | 0.63 | 0.63 | 0.62 | 321 |
|  | *Macro Average* | 0.63 | 0.61 | 0.61 | 0.62 | 0.61 | 0.61 | 321 |
| Healthy (SMOTE) | *No Stress* | 0.69 | 0.70 | 0.69 | 0.66 | 0.66 | 0.66 | 186 |
|  | *Stress* | 0.58 | 0.56 | 0.57 | 0.53 | 0.53 | 0.53 | 135 |
|  | *Accuracy* |  |  | 0.64 |  |  | 0.60 | 321 |
|  | *Weighted Average* | 0.64 | 0.64 | 0.64 | 0.60 | 0.60 | 0.60 | 321 |
|  | *Macro Average* | 0.63 | 0.63 | 0.63 | 0.59 | 0.60 | 0.60 | 321 |

Table B5: Precision, Recall, F1-Score, Accuracy for Sleep Datasets, Generalized

| **SDA** | **RF** | | | | | **SVM** | | | **Support** |
| --- | --- | --- | --- | --- | --- | --- | --- | --- | --- |
|  | **Items** | ***Precision*** | ***Recall*** | | ***F1-Score*** | ***Precision*** | ***Recall*** | ***F1-Score*** |  |
| Complete Dataset | *No Stress* | 0.72 | 0.78 | | 0.75 | 0.71 | 0.65 | 0.68 | 309 |
|  | *Stress* | 0.68 | 0.61 | | 0.65 | 0.59 | 0.65 | 0.62 | 238 |
|  | *Accuracy* | - | - | | 0.71 | - | - | 0.65 | 547 |
|  | *Weighted Average* | 0.71 | 0.71 | | 0.71 | 0.65 | 0.65 | 0.65 | 547 |
|  | *Macro Average* | 0.70 | 0.70 | | 0.70 | 0.65 | 0.65 | 0.65 | 547 |
| Complete Dataset (SMOTE) | *No Stress* | 0.77 | 0.76 | | 0.76 | 0.68 | 0.82 | 0.75 | 309 |
|  | *Stress* | 0.69 | 0.71 | | 0.70 | 0.69 | 0.50 | 0.58 | 238 |
|  | *Accuracy* | - | - | | 0.73 | - | - | 0.68 | 547 |
|  | *Weighted Average* | 0.74 | 0.73 | | 0.74 | 0.68 | 0.68 | 0.67 | 547 |
|  | *Macro Average* | 0.73 | 0.73 | | 0.73 | 0.68 | 0.66 | 0.66 | 547 |
| Gender - Male | *No Stress* | 0.72 | 0.85 | | 0.78 | 0.72 | 0.92 | 0.81 | 102 |
|  | *Stress* | 0.58 | 0.39 | | 0.47 | 0.69 | 0.33 | 0.45 | 54 |
|  | *Accuracy* |  |  | | 0.69 |  |  | 0.72 | 156 |
|  | *Weighted Average* | 0.68 | 0.69 | | 0.67 | 0.71 | 0.72 | 0.69 | 156 |
|  | *Macro Average* | 0.65 | 0.62 | | 0.63 | 0.71 | 0.63 | 0.63 | 156 |
| Gender – Male (SMOTE) | *No Stress* | 0.75 | 0.80 | | 0.77 | 0.73 | 0.93 | 0.82 | 102 |
|  | *Stress* | 0.57 | 0.48 | | 0.52 | 0.72 | 0.33 | 0.46 | 54 |
|  | *Accuracy* |  |  | | 0.69 |  |  | 0.72 | 156 |
|  | *Weighted Average* | 0.68 | 0.69 | | 0.69 | 0.72 | 0.72 | 0.69 | 156 |
|  | *Macro Average* | 0.66 | 0.64 | | 0.65 | 0.72 | 0.63 | 0.64 | 156 |
| Gender – Female | *No Stress* | 0.72 | 0.70 | | 0.71 | 0.68 | 0.64 | 0.66 | 186 |
|  | *Stress* | 0.69 | 0.71 | | 0.70 | 0.64 | 0.68 | 0.66 | 173 |
|  | *Accuracy* |  |  | | 0.71 |  |  | 0.66 | 359 |
|  | *Weighted Average* | 0.71 | 0.71 | | 0.71 | 0.66 | 0.66 | 0.66 | 359 |
|  | *Macro Average* | 0.71 | 0.71 | | 0.71 | 0.66 | 0.66 | 0.66 | 359 |
| Gender – Female (SMOTE) | *No Stress* | 0.73 | 0.70 | | 0.71 | 0.64 | 0.75 | 0.69 | 186 |
|  | *Stress* | 0.69 | 0.72 | | 0.70 | 0.67 | 0.54 | 0.60 | 173 |
|  | *Accuracy* |  |  | | 0.71 |  |  | 0.65 | 359 |
|  | *Weighted Average* | 0.71 | 0.71 | | 0.71 | 0.65 | 0.65 | 0.64 | 359 |
|  | *Macro Average* | 0.71 | 0.71 | | 0.71 | 0.65 | 0.65 | 0.64 | 359 |
| Employment - Student | *No Stress* | 0.73 | 0.65 | | 0.69 | 0.75 | 0.58 | 0.65 | 135 |
|  | *Stress* | 0.65 | 0.73 | | 0.69 | 0.62 | 0.79 | 0.70 | 121 |
|  | *Accuracy* |  |  | | 0.69 |  |  | 0.68 | 256 |
|  | *Weighted Average* | 0.69 | 0.69 | | 0.69 | 0.69 | 0.68 | 0.67 | 256 |
|  | *Macro Average* | 0.69 | 0.69 | | 0.69 | 0.69 | 0.68 | 0.67 | 256 |
| Employment – Student (SMOTE) | *No Stress* | 0.74 | 0.64 | | 0.69 | 0.70 | 0.66 | 0.68 | 135 |
|  | *Stress* | 0.65 | 0.75 | | 0.70 | 0.64 | 0.69 | 0.66 | 121 |
|  | *Accuracy* |  |  | | 0.69 |  |  | 0.67 | 256 |
|  | *Weighted Average* | 0.70 | 0.69 | | 0.69 | 0.67 | 0.67 | 0.67 | 256 |
|  | *Macro Average* | 0.70 | 0.69 | | 0.69 | 0.67 | 0.67 | 0.67 | 256 |
| Employment – Worker | *No Stress* | 0.71 | 0.84 | | 0.77 | 0.71 | 0.78 | 0.75 | 169 |
|  | *Stress* | 0.65 | 0.47 | | 0.54 | 0.59 | 0.50 | 0.55 | 107 |
|  | *Accuracy* |  |  | | 0.70 |  |  | 0.67 | 276 |
|  | *Weighted Average* | 0.69 | 0.70 | | 0.68 | 0.67 | 0.67 | 0.67 | 276 |
|  | *Macro Average* | 0.68 | 0.65 | | 0.66 | 0.65 | 0.64 | 0.65 | 276 |
| Employment – Worker (SMOTE) | *No Stress* | 0.74 | 0.77 | | 0.76 | 0.68 | 0.86 | 0.76 | 169 |
|  | *Stress* | 0.61 | 0.58 | | 0.60 | 0.62 | 0.37 | 0.47 | 107 |
|  | *Accuracy* |  |  | | 0.70 |  |  | 0.67 | 276 |
|  | *Weighted Average* | 0.69 | 0.70 | | 0.69 | 0.66 | 0.67 | 0.65 | 276 |
|  | *Macro Average* | 0.68 | 0.67 | | 0.68 | 0.65 | 0.62 | 0.61 | 276 |
| Income - Low | *No Stress* | 0.74 | 0.81 | | 0.78 | 0.68 | 0.70 | 0.69 | 161 |
|  | *Stress* | 0.67 | 0.57 | | 0.62 | 0.53 | 0.52 | 0.53 | 108 |
|  | *Accuracy* |  |  | | 0.72 |  |  | 0.62 | 269 |
|  | *Weighted Average* | 0.71 | 0.72 | | 0.71 | 0.62 | 0.62 | 0.62 | 269 |
|  | *Macro Average* | 0.71 | 0.69 | | 0.70 | 0.61 | 0.61 | 0.61 | 269 |
| Income – Low (SMOTE) | *No Stress* | 0.77 | 0.76 | | 0.77 | 0.70 | 0.90 | 0.79 | 161 |
|  | *Stress* | 0.65 | 0.67 | | 0.66 | 0.74 | 0.43 | 0.54 | 108 |
|  | *Accuracy* |  |  | | 0.72 |  |  | 0.71 | 269 |
|  | *Weighted Average* | 0.73 | 0.72 | | 0.73 | 0.72 | 0.71 | 0.69 | 269 |
|  | *Macro Average* | 0.71 | 0.72 | | 0.71 | 0.72 | 0.66 | 0.66 | 269 |
| Income – Medium High | *No Stress* | 0.76 | 0.75 | | 0.76 | 0.73 | 0.75 | 0.74 | 122 |
|  | *Stress* | 0.73 | 0.74 | | 0.74 | 0.72 | 0.70 | 0.71 | 111 |
|  | *Accuracy* |  |  | | 0.75 |  |  | 0.73 | 233 |
|  | *Weighted Average* | 0.75 | 0.75 | | 0.75 | 0.73 | 0.73 | 0.73 | 233 |
|  | *Macro Average* | 0.75 | 0.75 | | 0.75 | 0.72 | 0.72 | 0.72 | 233 |
| Income – Medium High (SMOTE) | *No Stress* | 0.76 | 0.69 | | 0.72 | 0.69 | 0.68 | 0.68 | 122 |
|  | *Stress* | 0.69 | 0.76 | | 0.72 | 0.65 | 0.66 | 0.65 | 111 |
|  | *Accuracy* |  |  | | 0.72 |  |  | 0.67 | 233 |
|  | *Weighted Average* | 0.72 | 0.72 | | 0.72 | 0.67 | 0.67 | 0.67 | 233 |
|  | *Macro Average* | 0.72 | 0.72 | | 0.72 | 0.67 | 0.67 | 0.67 | 233 |
| Age – 18-24 | *No Stress* | 0.74 | 0.83 | | 0.78 | 0.73 | 0.65 | 0.69 | 103 |
|  | *Stress* | 0.68 | 0.57 | | 0.62 | 0.55 | 0.64 | 0.59 | 69 |
|  | *Accuracy* |  |  | | 0.72 |  |  | 0.65 | 172 |
|  | *Weighted Average* | 0.72 | 0.70 | | 0.70 | 0.64 | 0.64 | 0.64 | 172 |
|  | *Macro Average* | 0.71 | 0.70 | | 0.70 | 0.66 | 0.65 | 0.65 | 172 |
| Age – 18-24 (SMOTE) | *No Stress* | 0.77 | 0.75 | | 0.76 | 0.69 | 0.83 | 0.75 | 103 |
|  | *Stress* | 0.64 | 0.67 | | 0.65 | 0.64 | 0.43 | 0.52 | 69 |
|  | *Accuracy* |  |  | | 0.72 |  |  | 0.67 | 172 |
|  | *Weighted Average* | 0.72 | 0.72 | | 0.72 | 0.67 | 0.67 | 0.66 | 172 |
|  | *Macro Average* | 0.70 | 0.71 | | 0.71 | 0.66 | 0.63 | 0.64 | 172 |
| Age 25-34 | *No Stress* | 0.65 | 0.53 | | 0.59 | 0.58 | 0.41 | 0.48 | 68 |
|  | *Stress* | 0.73 | 0.82 | | 0.77 | 0.68 | 0.81 | 0.74 | 105 |
|  | *Accuracy* |  |  | | 0.71 |  |  | 0.65 | 173 |
|  | *Weighted Average* | 0.70 | 0.71 | | 0.70 | 0.64 | 0.65 | 0.64 | 173 |
|  | *Macro Average* | 0.69 | 0.67 | | 0.68 | 0.63 | 0.61 | 0.61 | 173 |
| Age 25-34 (SMOTE) | *No Stress* | 0.59 | 0.65 | | 0.62 | 0.67 | 0.41 | 0.51 | 68 |
|  | *Stress* | 0.76 | 0.71 | | 0.74 | 0.69 | 0.87 | 0.77 | 105 |
|  | *Accuracy* |  |  | | 0.69 |  |  | 0.69 | 173 |
|  | *Weighted Average* | 0.68 | 0.68 | | 0.68 | 0.68 | 0.69 | 0.67 | 173 |
|  | *Macro Average* | 0.69 | 0.69 | | 0.69 | 0.68 | 0.64 | 0.64 | 173 |
| Age 35-44 | *No Stress* | 0.78 | 0.95 | | 0.85 | 0.79 | 0.84 | 0.81 | 98 |
|  | *Stress* | 0.76 | 0.37 | | 0.50 | 0.57 | 0.49 | 0.53 | 43 |
|  | *Accuracy* |  |  | | 0.77 |  |  | 0.73 | 141 |
|  | *Weighted Average* | 0.77 | 0.77 | | 0.75 | 0.72 | 0.73 | 0.72 | 141 |
|  | *Macro Average* | 0.77 | 0.66 | | 0.68 | 0.68 | 0.66 | 0.67 | 141 |
| Age 35-44 (SMOTE) | *No Stress* | 0.80 | 0.82 | | 0.81 | 0.75 | 0.91 | 0.82 | 98 |
|  | *Stress* | 0.56 | 0.53 | | 0.55 | 0.59 | 0.30 | 0.40 | 43 |
|  | *Accuracy* |  |  | | 0.73 |  |  | 0.72 | 141 |
|  | *Weighted Average* | 0.73 | 0.73 | | 0.73 | 0.70 | 0.72 | 0.69 | 141 |
|  | *Macro Average* | 0.68 | 0.68 | | 0.68 | 0.67 | 0.61 | 0.61 | 141 |
| Healthy | *No Stress* | 0.75 | 0.83 | | 0.79 | 0.71 | 0.74 | 0.73 | 254 |
|  | *Stress* | 0.70 | 0.60 | | 0.65 | 0.60 | 0.56 | 0.58 | 174 |
|  | *Accuracy* |  |  | | 0.73 |  |  | 0.67 | 428 |
|  | *Weighted Average* | 0.73 | 0.73 | | 0.73 | 0.66 | 0.65 | 0.65 | 428 |
|  | *Macro Average* | 0.73 | 0.71 | | 0.72 | 0.67 | 0.67 | 0.67 | 428 |
| Healthy - SMOTE | *No Stress* | 0.77 | 0.77 | | 0.77 | 0.71 | 0.70 | 0.70 | 254 |
|  | *Stress* | 0.66 | 0.66 | | 0.66 | 0.57 | 0.58 | 0.58 | 174 |
|  | *Accuracy* |  |  | | 0.72 |  |  | 0.65 | 428 |
|  | *Weighted Average* | 0.72 | 0.72 | | 0.72 | 0.65 | 0.65 | 0.65 | 428 |
|  | *Macro Average* | 0.71 | 0.71 | | 0.71 | 0.64 | 0.64 | 0.64 | 428 |
| **SDAW** | **RF** | | | | | **SVM** | | |  |
|  | **Items** | ***Precision*** | ***Recall*** | | ***F1-Score*** | ***Precision*** | ***Recall*** | ***F1-Score*** | ***Support*** |
| Complete Dataset | *No Stress* | 0.75 | 0.82 | | 0.79 | 0.72 | 0.80 | 0.76 | 249 |
|  | *Stress* | 0.72 | 0.63 | | 0.67 | 0.67 | 0.58 | 0.62 | 179 |
|  | *Accuracy* | - | - | | 0.74 | - | - | 0.70 | 428 |
|  | *Weighted Average* | 0.74 | 0.74 | | 0.74 | 0.70 | 0.70 | 0.70 | 428 |
|  | *Macro Average* | 0.74 | 0.72 | | 0.73 | 0.70 | 0.69 | 0.69 | 428 |
| Complete Dataset (SMOTE) | *No Stress* | 0.78 | 0.75 | | 0.76 | 0.76 | 0.76 | 0.76 | 249 |
|  | *Stress* | 0.67 | 0.70 | | 0.68 | 0.66 | 0.66 | 0.66 | 179 |
|  | *Accuracy* | - | - | | 0.73 | - | - | 0.72 | 428 |
|  | *Weighted Average* | 0.73 | 0.73 | | 0.73 | 0.72 | 0.72 | 0.72 | 428 |
|  | *Macro Average* | 0.72 | 0.72 | | 0.72 | 0.71 | 0.71 | 0.71 | 428 |
| Gender - Male | *No Stress* | 0.83 | 0.93 | | 0.88 | 0.83 | 0.88 | 0.85 | 73 |
|  | *Stress* | 0.29 | 0.12 | | 0.17 | 0.25 | 0.19 | 0.21 | 16 |
|  | *Accuracy* |  |  | | 0.79 |  |  | 0.75 | 89 |
|  | *Weighted Average* | 0.73 | 0.79 | | 0.75 | 0.73 | 0.75 | 0.74 | 89 |
|  | *Macro Average* | 0.56 | 0.53 | | 0.53 | 0.54 | 0.53 | 0.53 | 89 |
| Gender – Male (SMOTE) | *No Stress* | 0.83 | 0.88 | | 0.85 | 0.82 | 0.99 | 0.89 | 73 |
|  | *Stress* | 0.25 | 0.19 | | 0.21 | 0.00 | 0.00 | 0.00 | 16 |
|  | *Accuracy* |  |  | | 0.75 |  |  | 0.81 | 89 |
|  | *Weighted Average* | 0.73 | 0.75 | | 0.74 | 0.67 | 0.81 | 0.73 | 89 |
|  | *Macro Average* | 0.54 | 0.53 | | 0.53 | 0.41 | 0.49 | 0.45 | 89 |
| Gender – Female | *No Stress* | 0.70 | 0.73 | | 0.72 | 0.68 | 0.67 | 0.68 | 166 |
|  | *Stress* | 0.70 | 0.68 | | 0.69 | 0.66 | 0.66 | 0.66 | 157 |
|  | *Accuracy* |  |  | | 0.70 |  |  | 0.67 | 323 |
|  | *Weighted Average* | 0.70 | 0.70 | | 0.70 | 0.67 | 0.67 | 0.67 | 323 |
|  | *Macro Average* | 0.70 | 0.70 | | 0.70 | 0.67 | 0.67 | 0.67 | 323 |
| Gender – Female (SMOTE) | *No Stress* | 0.71 | 0.72 | | 0.72 | 0.68 | 0.63 | 0.65 | 166 |
|  | *Stress* | 0.70 | 0.69 | | 0.70 | 0.64 | 0.69 | 0.66 | 157 |
|  | *Accuracy* |  |  | | 0.71 |  |  | 0.66 | 323 |
|  | *Weighted Average* | 0.71 | 0.71 | | 0.71 | 0.66 | 0.66 | 0.66 | 323 |
|  | *Macro Average* | 0.71 | 0.71 | | 0.71 | 0.66 | 0.66 | 0.66 | 323 |
| Employment - Student | *No Stress* | 0.75 | 0.75 | | 0.75 | 0.74 | 0.66 | 0.70 | 99 |
|  | *Stress* | 0.73 | 0.73 | | 0.73 | 0.67 | 0.75 | 0.71 | 92 |
|  | *Accuracy* |  |  | | 0.74 |  |  | 0.70 | 191 |
|  | *Weighted Average* | 0.74 | 0.74 | | 0.74 | 0.71 | 0.70 | 0.70 | 191 |
|  | *Macro Average* | 0.74 | 0.74 | | 0.74 | 0.70 | 0.70 | 0.70 | 191 |
| Employment – Student (SMOTE) | *No Stress* | 0.76 | 0.73 | | 0.74 | 0.75 | 0.74 | 0.74 | 99 |
|  | *Stress* | 0.72 | 0.75 | | 0.73 | 0.72 | 0.74 | 0.73 | 92 |
|  | *Accuracy* |  |  | | 0.74 |  |  | 0.74 | 191 |
|  | *Weighted Average* | 0.74 | 0.74 | | 0.74 | 0.74 | 0.74 | 0.74 | 191 |
|  | *Macro Average* | 0.74 | 0.74 | | 0.74 | 0.74 | 0.74 | 0.74 | 191 |
| Employment – Worker | *No Stress* | 0.74 | 0.84 | | 0.79 | 0.74 | 0.78 | 0.76 | 151 |
|  | *Stress* | 0.64 | 0.49 | | 0.56 | 0.58 | 0.52 | 0.55 | 87 |
|  | *Accuracy* |  |  | | 0.71 |  |  | 0.68 | 238 |
|  | *Weighted Average* | 0.71 | 0.71 | | 0.70 | 0.68 | 0.68 | 0.68 | 238 |
|  | *Macro Average* | 0.69 | 0.67 | | 0.67 | 0.66 | 0.65 | 0.65 | 238 |
| Employment – Worker (SMOTE) | *No Stress* | 0.77 | 0.81 | | 0.79 | 0.73 | 0.73 | 0.73 | 151 |
|  | *Stress* | 0.64 | 0.59 | | 0.61 | 0.53 | 0.53 | 0.53 | 87 |
|  | *Accuracy* |  |  | | 0.73 |  |  | 0.66 | 238 |
|  | *Weighted Average* | 0.72 | 0.73 | | 0.72 | 0.66 | 0.66 | 0.66 | 238 |
|  | *Macro Average* | 0.70 | 0.70 | | 0.70 | 0.63 | 0.63 | 0.63 | 238 |
| Income - Low | *No Stress* | 0.80 | 0.82 | | 0.81 | 0.78 | 0.76 | 0.77 | 119 |
|  | *Stress* | 0.69 | 0.67 | | 0.68 | 0.62 | 0.64 | 0.63 | 73 |
|  | *Accuracy* |  |  | | 0.76 |  |  | 0.71 | 192 |
|  | *Weighted Average* | 0.76 | 0.76 | | 0.76 | 0.72 | 0.71 | 0.71 | 192 |
|  | *Macro Average* | 0.75 | 0.74 | | 0.74 | 0.70 | 0.70 | 0.70 | 192 |
| Income – Low (SMOTE) | *No Stress* | 0.83 | 0.75 | | 0.79 | 0.80 | 0.73 | 0.76 | 119 |
|  | *Stress* | 0.65 | 0.75 | | 0.70 | 0.61 | 0.70 | 0.65 | 73 |
|  | *Accuracy* |  |  | | 0.75 |  |  | 0.72 | 192 |
|  | *Weighted Average* | 0.76 | 0.75 | | 0.75 | 0.73 | 0.72 | 0.72 | 192 |
|  | *Macro Average* | 0.74 | 0.75 | | 0.74 | 0.71 | 0.71 | 0.71 | 192 |
| Income – Medium High | *No Stress* | 0.71 | 0.75 | | 0.73 | 0.72 | 0.75 | 0.73 | 110 |
|  | *Stress* | 0.64 | 0.77 | | 0.70 | 0.66 | 0.62 | 0.64 | 86 |
|  | *Accuracy* |  |  | | 0.68 |  |  | 0.69 | 196 |
|  | *Weighted Average* | 0.69 | 0.68 | | 0.67 | 0.69 | 0.69 | 0.69 | 196 |
|  | *Macro Average* | 0.68 | 0.68 | | 0.67 | 0.69 | 0.69 | 0.69 | 196 |
| Income – Medium High (SMOTE) | *No Stress* | 0.70 | 0.73 | | 0.71 | 0.71 | 0.73 | 0.72 | 110 |
|  | *Stress* | 0.63 | 0.60 | | 0.62 | 0.64 | 0.63 | 0.64 | 86 |
|  | *Accuracy* |  |  | | 0.67 |  |  | 0.68 | 196 |
|  | *Weighted Average* | 0.67 | 0.67 | | 0.67 | 0.68 | 0.68 | 0.68 | 196 |
|  | *Macro Average* | 0.67 | 0.67 | | 0.67 | 0.68 | 0.68 | 0.68 | 196 |
| Age – 18-24 | *No Stress* | 0.79 | 0.79 | | 0.79 | 0.80 | 0.73 | 0.76 | 66 |
|  | *Stress* | 0.72 | 0.72 | | 0.72 | 0.68 | 0.76 | 0.72 | 50 |
|  | *Accuracy* |  |  | | 0.76 |  |  | 0.74 | 116 |
|  | *Weighted Average* | 0.76 | 0.76 | | 0.76 | 0.75 | 0.74 | 0.74 | 116 |
|  | *Macro Average* | 0.75 | 0.75 | | 0.75 | 0.74 | 0.74 | 0.74 | 116 |
| Age – 18-24 (SMOTE) | *No Stress* | 0.78 | 0.74 | | 0.76 | 0.79 | 0.76 | 0.78 | 66 |
|  | *Stress* | 0.68 | 0.72 | | 0.70 | 0.70 | 0.74 | 0.72 | 50 |
|  | *Accuracy* |  |  | | 0.73 |  |  | 0.75 | 116 |
|  | *Weighted Average* | 0.74 | 0.73 | | 0.73 | 0.75 | 0.75 | 0.75 | 116 |
|  | *Macro Average* | 0.73 | 0.73 | | 0.73 | 0.75 | 0.75 | 0.75 | 116 |
| Age 25-34 | *No Stress* | 0.70 | 0.63 | | 0.66 | 0.64 | 0.57 | 0.60 | 67 |
|  | *Stress* | 0.71 | 0.78 | | 0.74 | 0.67 | 0.74 | 0.70 | 80 |
|  | *Accuracy* |  |  | | 0.71 |  |  | 0.66 | 147 |
|  | *Weighted Average* | 0.71 | 0.71 | | 0.71 | 0.66 | 0.66 | 0.66 | 147 |
|  | *Macro Average* | 0.71 | 0.70 | | 0.71 | 0.66 | 0.65 | 0.65 | 147 |
| Age 25-34 (SMOTE) | *No Stress* | 0.68 | 0.63 | | 0.65 | 0.64 | 0.67 | 0.66 | 67 |
|  | *Stress* | 0.71 | 0.75 | | 0.73 | 0.71 | 0.69 | 0.70 | 80 |
|  | *Accuracy* |  |  | | 0.69 |  |  | 0.68 | 147 |
|  | *Weighted Average* | 0.69 | 0.69 | | 0.69 | 0.68 | 0.68 | 0.68 | 147 |
|  | *Macro Average* | 0.69 | 0.69 | | 0.69 | 0.68 | 0.68 | 0.68 | 147 |
| Age 35-44 | *No Stress* | 0.81 | 0.95 | | 0.88 | 0.84 | 0.89 | 0.86 | 82 |
|  | *Stress* | 0.64 | 0.28 | | 0.39 | 0.55 | 0.44 | 0.49 | 25 |
|  | *Accuracy* |  |  | | 0.79 |  |  | 0.79 | 107 |
|  | *Weighted Average* | 0.77 | 0.79 | | 0.76 | 0.77 | 0.79 | 0.78 | 107 |
|  | *Macro Average* | 0.72 | 0.62 | | 0.63 | 0.69 | 0.67 | 0.68 | 107 |
| Age 35-44 (SMOTE) | *No Stress* | 0.83 | 0.88 | | 0.85 | 0.78 | 0.98 | 0.87 | 82 |
|  | *Stress* | 0.50 | 0.40 | | 0.44 | 0.60 | 0.12 | 0.20 | 25 |
|  | *Accuracy* |  |  | | 0.77 |  |  | 0.78 | 107 |
|  | *Weighted Average* | 0.75 | 0.77 | | 0.76 | 0.74 | 0.78 | 0.71 | 107 |
|  | *Macro Average* | 0.66 | 0.64 | | 0.65 | 0.69 | 0.55 | 0.53 | 107 |
| Healthy | *No Stress* | 0.75 | 0.85 | | 0.80 | 0.76 | 0.75 | 0.75 | 195 |
|  | *Stress* | 0.67 | 0.52 | | 0.58 | 0.58 | 0.60 | 0.59 | 114 |
|  | *Accuracy* |  |  | | 0.73 |  |  | 0.69 | 309 |
|  | *Weighted Average* | 0.72 | 0.73 | | 0.72 | 0.69 | 0.69 | 0.69 | 309 |
|  | *Macro Average* | 0.71 | 0.68 | | 0.69 | 0.67 | 0.67 | 0.67 | 309 |
| Healthy (SMOTE) | *No Stress* | 0.79 | 0.74 | | 0.77 | 0.79 | 0.76 | 0.77 | 195 |
|  | *Stress* | 0.60 | 0.66 | | 0.63 | 0.61 | 0.65 | 0.63 | 114 |
|  | *Accuracy* |  |  | | 0.71 |  |  | 0.72 | 309 |
|  | *Weighted Average* | 0.72 | 0.71 | | 0.71 | 0.72 | 0.72 | 0.72 | 309 |
|  | *Macro Average* | 0.69 | 0.70 | | 0.70 | 0.70 | 0.70 | 0.70 | 309 |
| **SDW** | **RF** | | | | | **SVM** | | |  |
|  | **Items** | ***Precision*** | ***Recall*** | | ***F1-Score*** | ***Precision*** | ***Recall*** | ***F1-Score*** |  |
| Complete Dataset | *No Stress* | 0.73 | 0.76 | | 0.74 | 0.62 | 0.73 | 0.67 | 307 |
|  | *Stress* | 0.65 | 0.62 | | 0.64 | 0.56 | 0.44 | 0.49 | 228 |
|  | *Accuracy* | - | - | | 0.70 | - | - | 0.60 | 535 |
|  | *Weighted Average* | 0.70 | 0.70 | | 0.70 | 0.60 | 0.60 | 0.59 | 535 |
|  | *Macro Average* | 0.69 | 0.69 | | 0.69 | 0.59 | 0.58 | 0.58 | 535 |
| Complete Dataset (SMOTE) | *No Stress* | 0.72 | 0.74 | | 0.73 | 0.73 | 0.74 | 0.73 | 307 |
|  | *Stress* | 0.64 | 0.61 | | 0.62 | 0.64 | 0.63 | 0.63 | 228 |
|  | *Accuracy* | - | - | | 0.68 | - | - | 0.69 | 535 |
|  | *Weighted Average* | 0.68 | 0.68 | | 0.68 | 0.69 | 0.69 | 0.69 | 535 |
|  | *Macro Average* | 0.68 | 0.67 | | 0.67 | 0.68 | 0.68 | 0.68 | 535 |
| Gender - Male | *No Stress* | 0.80 | 0.96 | | 0.88 | 0.83 | 0.93 | 0.88 | 85 |
|  | *Stress* | 0.90 | 0.58 | | 0.71 | 0.84 | 0.67 | 0.74 | 48 |
|  | *Accuracy* |  |  | | 0.83 |  |  | 0.83 | 133 |
|  | *Weighted Average* | 0.84 | 0.83 | | 0.82 | 0.84 | 0.83 | 0.83 | 133 |
|  | *Macro Average* | 0.85 | 0.77 | | 0.79 | 0.84 | 0.80 | 0.81 | 133 |
| Gender – Male (SMOTE) | *No Stress* | 0.83 | 0.91 | | 0.87 | 0.86 | 0.86 | 0.86 | 85 |
|  | *Stress* | 0.80 | 0.67 | | 0.73 | 0.75 | 0.75 | 0.75 | 48 |
|  | *Accuracy* |  |  | | 0.82 |  |  | 0.82 | 133 |
|  | *Weighted Average* | 0.82 | 0.82 | | 0.82 | 0.82 | 0.82 | 0.82 | 133 |
|  | *Macro Average* | 0.81 | 0.79 | | 0.80 | 0.80 | 0.80 | 0.80 | 133 |
| Gender – Female | *No Stress* | 0.70 | 0.75 | | 0.73 | 0.68 | 0.73 | 0.70 | 212 |
|  | *Stress* | 0.67 | 0.61 | | 0.64 | 0.64 | 0.59 | 0.61 | 174 |
|  | *Accuracy* |  |  | | 0.69 |  |  | 0.67 | 386 |
|  | *Weighted Average* | 0.69 | 0.69 | | 0.69 | 0.66 | 0.67 | 0.66 | 386 |
|  | *Macro Average* | 0.69 | 0.68 | | 0.68 | 0.66 | 0.66 | 0.66 | 386 |
| Gender – Female (SMOTE) | *No Stress* | 0.70 | 0.71 | | 0.71 | 0.70 | 0.64 | 0.67 | 212 |
|  | *Stress* | 0.64 | 0.64 | | 0.64 | 0.60 | 0.66 | 0.63 | 174 |
|  | *Accuracy* |  |  | | 0.68 |  |  | 0.65 | 386 |
|  | *Weighted Average* | 0.68 | 0.68 | | 0.68 | 0.65 | 0.65 | 0.65 | 386 |
|  | *Macro Average* | 0.67 | 0.67 | | 0.67 | 0.65 | 0.65 | 0.65 | 386 |
| Employment - Student | *No Stress* | 0.74 | 0.68 | | 0.71 | 0.74 | 0.75 | 0.74 | 99 |
|  | *Stress* | 0.68 | 0.74 | | 0.71 | 0.73 | 0.72 | 0.72 | 92 |
|  | *Accuracy* |  |  | | 0.71 |  |  | 0.73 | 191 |
|  | *Weighted Average* | 0.71 | 0.71 | | 0.71 | 0.73 | 0.73 | 0.73 | 191 |
|  | *Macro Average* | 0.71 | 0.71 | | 0.71 | 0.73 | 0.73 | 0.73 | 191 |
| Employment – Student (SMOTE) | *No Stress* | 0.73 | 0.67 | | 0.69 | 0.75 | 0.73 | 0.74 | 99 |
|  | *Stress* | 0.67 | 0.73 | | 0.70 | 0.72 | 0.74 | 0.73 | 92 |
|  | *Accuracy* |  |  | | 0.70 |  |  | 0.73 | 191 |
|  | *Weighted Average* | 0.70 | 0.70 | | 0.73 | 0.73 | 0.73 | 0.73 | 191 |
|  | *Macro Average* | 0.70 | 0.70 | | 0.73 | 0.73 | 0.73 | 0.73 | 191 |
| Employment – Worker | *No Stress* | 0.72 | 0.76 | | 0.74 | 0.71 | 0.74 | 0.72 | 195 |
|  | *Stress* | 0.62 | 0.57 | | 0.60 | 0.59 | 0.55 | 0.57 | 133 |
|  | *Accuracy* |  |  | | 0.69 |  |  | 0.66 | 328 |
|  | *Weighted Average* | 0.68 | 0.69 | | 0.68 | 0.66 | 0.66 | 0.66 | 328 |
|  | *Macro Average* | 0.67 | 0.67 | | 0.67 | 0.65 | 0.65 | 0.65 | 328 |
| Employment – Worker (SMOTE) | *No Stress* | 0.74 | 0.74 | | 0.74 | 0.71 | 0.72 | 0.72 | 195 |
|  | *Stress* | 0.62 | 0.61 | | 0.61 | 0.58 | 0.56 | 0.57 | 133 |
|  | *Accuracy* |  |  | | 0.69 |  |  | 0.66 | 328 |
|  | *Weighted Average* | 0.69 | 0.69 | | 0.69 | 0.66 | 0.66 | 0.66 | 328 |
|  | *Macro Average* | 0.68 | 0.68 | | 0.68 | 0.64 | 0.64 | 0.64 | 328 |
| Income - Low | *No Stress* | 0.73 | 0.85 | | 0.78 | 0.74 | 0.82 | 0.78 | 144 |
|  | *Stress* | 0.66 | 0.49 | | 0.56 | 0.64 | 0.53 | 0.58 | 88 |
|  | *Accuracy* |  |  | | 0.71 |  |  | 0.71 | 232 |
|  | *Weighted Average* | 0.70 | 0.71 | | 0.70 | 0.70 | 0.71 | 0.70 | 232 |
|  | *Macro Average* | 0.70 | 0.67 | | 0.67 | 0.69 | 0.68 | 0.68 | 232 |
| Income – Low (SMOTE) | *No Stress* | 0.76 | 0.78 | | 0.77 | 0.76 | 0.76 | 0.76 | 144 |
|  | *Stress* | 0.62 | 0.60 | | 0.61 | 0.61 | 0.61 | 0.61 | 88 |
|  | *Accuracy* |  |  | | 0.71 |  |  | 0.70 | 232 |
|  | *Weighted Average* | 0.71 | 0.71 | | 0.71 | 0.70 | 0.70 | 0.70 | 232 |
|  | *Macro Average* | 0.69 | 0.69 | | 0.69 | 0.68 | 0.68 | 0.68 | 232 |
| Income – Medium High | *No Stress* | 0.78 | 0.65 | | 0.71 | 0.79 | 0.69 | 0.74 | 149 |
|  | *Stress* | 0.65 | 0.78 | | 0.71 | 0.68 | 0.78 | 0.72 | 125 |
|  | *Accuracy* |  |  | | 0.71 |  |  | 0.73 | 274 |
|  | *Weighted Average* | 0.72 | 0.71 | | 0.71 | 0.74 | 0.73 | 0.73 | 274 |
|  | *Macro Average* | 0.72 | 0.72 | | 0.71 | 0.73 | 0.73 | 0.73 | 274 |
| Income – Medium High (SMOTE) | *No Stress* | 0.79 | 0.64 | | 0.71 | 0.80 | 0.66 | 0.73 | 149 |
|  | *Stress* | 0.65 | 0.79 | | 0.71 | 0.67 | 0.80 | 0.73 | 125 |
|  | *Accuracy* |  |  | | 0.71 |  |  | 0.73 | 274 |
|  | *Weighted Average* | 0.73 | 0.71 | | 0.71 | 0.74 | 0.73 | 0.73 | 274 |
|  | *Macro Average* | 0.72 | 0.72 | | 0.71 | 0.73 | 0.73 | 0.73 | 274 |
| Age – 18-24 | *No Stress* | 0.75 | 0.66 | | 0.70 | 0.82 | 0.66 | 0.73 | 80 |
|  | *Stress* | 0.60 | 0.69 | | 0.65 | 0.64 | 0.80 | 0.71 | 59 |
|  | *Accuracy* |  |  | | 0.68 |  |  | 0.72 | 139 |
|  | *Weighted Average* | 0.69 | 0.68 | | 0.68 | 0.74 | 0.72 | 0.72 | 139 |
|  | *Macro Average* | 0.67 | 0.68 | | 0.67 | 0.73 | 0.73 | 0.72 | 139 |
| Age – 18-24 (SMOTE) | *No Stress* | 0.73 | 0.64 | | 0.68 | 0.75 | 0.64 | 0.69 | 80 |
|  | *Stress* | 0.58 | 0.68 | | 0.63 | 0.59 | 0.71 | 0.65 | 59 |
|  | *Accuracy* |  |  | | 0.65 |  |  | 0.67 | 139 |
|  | *Weighted Average* | 0.67 | 0.65 | | 0.66 | 0.68 | 0.67 | 0.67 | 139 |
|  | *Macro Average* | 0.65 | 0.66 | | 0.65 | 0.67 | 0.67 | 0.67 | 139 |
| Age 25-34 | *No Stress* | 0.70 | 0.69 | | 0.70 | 0.68 | 0.82 | 0.74 | 72 |
|  | *Stress* | 0.76 | 0.77 | | 0.76 | 0.83 | 0.69 | 0.75 | 90 |
|  | *Accuracy* |  |  | | 0.73 |  |  | 0.75 | 162 |
|  | *Weighted Average* | 0.73 | 0.73 | | 0.73 | 0.76 | 0.75 | 0.75 | 162 |
|  | *Macro Average* | 0.73 | 0.73 | | 0.73 | 0.75 | 0.75 | 0.75 | 162 |
| Age 25-34 (SMOTE) | *No Stress* | 0.63 | 0.79 | | 0.70 | 0.67 | 0.83 | 0.74 | 72 |
|  | *Stress* | 0.79 | 0.63 | | 0.70 | 0.83 | 0.67 | 0.74 | 90 |
|  | *Accuracy* |  |  | | 0.70 |  |  | 0.74 | 162 |
|  | *Weighted Average* | 0.72 | 0.70 | | 0.70 | 0.76 | 0.74 | 0.74 | 162 |
|  | *Macro Average* | 0.71 | 0.71 | | 0.70 | 0.75 | 0.75 | 0.74 | 162 |
| Age 35-44 | *No Stress* | 0.78 | 0.91 | | 0.84 | 0.78 | 0.91 | 0.84 | 99 |
|  | *Stress* | 0.74 | 0.50 | | 0.60 | 0.75 | 0.52 | 0.61 | 52 |
|  | *Accuracy* |  |  | | 0.77 |  |  | 0.77 | 151 |
|  | *Weighted Average* | 0.76 | 0.77 | | 0.75 | 0.77 | 0.77 | 0.76 | 151 |
|  | *Macro Average* | 0.76 | 0.70 | | 0.72 | 0.77 | 0.71 | 0.73 | 151 |
| Age 35-44 (SMOTE) | *No Stress* | 0.79 | 0.88 | | 0.83 | 0.78 | 0.77 | 0.78 | 99 |
|  | *Stress* | 0.71 | 0.56 | | 0.62 | 0.57 | 0.60 | 0.58 | 52 |
|  | *Accuracy* |  |  | | 0.77 |  |  | 0.71 | 151 |
|  | *Weighted Average* | 0.76 | 0.77 | | 0.76 | 0.71 | 0.71 | 0.71 | 151 |
|  | *Macro Average* | 0.75 | 0.72 | | 0.73 | 0.68 | 0.68 | 0.68 | 151 |
| Healthy | *No Stress* | 0.75 | 0.86 | | 0.80 | 0.75 | 0.80 | 0.78 | 243 |
|  | *Stress* | 0.73 | 0.56 | | 0.63 | 0.66 | 0.59 | 0.62 | 157 |
|  | *Accuracy* |  |  | | 0.74 |  |  | 0.72 | 400 |
|  | *Weighted Average* | 0.74 | 0.74 | | 0.74 | 0.72 | 0.72 | 0.72 | 400 |
|  | *Macro Average* | 0.74 | 0.71 | | 0.72 | 0.71 | 0.70 | 0.70 | 400 |
| Healthy (SMOTE) | *No Stress* | 0.75 | 0.80 | | 0.78 | 0.80 | 0.75 | 0.77 | 243 |
|  | *Stress* | 0.66 | 0.59 | | 0.62 | 0.65 | 0.71 | 0.67 | 157 |
|  | *Accuracy* |  |  | | 0.72 |  |  | 0.73 | 400 |
|  | *Weighted Average* | 0.72 | 0.72 | | 0.72 | 0.74 | 0.73 | 0.73 | 400 |
|  | *Macro Average* | 0.71 | 0.70 | | 0.70 | 0.72 | 0.73 | 0.72 | 400 |
| **SDS** | **RF** | | | | | **SVM** | | |  |
|  | **Items** | ***Precision*** | | ***Recall*** | ***F1-Score*** | ***Precision*** | ***Recall*** | ***F1-Score*** |  |
| Complete Dataset | *No Stress* | 0.73 | | 0.81 | 0.77 | 0.73 | 0.78 | 0.76 | 249 |
|  | *Stress* | 0.69 | | 0.59 | 0.64 | 0.66 | 0.60 | 0.63 | 179 |
|  | *Accuracy* | - | | - | 0.72 | - | - | 0.71 | 428 |
|  | *Weighted Average* | 0.72 | | 0.72 | 0.72 | 0.70 | 0.71 | 0.70 | 428 |
|  | *Macro Average* | 0.71 | | 0.70 | 0.70 | 0.70 | 0.69 | 0.69 | 428 |
| Complete Dataset (SMOTE) | *No Stress* | 0.78 | | 0.69 | 0.73 | 0.78 | 0.72 | 0.75 | 249 |
|  | *Stress* | 0.63 | | 0.73 | 0.68 | 0.65 | 0.71 | 0.68 | 179 |
|  | *Accuracy* | - | | - | 0.71 | - | - | 0.72 | 428 |
|  | *Weighted Average* | 0.72 | | 0.71 | 0.71 | 0.72 | 0.72 | 0.72 | 428 |
|  | *Macro Average* | 0.70 | | 0.71 | 0.70 | 0.71 | 0.72 | 0.71 | 428 |
| Gender - Male | *No Stress* | 0.81 | | 0.86 | 0.83 | 0.81 | 0.85 | 0.83 | 73 |
|  | *Stress* | 0.09 | | 0.06 | 0.07 | 0.08 | 0.06 | 0.07 | 16 |
|  | *Accuracy* |  | |  | 0.72 |  |  | 0.71 | 89 |
|  | *Weighted Average* | 0.68 | | 0.72 | 0.70 | 0.68 | 0.71 | 0.69 | 89 |
|  | *Macro Average* | 0.45 | | 0.46 | 0.45 | 0.44 | 0.46 | 0.45 | 89 |
| Gender – Male (SMOTE) | *No Stress* | 0.81 | | 0.82 | 0.82 | 0.84 | 0.59 | 0.69 | 73 |
|  | *Stress* | 0.13 | | 0.12 | 0.13 | 0.21 | 0.50 | 0.30 | 16 |
|  | *Accuracy* |  | |  | 0.70 |  |  | 0.57 | 89 |
|  | *Weighted Average* | 0.69 | | 0.70 | 0.69 | 0.73 | 0.57 | 0.62 | 89 |
|  | *Macro Average* | 0.47 | | 0.47 | 0.47 | 0.53 | 0.54 | 0.49 | 89 |
| Gender – Female | *No Stress* | 0.72 | | 0.72 | 0.72 | 0.71 | 0.74 | 0.72 | 160 |
|  | *Stress* | 0.71 | | 0.70 | 0.70 | 0.71 | 0.68 | 0.70 | 153 |
|  | *Accuracy* |  | |  | 0.71 |  |  | 0.71 | 313 |
|  | *Weighted Average* | 0.71 | | 0.71 | 0.71 | 0.71 | 0.71 | 0.71 | 313 |
|  | *Macro Average* | 0.71 | | 0.71 | 0.71 | 0.71 | 0.71 | 0.71 | 313 |
| Gender – Female (SMOTE) | *No Stress* | 0.72 | | 0.72 | 0.72 | 0.71 | 0.72 | 0.72 | 160 |
|  | *Stress* | 0.71 | | 0.71 | 0.71 | 0.71 | 0.69 | 0.70 | 153 |
|  | *Accuracy* |  | |  | 0.71 |  |  | 0.71 | 313 |
|  | *Weighted Average* | 0.71 | | 0.71 | 0.71 | 0.71 | 0.71 | 0.71 | 313 |
|  | *Macro Average* | 0.71 | | 0.71 | 0.71 | 0.71 | 0.71 | 0.71 | 313 |
| Employment - Student | *No Stress* | 0.76 | | 0.71 | 0.73 | 0.75 | 0.74 | 0.74 | 99 |
|  | *Stress* | 0.71 | | 0.76 | 0.73 | 0.72 | 0.74 | 0.73 | 92 |
|  | *Accuracy* |  | |  | 0.73 |  |  | 0.74 | 191 |
|  | *Weighted Average* | 0.73 | | 0.73 | 0.73 | 0.74 | 0.74 | 0.74 | 191 |
|  | *Macro Average* | 0.73 | | 0.73 | 0.73 | 0.74 | 0.74 | 0.74 | 191 |
| Employment – Student (SMOTE) | *No Stress* | 0.75 | | 0.66 | 0.70 | 0.77 | 0.72 | 0.74 | 99 |
|  | *Stress* | 0.67 | | 0.76 | 0.71 | 0.72 | 0.77 | 0.74 | 92 |
|  | *Accuracy* |  | |  | 0.71 |  |  | 0.74 | 191 |
|  | *Weighted Average* | 0.71 | | 0.71 | 0.71 | 0.75 | 0.74 | 0.74 | 191 |
|  | *Macro Average* | 0.71 | | 0.71 | 0.71 | 0.74 | 0.74 | 0.74 | 191 |
| Employment – Worker | *No Stress* | 0.73 | | 0.85 | 0.79 | 0.73 | 0.79 | 0.76 | 151 |
|  | *Stress* | 0.64 | | 0.45 | 0.53 | 0.58 | 0.51 | 0.54 | 87 |
|  | *Accuracy* |  | |  | 0.71 |  |  | 0.68 | 238 |
|  | *Weighted Average* | 0.70 | | 0.71 | 0.69 | 0.68 | 0.68 | 0.68 | 238 |
|  | *Macro Average* | 0.68 | | 0.65 | 0.66 | 0.66 | 0.65 | 0.65 | 238 |
| Employment – Worker (SMOTE) | *No Stress* | 0.73 | | 0.72 | 0.72 | 0.73 | 0.68 | 0.70 | 151 |
|  | *Stress* | 0.53 | | 0.55 | 0.54 | 0.50 | 0.56 | 0.53 | 87 |
|  | *Accuracy* |  | |  | 0.66 |  |  | 0.63 | 238 |
|  | *Weighted Average* | 0.66 | | 0.66 | 0.66 | 0.65 | 0.63 | 0.64 | 238 |
|  | *Macro Average* | 0.63 | | 0.63 | 0.63 | 0.61 | 0.62 | 0.62 | 238 |
| Income - Low | *No Stress* | 0.76 | | 0.78 | 0.77 | 0.78 | 0.78 | 0.78 | 125 |
|  | *Stress* | 0.63 | | 0.60 | 0.61 | 0.64 | 0.64 | 0.64 | 78 |
|  | *Accuracy* |  | |  | 0.71 |  |  | 0.72 | 203 |
|  | *Weighted Average* | 0.71 | | 0.71 | 0.71 | 0.72 | 0.72 | 0.72 | 203 |
|  | *Macro Average* | 0.69 | | 0.69 | 0.69 | 0.71 | 0.71 | 0.71 | 203 |
| Income – Low (SMOTE) | *No Stress* | 0.81 | | 0.76 | 0.79 | 0.82 | 0.74 | 0.78 | 125 |
|  | *Stress* | 0.65 | | 0.72 | 0.68 | 0.64 | 0.74 | 0.69 | 78 |
|  | *Accuracy* |  | |  | 0.74 |  |  | 0.74 | 203 |
|  | *Weighted Average* | 0.75 | | 0.74 | 0.75 | 0.75 | 0.74 | 0.74 | 203 |
|  | *Macro Average* | 0.73 | | 0.74 | 0.73 | 0.73 | 0.74 | 0.73 | 203 |
| Income – Medium High | *No Stress* | 0.71 | | 0.69 | 0.70 | 0.70 | 0.71 | 0.71 | 110 |
|  | *Stress* | 0.62 | | 0.64 | 0.63 | 0.62 | 0.62 | 0.62 | 86 |
|  | *Accuracy* |  | |  | 0.67 |  |  | 0.67 | 196 |
|  | *Weighted Average* | 0.67 | | 0.67 | 0.67 | 0.67 | 0.67 | 0.67 | 196 |
|  | *Macro Average* | 0.66 | | 0.67 | 0.66 | 0.66 | 0.66 | 0.66 | 196 |
| Income – Medium High (SMOTE) | *No Stress* | 0.69 | | 0.65 | 0.67 | 0.70 | 0.68 | 0.69 | 110 |
|  | *Stress* | 0.58 | | 0.62 | 0.60 | 0.61 | 0.63 | 0.62 | 86 |
|  | *Accuracy* |  | |  | 0.64 |  |  | 0.66 | 196 |
|  | *Weighted Average* | 0.64 | | 0.64 | 0.64 | 0.66 | 0.66 | 0.66 | 196 |
|  | *Macro Average* | 0.63 | | 0.64 | 0.63 | 0.65 | 0.65 | 0.65 | 196 |
| Age – 18-24 | *No Stress* | 0.72 | | 0.62 | 0.67 | 0.66 | 0.61 | 0.63 | 71 |
|  | *Stress* | 0.58 | | 0.69 | 0.63 | 0.54 | 0.60 | 0.57 | 55 |
|  | *Accuracy* |  | |  | 0.65 |  |  | 0.60 | 126 |
|  | *Weighted Average* | 0.66 | | 0.65 | 0.65 | 0.61 | 0.60 | 0.60 | 126 |
|  | *Macro Average* | 0.65 | | 0.66 | 0.65 | 0.60 | 0.60 | 0.60 | 126 |
| Age – 18-24 (SMOTE) | *No Stress* | 0.74 | | 0.59 | 0.66 | 0.67 | 0.62 | 0.64 | 71 |
|  | *Stress* | 0.58 | | 0.73 | 0.65 | 0.55 | 0.60 | 0.57 | 55 |
|  | *Accuracy* |  | |  | 0.65 |  |  | 0.61 | 126 |
|  | *Weighted Average* | 0.67 | | 0.65 | 0.65 | 0.63 | 0.62 | 0.61 | 126 |
|  | *Macro Average* | 0.66 | | 0.66 | 0.65 | 0.62 | 0.61 | 0.61 | 126 |
| Age 25-34 | *No Stress* | 0.67 | | 0.58 | 0.62 | 0.67 | 0.58 | 0.62 | 67 |
|  | *Stress* | 0.69 | | 0.76 | 0.72 | 0.69 | 0.76 | 0.72 | 80 |
|  | *Accuracy* |  | |  | 0.68 |  |  | 0.68 | 147 |
|  | *Weighted Average* | 0.68 | | 0.68 | 0.68 | 0.68 | 0.68 | 0.68 | 147 |
|  | *Macro Average* | 0.68 | | 0.67 | 0.67 | 0.68 | 0.67 | 0.67 | 147 |
| Age 25-34 (SMOTE) | *No Stress* | 0.67 | | 0.63 | 0.65 | 0.67 | 0.64 | 0.66 | 67 |
|  | *Stress* | 0.70 | | 0.74 | 0.72 | 0.71 | 0.74 | 0.72 | 80 |
|  | *Accuracy* |  | |  | 0.69 |  |  | 0.69 | 147 |
|  | *Weighted Average* | 0.69 | | 0.69 | 0.69 | 0.69 | 0.69 | 0.69 | 147 |
|  | *Macro Average* | 0.68 | | 0.68 | 0.68 | 0.69 | 0.69 | 0.69 | 147 |
| Age 35-44 | *No Stress* | 0.81 | | 0.87 | 0.84 | 0.81 | 0.84 | 0.83 | 82 |
|  | *Stress* | 0.42 | | 0.32 | 0.46 | 0.41 | 0.36 | 0.38 | 25 |
|  | *Accuracy* |  | |  | 0.74 |  |  | 0.73 | 107 |
|  | *Weighted Average* | 0.72 | | 0.74 | 0.73 | 0.72 | 0.73 | 0.72 | 107 |
|  | *Macro Average* | 0.61 | | 0.59 | 0.60 | 0.61 | 0.60 | 0.60 | 107 |
| Age 35-44 (SMOTE) | *No Stress* | 0.84 | | 0.80 | 0.82 | 0.80 | 0.60 | 0.69 | 82 |
|  | *Stress* | 0.43 | | 0.48 | 0.45 | 0.28 | 0.52 | 0.37 | 25 |
|  | *Accuracy* |  | |  | 0.73 |  |  | 0.58 | 107 |
|  | *Weighted Average* | 0.74 | | 0.73 | 0.73 | 0.68 | 0.58 | 0.61 | 107 |
|  | *Macro Average* | 0.63 | | 0.64 | 0.64 | 0.54 | 0.56 | 0.53 | 107 |
| Healthy | *No Stress* | 0.71 | | 0.71 | 0.71 | 0.67 | 0.82 | 0.74 | 55 |
|  | *Stress* | 0.75 | | 0.75 | 0.75 | 0.81 | 0.66 | 0.73 | 65 |
|  | *Accuracy* |  | |  | 0.73 |  |  | 0.73 | 120 |
|  | *Weighted Average* | 0.73 | | 0.73 | 0.73 | 0.74 | 0.74 | 0.73 | 120 |
|  | *Macro Average* | 0.73 | | 0.73 | 0.73 | 0.75 | 0.73 | 0.73 | 120 |
| Healthy (SMOTE) | *No Stress* | 0.67 | | 0.82 | 0.74 | 0.68 | 0.82 | 0.74 | 55 |
|  | *Stress* | 0.81 | | 0.66 | 0.73 | 0.81 | 0.68 | 0.74 | 65 |
|  | *Accuracy* |  | |  | 0.73 |  |  | 0.74 | 120 |
|  | *Weighted Average* | 0.75 | | 0.73 | 0.73 | 0.75 | 0.74 | 0.74 | 120 |
|  | *Macro Average* | 0.74 | | 0.74 | 0.73 | 0.75 | 0.75 | 0.74 | 120 |

Table B6: Review of Stress Prediction ML Studies

| **Study** | **Year** | **Type** | **Device** | **Variables** | **Size** | **Period** | **Ground Truth** | **Model** | **G/I** | **Accuracy** | **Validation** |
| --- | --- | --- | --- | --- | --- | --- | --- | --- | --- | --- | --- |
| [1] | 2013 | DDSR | Wahoo chest belt | Audio, Physical Activity, HRV, Communication | 35 | 4 Months | PANAS self-report questionnaire + audio self-report + stress self-assessment before sleep | LR | G/I | Generalized: 53%  Individualized: 61% | Generalized Leave One Person Out  Individualized Leave One Day Out |
| [2] | 2010 | LLKC | Emotion Board | EDA | 33 | N/A | Known context | LDA, SVM, NCC | G | 83% (LDA), ˜81% (SVM) | Leave One Person Out |
| [3] | 2020 | DDSR | Empatica E4 | EDA, BVP, Acc, HR, Skin Temperature | 6 | 4 Weeks | Button Press from device indicating a stressful event | RF, SVM | G | 87.4% (RF), 82.1% (SVM) - calculated as AUC | 10-fold Cross Validation, testing on 10% validation set |
| [4] | 2012 | LLSR | Polar WearLink+, SA9311M, AgCI electrodes | HRV, Respiration, EDA | 10 | N/A | 7-point LIKERT scale on perceived stress levels | LR | I | 81% | Leave One Person Out |
| [5] | 2020 | Mix of different modalities | Empatica E4 | EDA, HRV, HR | 14 | 1 week | DDSR: PSS-5 and EMA LDSR: PSS-5 and known context. | MLP, RF, kNN, SVM, LR | G | DDSR: 68% (SVM), 64% (LR), 60% (kNN), 52% (RF)  LDSR: 74.6% (RF), 73.8% (LR), 73.4% (SVM), kNN AND MLP (72.2%) | 10-fold CV with data from all users  80% training, 20% testing |
| [6] | 2014 | DDSR | BioHarness 3.0 + , Empatica E4 | ECG, HRV, Resp, Temp, GSR, Posture, Accelerometer, Sleep | 10 | 18 days | 2 questions before sleep on how the participant felt during the day | SVM, LR, kNN, RF, NN | G | 73% (SVM), 71% (RF), NN (˜63%), kNN (˜60%), LR (52%) | Leave One Person Out |
| [7] | 2017 | LLKC | NeuLog (ECG, GSR, Resp), Contec PM50 (BP), Kenek Edge pulse oximeter (SpO2) | ECG, Resp, GSR, BP, SpO2 | 32 | N/A | Known context | SVM, kNN | G/I | Generalized: 89.2% (kNN), 83.1% (SVM) Individualized: 94.5% (kNN), 86.7% (SVM) | Train, Test, Validation |
| [8] | 2015 | LLKC and LLSR | Sensor suite similar to the BioHarness | ECG, Resp | 20 | 1 week | Known context in lab, PSS-5 with EMA in the field | SVM | G | LLKC: 90%  LLSR: 72% | Leave One Person Out |
| [9] | 2016 | LLKC and LLSR | Empatica E4 | BVP, HR, HRV, ST and GSR | 5 | 55 days | Known context and Short STAY-Y Questionnaire in the laboratory, 4-6 random EMA prompts asking users for the duration and the level of stress in the field | RF for laboratory stress followed by SVM for real-life data | I | 92% (SVM) | Leave One Person Out |
| [10] | 2018 | LLKC | NeuroSky Mindwave Mobile | EEG | 7 | N/A | Known context while listening to music (meditation and attention instead of stress/non-stress) | Neural Network | G | 0.60 (Attention), 0.01 (Meditation) – calculated as F1-score | Train, Test and Validation |
| [11] | 2013 | LLSR | Androind Phone + Wristworn Sensor | ACC, Skin Conductance, Phone Usage | 18 | 5 days | PSS, PSQI, Big Five Inventory Personality Test | SVM, kNN | G | Over 75% (with a number of trained model and feature combinations) | 10-fold CV with data from all users  90% training, 10% testing |
| [12] | 2015 | DDSR | Samsung Galaxy SIII Mini | ACC | 30 | 8 weeks | OLBI + 3 EMA prompts per day asking users to rate stress on a 1-5 scale | Naïve Bayes, DT, Ordinal Naïve Bayes | G/I | Generalized:  Accuracy - 52%  MAE – 0.83  RMSE – 1  Individualized:  Accuracy - 71%  MAE – 0.66  RMSE – 0.96 | 5-fold CV for Individualized, Leave One Person Out for Generalized |
| [13] | 2019 | DDSR and DDKC (in a structured programming contest) | Samsung Gear S1, S2 and S3, Empatica E4 | HRV, ACC, EDA | 21 | 9 days | Frustration collected in the NASA-TLX questionnaire + 0-100 scale with question, 3 class stress | PCA + LDA, SVM, LR, RF, Multilayer Perceptrom, | G/I | Generalized: 88.20% (RF) with DDKC, 86.38% with DDSR  Individualized:  97.92% (RF) | Train amd Test for Individualized, 10-fold CV for Generalized |
| [14] | 2018 | LLKC | BioPatch M3 | HRV | 128 | N/A | Known Context (, Stroop Colour Word Test, videogames) | SVM | G | 64% | 10-fold CV |
| [15] | 2016 | LLKC | Emotiv Epoch headset | EEG | 6 | N/A | Mathematical Questions, self-report with the NASA-TLX | SVM, LDA, QDA, kNN | G | 89% (SVM) | 10-fold CV |
| [16] | 2015 | LLKC | BioNomadix model BN-PPGED | EDA, HRV | 5 | N/A | State Trait Anxiety Inventory, Triet Social Stress Test | SVM | I | For each participant:  78.90%, 73.26%, 83.08%, 82.82%, 76.83% | Train and Test (75% and 25%) |

Table B7: Generalized Model - RF Feature Importance, D

| **All** | |
| --- | --- |
| **Feature** | **Values** |
| MAP | 0.018 |
| ECG_DC | 0.016 |
| User | 0.015 |
| ECG_AR_AbsolutePower_HF | 0.014 |
| ECG_AR_AbsolutePower_LF | 0.014 |
| dia | 0.013 |
| HRV-1 | 0.013 |
| Empatica_AR_RelativePower_LF | 0.013 |
| Empatica_AR_LFHF | 0.012 |
| ECG_Stress Index | 0.012 |
| **Gender - Male** | |
| **Feature** | **Values** |
| MAP | 0.032 |
| AW Min HR - Interval | 0.027 |
| Empatica_SampEn | 0.021 |
| Empatica_MSE13 | 0.018 |
| Empatica_MSE17 | 0.017 |
| sys | 0.016 |
| Empatica_MSE3 | 0.016 |
| Empatica_MSE2 | 0.016 |
| Short Term Min | 0.016 |
| Weight | 0.016 |
| **Gender - Female** | |
| **Feature** | **Values** |
| User | 0.038 |
| Weight | 0.022 |
| ECG_SNS_Index | 0.020 |
| ECG_DC | 0.019 |
| ECG_SDNN | 0.019 |
| ECG_AC | 0.016 |
| ECG_RMSSD | 0.014 |
| Empatica_FFT_LFHF | 0.014 |
| ECG_FFT_AbsolutePower_HF | 0.014 |
| ECG_AR_AbsolutePower_HF | 0.013 |
| **Income - Low** | |
| **Feature** | **Values** |
| ECG_Dcmod | 0.019 |
| Empatica_MSE2 | 0.019 |
| MAP | 0.018 |
| ECG_RMSSD | 0.018 |
| User | 0.017 |
| ECG_SDNN | 0.017 |
| ECG_Stress Index | 0.016 |
| Empatica_MSE12 | 0.016 |
| Empatica_DC | 0.015 |
| ECG_DC | 0.014 |
| **Income Medium High** | |
| **Feature** | **Values** |
| ECG_DC | 0.021 |
| ECG_SD1SD2 | 0.016 |
| ECG_AC | 0.015 |
| dia | 0.014 |
| Empatica_DC | 0.014 |
| Empatica_FFT_RelativePower_VLF | 0.014 |
| HRV-1 | 0.014 |
| ECG_AR_AbsolutePower_HF | 0.013 |
| Empatica_FFT_AbsolutePower_HF | 0.013 |
| Empatica_FFT_HF | 0.012 |
| **Employment Students** | |
| **Feature** | **Values** |
| User | 0.042 |
| ECG_RMSSD | 0.023 |
| MAP | 0.022 |
| ECG_Dcmod | 0.021 |
| ECG_Stress Index | 0.021 |
| ECG_DC | 0.020 |
| ECG_FFT_TotalPower | 0.019 |
| ECG_FFT_AbsolutePower_LF | 0.018 |
| ECG_SNS_Index | 0.017 |
| ECG_AR_AbsolutePower_HF | 0.017 |
| **Employment Workers** | |
| **Feature** | **Values** |
| MAP | 0.027 |
| Weight | 0.017 |
| sys | 0.015 |
| AW Max Steps | 0.015 |
| HRV-1 | 0.014 |
| Empatica_FFT_RelativePower_VLF | 0.014 |
| Empatica_MSE11 | 0.014 |
| ECG_DC | 0.014 |
| Empatica_Max HR | 0.014 |
| AW Mean Steps | 0.014 |
| **Age 18-24** | |
| **Feature** | **Values** |
| Weight | 0.026 |
| sys | 0.024 |
| Empatica_Mean line length | 0.018 |
| Empatica_MSE2 | 0.016 |
| ECG_FFT_RelativePower_LF | 0.015 |
| Empatica_Min HR | 0.015 |
| AW Max Steps | 0.015 |
| AW Mean Steps | 0.014 |
| ECG_FFT_LF | 0.014 |
| ECG_FFT_AbsolutePower_LF | 0.014 |
| **Age 25-34** | |
| **Feature** | **Values** |
| MAP | 0.021 |
| HRV-1 | 0.021 |
| Weight | 0.021 |
| Empatica_MSE4 | 0.016 |
| Empatica_FFT_RelativePower_VLF | 0.015 |
| Temp | 0.014 |
| ECG_SD HR | 0.014 |
| ECG_DC | 0.013 |
| ECG_AR_AbsolutePower_LF | 0.013 |
| ECG_AC | 0.013 |
| **Age 35-44** | |
| **Feature** | **Values** |
| Temp | 0.019 |
| User | 0.018 |
| ECG_AR_LFHF | 0.016 |
| ECG_AC | 0.015 |
| Empatica_MSE15 | 0.015 |
| AW Mean Steps | 0.015 |
| ECG_AR_RelativePower_LF | 0.015 |
| Empatica_FFT_HF | 0.015 |
| Empatica_alpha2 | 0.014 |
| ECG_DC | 0.014 |
| **Healthy** | |
| **Feature** | **Values** |
| MAP | 0.023 |
| ECG_DC | 0.017 |
| ECG_AR_HF | 0.017 |
| ECG_AR_AbsolutePower_HF | 0.015 |
| Empatica_MSE2 | 0.014 |
| ECG_FFT_AbsolutePower_HF | 0.014 |
| AW Min HR | 0.014 |
| Empatica_MSE3 | 0.014 |
| sys | 0.013 |
| ECG_SDNN | 0.013 |

Table B8: Generalized Model - RF Feature Importance, DECG

| **All** | |
| --- | --- |
| **Feature** | **Values** |
| User | 0.11 |
| ECG_DC | 0.049 |
| ECG_AR_AbsolutePower_HF | 0.044 |
| ECG_Max HR | 0.043 |
| ECG_FFT_AbsolutePower_HF | 0.041 |
| ECG_SDNN | 0.040 |
| ECG_AC | 0.040 |
| ECG_Stress Index | 0.040 |
| ECG_FFT_RelativePower_LF | 0.039 |
| ECG_SNS_Index | 0.038 |
| **Gender - Male** | |
| **Feature** | **Values** |
| User | 0.137 |
| ECG_Mean RR | 0.064 |
| ECG_Mean HR | 0.053 |
| ECG_PNS Index | 0.052 |
| ECG_AR_HF | 0.049 |
| ECG_SD1SD2 | 0.042 |
| ECG_RMSSD | 0.040 |
| ECG_AR_AbsolutePower_LF | 0.037 |
| ECG_DC | 0.036 |
| ECG_AR_AbsolutePower_HF | 0.036 |
| **Gender - Female** | |
| **Feature** | **Values** |
| User | 0.067 |
| ECG_DC | 0.044 |
| ECG_AC | 0.044 |
| ECG_AR_AbsolutePower_HF | 0.044 |
| ECG_Stress Index | 0.044 |
| ECG_FFT_AbsolutePower_HF | 0.043 |
| ECG_SDNN | 0.042 |
| ECG_Max HR | 0.042 |
| ECG_Mean RR | 0.041 |
| ECG_SNS_Index | 0.041 |
| **Income - Low** | |
| **Feature** | **Values** |
| User | 0.045 |
| ECG_Stress Index | 0.042 |
| ECG_DC | 0.042 |
| ECG_AC | 0.041 |
| ECG_AR_AbsolutePower_HF | 0.041 |
| ECG_Dcmod | 0.039 |
| ECG_RMSSD | 0.039 |
| ECG_SD HR | 0.039 |
| ECG_AR_AbsolutePower_LF | 0.039 |
| ECG_FFT_LFHF | 0.039 |
| **Income Medium High** | |
| **Feature** | **Values** |
| User | 0.158 |
| ECG_DC | 0.052 |
| ECG_AC | 0.045 |
| ECG_AR_AbsolutePower_LF | 0.041 |
| ECG_SD1SD2 | 0.040 |
| ECG_SDNN | 0.038 |
| ECG_Mean RR | 0.038 |
| ECG_Stress Index | 0.036 |
| ECG_FFT_AbsolutePower_HF | 0.034 |
| ECG_SD HR | 0.034 |
| **Employment Students** | |
| **Feature** | **Values** |
| User | 0.094 |
| ECG_AR_AbsolutePower_HF | 0.047 |
| ECG_RMSSD | 0.042 |
| ECG_DC | 0.041 |
| ECG_SD HR | 0.040 |
| ECG_Stress Index | 0.040 |
| ECG_Dcmod | 0.040 |
| ECG_FFT_RelativePower_LF | 0.039 |
| ECG_FFT_LFHF | 0.038 |
| ECG_AC | 0.037 |
| **Employment Workers** | |
| **Feature** | **Values** |
| User | 0.110 |
| ECG_SDNN | 0.044 |
| ECG_AR_AbsolutePower_LF | 0.041 |
| ECG_AC | 0.040 |
| ECG_SD1SD2 | 0.039 |
| ECG_Max HR | 0.039 |
| ECG_DC | 0.039 |
| ECG_Mean RR | 0.038 |
| ECG_PNS Index | 0.038 |
| ECG_Stress Index | 0.037 |
| **Age 18-24** | |
| **Feature** | **Values** |
| ECG_AR_HF | 0.049 |
| ECG_SD1SD2 | 0.045 |
| ECG_FFT_HF | 0.045 |
| ECG_SD HR | 0.043 |
| ECG_Mean RR | 0.042 |
| ECG_FFT_AbsolutePower_LF | 0.041 |
| ECG_Mean HR | 0.041 |
| ECG_AR_AbsolutePower_HF | 0.041 |
| ECG_AC | 0.040 |
| ECG_FFT_LF | 0.040 |
| **Age 25-34** | |
| **Feature** | **Values** |
| User | 0.048 |
| ECG_DC | 0.042 |
| ECG_AR_LFHF | 0.042 |
| ECG_AR_AbsolutePower_HF | 0.042 |
| ECG_FFT_LF | 0.042 |
| ECG_AR_AbsolutePower_LF | 0.042 |
| ECG_Mean RR | 0.041 |
| ECG_FFT_LFHF | 0.040 |
| ECG_SNS_Index | 0.040 |
| ECG_AR_RelativePower_LF | 0.040 |
| **Age 35-44** | |
| **Feature** | **Values** |
| User | 0.083 |
| ECG_SD1SD2 | 0.047 |
| ECG_AR_HF | 0.046 |
| ECG_Mean RR | 0.039 |
| ECG_AR_RelativePower_LF | 0.038 |
| ECG_SD HR | 0.038 |
| ECG_AC | 0.038 |
| ECG_FFT_RelativePower_LF | 0.037 |
| ECG_DC | 0.036 |
| ECG_FFT_HF | 0.036 |
| **Healthy** | |
| **Feature** | **Values** |
| User | 0.053 |
| ECG_AC | 0.044 |
| ECG_DC | 0.043 |
| ECG_AR_AbsolutePower_HF | 0.041 |
| ECG_FFT_AbsolutePower_HF | 0.039 |
| ECG_SDNN | 0.039 |
| ECG_SD1SD2 | 0.039 |
| ECG_FFT_LFHF | 0.038 |
| ECG_FFT_RelativePower_LF | 0.038 |
| ECG_Stress Index | 0.038 |

Table B9: Generalized Model - RF Feature Importance, DA

| **All** | |
| --- | --- |
| **Feature** | **Values** |
| User | 0.052 |
| AW Mean Steps | 0.033 |
| HRV-1 | 0.033 |
| ECG_AC | 0.033 |
| AW Mean HR - Interval | 0.033 |
| AW Min HR - Interval | 0.032 |
| ECG_DC | 0.031 |
| ECG_SDNN | 0.030 |
| AW Max Steps | 0.030 |
| ECG_Max HR | 0.029 |
| **Gender - Male** | |
| **Feature** | **Values** |
| User | 0.091 |
| AW Min HR - Interval | 0.046 |
| ECG_Mean RR | 0.036 |
| ECG_Mean HR | 0.036 |
| AW Mean HR - Interval | 0.035 |
| HRV-1 | 0.034 |
| ECG_AR_HF | 0.031 |
| ECG_SD1SD2 | 0.031 |
| ECG_PNS Index | 0.029 |
| ECG_RMSSD | 0.027 |
| **Gender - Female** | |
| **Feature** | **Values** |
| User | 0.053 |
| ECG_SDNN | 0.036 |
| ECG_DC | 0.036 |
| ECG_FFT_AbsolutePower_HF | 0.036 |
| ECG_AC | 0.035 |
| ECG_Stress Index | 0.034 |
| ECG_AR_AbsolutePower_HF | 0.034 |
| AW Mean Steps | 0.032 |
| ECG_RMSSD | 0.031 |
| ECG_SD HR | 0.031 |
| **Income - Low** | |
| **Feature** | **Values** |
| AW Mean Steps | 0.035 |
| User | 0.034 |
| HRV-1 | 0.032 |
| ECG_AC | 0.032 |
| ECG_Stress Index | 0.032 |
| ECG_DC | 0.030 |
| AW Min HR - Interval | 0.030 |
| AW Max Steps | 0.030 |
| ECG_RMSSD | 0.029 |
| AW Mean HR | 0.029 |
| **Income Medium High** | |
| **Feature** | **Values** |
| User | 0.075 |
| ECG_DC | 0.035 |
| AW Mean HR - Interval | 0.034 |
| ECG_AC | 0.032 |
| HRV-1 | 0.031 |
| Short Term Min | 0.030 |
| ECG_SD1SD2 | 0.030 |
| AW Min HR - Interval | 0.029 |
| AW Max HR - Interval | 0.028 |
| ECG_SD HR | 0.028 |
| **Employment Students** | |
| **Feature** | **Values** |
| User | 0.116 |
| ECG_RMSSD | 0.041 |
| ECG_Stress Index | 0.038 |
| ECG_DC | 0.036 |
| ECG_SDNN | 0.036 |
| ECG_Dcmod | 0.036 |
| ECG_AR_AbsolutePower_HF | 0.033 |
| HRV-1 | 0.031 |
| ECG_AC | 0.030 |
| ECG_SD HR | 0.029 |
| **Employment Workers** | |
| **Feature** | **Values** |
| User | 0.103 |
| AW Mean HR - Interval | 0.039 |
| HRV-1 | 0.034 |
| AW Min HR - Interval | 0.031 |
| ECG_SDNN | 0.030 |
| AW Mean Steps | 0.029 |
| ECG_AR_AbsolutePower_LF | 0.029 |
| ECG_RMSSD | 0.028 |
| ECG_DC | 0.028 |
| AW Max Steps | 0.028 |
| **Age 18-24** | |
| **Feature** | **Values** |
| AW Mean Steps | 0.048 |
| ECG_AR_HF | 0.041 |
| ECG_FFT_HF | 0.038 |
| AW Max HR | 0.033 |
| HRV-1 | 0.032 |
| ECG_FFT_LFHF | 0.031 |
| AW Max Steps | 0.031 |
| ECG_SD1SD2 | 0.031 |
| AW Mean HR - Interval | 0.030 |
| ECG_FFT_RelativePower_LF | 0.030 |
| **Age 25-34** | |
| **Feature** | **Values** |
| HRV-1 | 0.041 |
| User | 0.040 |
| AW Mean HR - Interval | 0.038 |
| ECG_AR_LFHF | 0.031 |
| ECG_DC | 0.031 |
| AW Mean Steps | 0.030 |
| ECG_SD HR | 0.030 |
| ECG_AR_AbsolutePower_HF | 0.030 |
| ECG_AR_RelativePower_LF | 0.029 |
| ECG_FFT_LFHF | 0.029 |
| **Age 35-44** | |
| **Feature** | **Values** |
| User | 0.068 |
| AW Max HR - Interval | 0.036 |
| ECG_SD1SD2 | 0.033 |
| ECG_AR_HF | 0.032 |
| ECG_AR_RelativePower_LF | 0.032 |
| HRV-1 | 0.031 |
| Short Term Min | 0.030 |
| AW Max Steps | 0.029 |
| ECG_AR_LFHF | 0.029 |
| AW Mean Steps | 0.029 |
| **Healthy** | |
| **Feature** | **Values** |
| User | 0.040 |
| HRV-1 | 0.034 |
| AW Mean Steps | 0.034 |
| AW Mean HR - Interval | 0.033 |
| ECG_AC | 0.033 |
| ECG_DC | 0.031 |
| AW Max HR - Interval | 0.030 |
| ECG_SD1SD2 | 0.030 |
| AW Max Steps | 0.030 |
| AW Min HR - Interval | 0.030 |

Table B10: Generalized Model - RF Feature Importance, DAW

| **All** | |
| --- | --- |
| **Feature** | **Values** |
| Weight | 0.047 |
| User | 0.042 |
| MAP | 0.034 |
| Temp | 0.031 |
| AW Mean Steps | 0.029 |
| HRV-1 | 0.028 |
| AW Mean HR - Interval | 0.028 |
| ECG_DC | 0.026 |
| dia | 0.026 |
| ECG_AC | 0.025 |
| **Gender - Male** | |
| **Feature** | **Values** |
| Weight | 0.058 |
| User | 0.051 |
| AW Min HR - Interval | 0.040 |
| Temp | 0.038 |
| MAP | 0.037 |
| AW Mean HR - Interval | 0.030 |
| ECG_SD1SD2 | 0.027 |
| ECG_AR_HF | 0.027 |
| dia | 0.026 |
| sys | 0.026 |
| **Gender - Female** | |
| **Feature** | **Values** |
| Weight | 0.041 |
| User | 0.039 |
| Temp | 0.030 |
| MAP | 0.030 |
| HRV-1 | 0.028 |
| ECG_AC | 0.028 |
| AW Mean Steps | 0.028 |
| AW Mean HR - Interval | 0.027 |
| ECG_DC | 0.026 |
| ECG_Stress Index | 0.026 |
| **Income - Low** | |
| **Feature** | **Values** |
| Weight | 0.038 |
| Temp | 0.035 |
| AW Min HR - Interval | 0.028 |
| MAP | 0.028 |
| AW Mean Steps | 0.027 |
| ECG_AC | 0.026 |
| User | 0.026 |
| ECG_SD HR | 0.026 |
| HRV-1 | 0.026 |
| ECG_FFT_AbsolutePower_HF | 0.026 |
| **Income Medium High** | |
| **Feature** | **Values** |
| User | 0.074 |
| Weight | 0.064 |
| Temp | 0.041 |
| AW Mean HR - Interval | 0.036 |
| dia | 0.034 |
| sys | 0.031 |
| HRV-1 | 0.027 |
| AW Min HR - Interval | 0.026 |
| ECG_SD1SD2 | 0.025 |
| AW Max HR - Interval | 0.024 |
| **Employment Students** | |
| **Feature** | **Values** |
| Weight | 0.110 |
| User | 0.074 |
| ECG_RMSSD | 0.033 |
| MAP | 0.032 |
| Temp | 0.032 |
| ECG_Stress Index | 0.032 |
| ECG_Dcmod | 0.029 |
| ECG_DC | 0.029 |
| ECG_SD HR | 0.027 |
| ECG_AR_AbsolutePower_HF | 0.027 |
| **Employment Workers** | |
| **Feature** | **Values** |
| User | 0.065 |
| Weight | 0.053 |
| Temp | 0.042 |
| AW Mean HR - Interval | 0.036 |
| MAP | 0.033 |
| AW Min HR - Interval | 0.030 |
| HRV-1 | 0.026 |
| ECG_SD1SD2 | 0.025 |
| sys | 0.025 |
| dia | 0.025 |
| **Age 18-24** | |
| **Feature** | **Values** |
| Weight | 0.061 |
| AW Mean Steps | 0.036 |
| ECG_AR_HF | 0.032 |
| Temp | 0.031 |
| ECG_FFT_HF | 0.028 |
| HRV-1 | 0.028 |
| ECG_FFT_LFHF | 0.027 |
| ECG_FFT_RelativePower_LF | 0.027 |
| AW Max Steps | 0.026 |
| AW Mean HR - Interval | 0.025 |
| **Age 25-34** | |
| **Feature** | **Values** |
| Weight | 0.063 |
| Temp | 0.032 |
| HRV-1 | 0.031 |
| MAP | 0.031 |
| AW Mean HR - Interval | 0.031 |
| AW Min HR - Interval | 0.030 |
| ECG_DC | 0.028 |
| ECG_AR_AbsolutePower_HF | 0.027 |
| User | 0.026 |
| ECG_FFT_LF | 0.025 |
| **Age 35-44** | |
| **Feature** | **Values** |
| Weight | 0.053 |
| User | 0.051 |
| ECG_SD1SD2 | 0.036 |
| ECG_AR_RelativePower_LF | 0.033 |
| dia | 0.031 |
| MAP | 0.031 |
| sys | 0.030 |
| ECG_AR_LFHF | 0.028 |
| HRV-1 | 0.027 |
| AW Max HR - Interval | 0.026 |
| **Healthy** | |
| **Feature** | **Values** |
| Weight | 0.054 |
| Temp | 0.035 |
| MAP | 0.032 |
| User | 0.027 |
| AW Mean Steps | 0.026 |
| HRV-1 | 0.026 |
| ECG_AC | 0.026 |
| AW Mean HR - Interval | 0.025 |
| sys | 0.025 |
| ECG_DC | 0.025 |

Table B11: Generalized Model - RF Feature Importance, DW

| **All** | |
| --- | --- |
| **Feature** | **Values** |
| Weight | 0.251 |
| User | 0.204 |
| Temp | 0.160 |
| MAP | 0.151 |
| dia | 0.118 |
| **Gender - Male** | |
| **Feature** | **Values** |
| Weight | 0.295 |
| User | 0.201 |
| Temp | 0.147 |
| MAP | 0.128 |
| dia | 0.122 |
| **Gender - Female** | |
| **Feature** | **Values** |
| Weight | 0.25 |
| User | 0.18 |
| Temp | 0.18 |
| MAP | 0.15 |
| sys | 0.12 |
| **Income - Low** | |
| **Feature** | **Values** |
| Weight | 0.235 |
| Temp | 0.193 |
| MAP | 0.166 |
| User | 0.139 |
| dia | 0.138 |
| **Income Medium High** | |
| **Feature** | **Values** |
| Weight | 0.299 |
| User | 0.278 |
| Temp | 0.160 |
| dia | 0.139 |
| sys | 0.125 |
| **Employment Students** | |
| **Feature** | **Values** |
| Weight | 0.293 |
| User | 0.222 |
| Temp | 0.145 |
| MAP | 0.144 |
| dia | 0.101 |
| **Employment Workers** | |
| **Feature** | **Values** |
| Weight | 0.252 |
| User | 0.237 |
| Temp | 0.154 |
| MAP | 0.137 |
| dia | 0.122 |
| **Age 18-24** | |
| **Feature** | **Values** |
| Weight | 0.295 |
| Temp | 0.207 |
| MAP | 0.151 |
| dia | 0.135 |
| sys | 0.127 |
| **Age 25-34** | |
| **Feature** | **Values** |
| Weight | 0.295 |
| Temp | 0.207 |
| MAP | 0.151 |
| dia | 0.135 |
| sys | 0.127 |
| **Age 35-44** | |
| **Feature** | **Values** |
| Weight | 0.231 |
| MAP | 0.173 |
| Temp | 0.168 |
| sys | 0.151 |
| dia | 0.146 |
| **Healthy** | |
| **Feature** | **Values** |
| Weight | 0.248 |
| Temp | 0.198 |
| MAP | 0.172 |
| sys | 0.139 |
| dia | 0.137 |

Table B12: Generalized Model - RF Feature Importance, DEmpatica

| **All** | |
| --- | --- |
| **Feature** | **Values** |
| User | 0.046 |
| Empatica_resp | 0.026 |
| Empatica_MSE3 | 0.021 |
| Empatica_ApEn | 0.020 |
| Empatica_FFT_RelativePower_VLF | 0.020 |
| Empatica_Max HR | 0.018 |
| Empatica_SD HR | 0.018 |
| Empatica_MSE4 | 0.018 |
| Empatica_FFT_LF | 0.018 |
| Empatica_D2 | 0.017 |
| **Gender - Male** | |
| **Feature** | **Values** |
| Empatica_MSE13 | 0.037 |
| Empatica_resp | 0.034 |
| Empatica_MSE3 | 0.028 |
| Empatica_MSE15 | 0.026 |
| Empatica_MSE2 | 0.024 |
| Empatica_MSE9 | 0.023 |
| Empatica_MSE11 | 0.021 |
| Empatica_FFT_LF | 0.020 |
| User | 0.019 |
| Empatica_MSE8 | 0.018 |
| **Gender - Female** | |
| **Feature** | **Values** |
| User | 0.056 |
| Empatica_NN50 | 0.025 |
| Empatica_MSE18 | 0.021 |
| Empatica_TINN | 0.019 |
| Empatica_DC | 0.019 |
| Empatica_SampEn | 0.019 |
| Empatica_SD1SD2 | 0.019 |
| Empatica_AR_RelativePower_VLF | 0.018 |
| Empatica_MSE19 | 0.018 |
| Empatica_resp | 0.017 |
| **Income - Low** | |
| **Feature** | **Values** |
| Empatica_DET | 0.024 |
| Empatica_DC | 0.024 |
| User | 0.024 |
| Empatica_REC | 0.021 |
| Empatica_MSE15 | 0.020 |
| Empatica_AR_AbsolutePower_VLF | 0.020 |
| Empatica_SampEn | 0.020 |
| Empatica_MSE3 | 0.020 |
| Empatica_MSE2 | 0.019 |
| Empatica_resp | 0.019 |
| **Income Medium High** | |
| **Feature** | **Values** |
| User | 0.0512 |
| Empatica_resp | 0.0252 |
| Empatica_DC | 0.0212 |
| Empatica_FFT_RelativePower_VLF | 0.0210 |
| Empatica_MSE12 | 0.0202 |
| Empatica_AR_LF | 0.0193 |
| Empatica_MSE3 | 0.0191 |
| Empatica_FFT_AbsolutePower_HF_log | 0.0189 |
| Empatica_FFT_AbsolutePower_HF | 0.0175 |
| Empatica_MSE14 | 0.0175 |
| **Employment Students** | |
| **Feature** | **Values** |
| User | 0.066 |
| Empatica_resp | 0.023 |
| Empatica_DC | 0.022 |
| Empatica_SampEn | 0.021 |
| Empatica_FFT_HF | 0.021 |
| Empatica_REC | 0.019 |
| Empatica_MSE3 | 0.019 |
| Empatica_FFT_RelativePower_LF | 0.019 |
| Empatica_MSE12 | 0.019 |
| Empatica_AR_AbsolutePower_VLF | 0.019 |
| **Employment Workers** | |
| **Feature** | **Values** |
| Empatica_MSE13 | 0.023 |
| User | 0.021 |
| Empatica_FFT_RelativePower_VLF | 0.020 |
| Empatica_MSE15 | 0.020 |
| Empatica_Min HR | 0.019 |
| Empatica_MSE3 | 0.019 |
| Empatica_ApEn | 0.018 |
| Empatica_MSE18 | 0.018 |
| Empatica_Mean RR | 0.018 |
| Empatica_resp | 0.018 |
| **Age 18-24** | |
| **Feature** | **Values** |
| Empatica_MSE4 | 0.028 |
| Empatica_MSE17 | 0.026 |
| Empatica_FFT_AbsolutePower_VLF_log | 0.024 |
| Empatica_AR_LF | 0.020 |
| Empatica_FFT_AbsolutePower_VLF | 0.020 |
| Empatica_FFT_LF | 0.019 |
| Empatica_MSE15 | 0.019 |
| Empatica_D2 | 0.019 |
| Empatica_MSE9 | 0.019 |
| Empatica_FFT_RelativePower_VLF | 0.019 |
| **Age 25-34** | |
| **Feature** | **Values** |
| User | 0.096 |
| Empatica_MSE6 | 0.024 |
| Empatica_AR_LF | 0.023 |
| Empatica_resp | 0.022 |
| Empatica_MSE8 | 0.021 |
| Empatica_MSE19 | 0.020 |
| Empatica_FFT_LFHF | 0.019 |
| Empatica_SampEn | 0.019 |
| Empatica_MSE18 | 0.018 |
| Empatica_MSE3 | 0.018 |
| **Age 35-44** | |
| **Feature** | **Values** |
| User | 0.032 |
| Empatica_MSE3 | 0.031 |
| Empatica_MSE15 | 0.028 |
| Empatica_FFT_HF | 0.027 |
| Empatica_resp | 0.025 |
| Empatica_MSE5 | 0.021 |
| Empatica_MSE16 | 0.020 |
| Empatica_MSE13 | 0.019 |
| Empatica_MSE12 | 0.018 |
| Empatica_FFT_AbsolutePower_VLF_log | 0.018 |
| **Healthy** | |
| **Feature** | **Values** |
| Empatica_resp | 0.030 |
| Empatica_MSE3 | 0.028 |
| Empatica_MSE2 | 0.022 |
| Empatica_MSE15 | 0.022 |
| User | 0.021 |
| Empatica_FFT_HF | 0.020 |
| Empatica_MSE4 | 0.019 |
| Empatica_MSE13 | 0.019 |
| Empatica_MSE17 | 0.018 |
| Empatica_MSE8 | 0.018 |

Table B13: Generalized Model - RF Feature Importance, SDA

| **All** | |
| --- | --- |
| **Feature** | **Values** |
| T+2 AW Consolidated Time During Awake | 0.023 |
| T+1 AW Consolidated Time During Awake | 0.023 |
| T+2 AW Number of Wake-Ups | 0.021 |
| AW Consolidated Time During Awake | 0.018 |
| AW Mean HR - Interval | 0.017 |
| AW Min HR - Interval | 0.017 |
| T-2 AW Mean HR | 0.017 |
| AW Total Time in Bed | 0.017 |
| T-2 AW Max HR | 0.017 |
| T-2 AW Total Time Asleep | 0.016 |
| **Gender - Male** | |
| **Feature** | **Values** |
| AW Min HR - Interval | 0.042 |
| AW Total Time in Bed | 0.024 |
| Short Term Min | 0.023 |
| User | 0.023 |
| T-2 AW Total Time Asleep | 0.022 |
| T+1 AW Consolidated Time During Awake | 0.021 |
| AW Mean HR - Interval | 0.020 |
| T-2 AW Total Time in Bed | 0.020 |
| ECG_AR_HF | 0.019 |
| T+2 AW Max HR | 0.019 |
| **Gender - Female** | |
| **Feature** | **Values** |
| T+1 AW Consolidated Time During Awake | 0.030 |
| T+2 AW Consolidated Time During Awake | 0.024 |
| User | 0.020 |
| AW Total Time in Bed | 0.019 |
| T+2 AW Number of Wake-Ups | 0.019 |
| AW Total Time Asleep | 0.018 |
| ECG_Stress Index | 0.017 |
| T-1 AW Total Time in Bed | 0.017 |
| AW Consolidated Time During Awake | 0.017 |
| AW Mean Steps | 0.016 |
| **Income - Low** | |
| **Feature** | **Values** |
| T+2 AW Consolidated Time During Awake | 0.034 |
| T+2 AW Number of Wake-Ups | 0.033 |
| T+1 AW Consolidated Time During Awake | 0.031 |
| AW Total Time in Bed | 0.021 |
| AW Consolidated Time During Awake | 0.021 |
| T+2 AW Total Time in Bed | 0.020 |
| T-1 Total Time in Bed | 0.020 |
| T-2 AW Number of Wake-Ups | 0.019 |
| T+1 AW Number of Wake-Ups | 0.019 |
| T-2 Consolidated Time During Awake | 0.018 |
| **Income Medium High** | |
| **Feature** | **Values** |
| User | 0.029 |
| AW Min HR | 0.023 |
| Short Term Min | 0.022 |
| T+2 AW Total Time in Bed | 0.021 |
| T-2 AW Mean HR | 0.021 |
| AW Min HR - Interval | 0.020 |
| T+2 AW Min HR | 0.020 |
| T+1 AW Consolidated Time During Awake | 0.020 |
| AW Mean HR - Interval | 0.020 |
| T+2 AW Number of Wake-Ups | 0.019 |
| **Employment Students** | |
| **Feature** | **Values** |
| T+1 AW Consolidated Time During Awake | 0.035 |
| T+2 AW Number of Wake-Ups | 0.035 |
| T+2 AW Consolidated Time During Awake | 0.029 |
| T+1 AW Number of Wake-Ups | 0.022 |
| AW Consolidated Time During Awake | 0.021 |
| AW Number of Wake-Ups | 0.017 |
| T+2 AW Total Time in Bed | 0.017 |
| T-2 AW Number of Wake-Ups | 0.017 |
| ECG_FFT_RelativePower_LF | 0.016 |
| ECG_FFT_LFHF | 0.016 |
| **Employment Workers** | |
| **Feature** | **Values** |
| User | 0.028 |
| AW Min HR - Interval | 0.024 |
| AW Total Time in Bed | 0.021 |
| AW Mean HR - Interval | 0.020 |
| T+1 AW Max HR | 0.019 |
| Short Term Min | 0.019 |
| T+2 AW Total Time in Bed | 0.019 |
| T-2 AW Mean HR | 0.019 |
| AW Total Time Asleep | 0.018 |
| T+2 AW Mean HR | 0.017 |
| **Age 18-24** | |
| **Feature** | **Values** |
| T+2 AW Number of Wake-Ups | 0.024 |
| T+1 AW Consolidated Time During Awake | 0.024 |
| T+2 AW Consolidated Time During Awake | 0.023 |
| AW Total Time in Bed | 0.021 |
| AW Mean Steps | 0.020 |
| AW % of Time Asleep While In Bed | 0.019 |
| T+2 AW % of Time Asleep While In Bed | 0.019 |
| T+2 AW Total Time in Bed | 0.018 |
| AW Consolidated Time During Awake | 0.017 |
| T-2 AW Min HR | 0.017 |
| **Age 25-34** | |
| **Feature** | **Values** |
| T+2 AW Consolidated Time During Awake | 0.027 |
| T+2 AW Number of Wake-Ups | 0.026 |
| T-2 AW Total Time Asleep | 0.025 |
| T-2 AW Total Time in Bed | 0.024 |
| AW Consolidated Time During Awake | 0.022 |
| T+2 AW Total Time in Bed | 0.022 |
| AW Total Time in Bed | 0.022 |
| AW Mean HR - Interval | 0.021 |
| HRV-1 | 0.021 |
| T-1 AW Total Time in Bed | 0.020 |
| **Age 35-44** | |
| **Feature** | **Values** |
| AW Min HR | 0.030 |
| T+1 AW Mean HR | 0.026 |
| T-2 AW % of Time Asleep While In Bed | 0.024 |
| AW Min HR - Interval | 0.024 |
| Short Term Min | 0.022 |
| AW Mean HR - Interval | 0.020 |
| AW Min Steps | 0.019 |
| T+2 AW Mean HR | 0.019 |
| AW Mean Steps | 0.018 |
| T-1 AW Min HR | 0.018 |
| **Healthy** | |
| **Feature** | **Values** |
| T+2 AW Consolidated Time During Awake | 0.021 |
| T-2 AW Max HR | 0.020 |
| T+1 AW Consolidated Time During Awake | 0.020 |
| AW Mean Steps | 0.019 |
| T+1 AW Max HR | 0.017 |
| AW Min HR - Interval | 0.017 |
| AW Total Time Asleep | 0.017 |
| T-2 AW Total Time Asleep | 0.017 |
| AW Max Steps | 0.017 |
| T+2 AW Number of Wake-Ups | 0.016 |

Table B14: Generalized Model - RF Feature Importance, SDAW

| **All** | |
| --- | --- |
| **Feature** | **Values** |
| T+2 AW Number of Wake-Ups | 0.020 |
| T+1 AW Consolidated Time During Awake | 0.019 |
| Weight | 0.017 |
| T+2 AW Consolidated Time During Awake | 0.017 |
| Temp | 0.015 |
| AW Total Time in Bed | 0.014 |
| MAP | 0.014 |
| T+2 AW Min HR | 0.014 |
| AW Min HR - Interval | 0.013 |
| T+1 AW Number of Wake-Ups | 0.013 |
| **Gender - Male** | |
| **Feature** | **Values** |
| T+1 AW Max HR | 0.030 |
| T-2 AW Total Time Asleep | 0.024 |
| T-2 AW Max HR | 0.021 |
| AW Max HR | 0.020 |
| ECG_FFT_RelativePower_HF | 0.018 |
| Weight | 0.018 |
| MAP | 0.018 |
| T-1 AW Max HR | 0.017 |
| sys | 0.016 |
| Short Term Mean | 0.016 |
| **Gender - Female** | |
| **Feature** | **Values** |
| T+1 AW Consolidated Time During Awake | 0.022 |
| AW Total Time in Bed | 0.018 |
| Weight | 0.017 |
| Temp | 0.017 |
| T+2 AW Number of Wake-Ups | 0.015 |
| T+2 AW Consolidated Time During Awake | 0.015 |
| ECG_Stress Index | 0.014 |
| AW Min HR - Interval | 0.014 |
| AW Min HR | 0.014 |
| ECG_SNS_Index | 0.013 |
| **Income - Low** | |
| **Feature** | **Values** |
| T+1 AW Consolidated Time During Awake | 0.033 |
| T+2 AW Consolidated Time During Awake | 0.028 |
| T+1 AW Number of Wake-Ups | 0.027 |
| T+2 AW Number of Wake-Ups | 0.024 |
| AW Consolidated Time During Awake | 0.016 |
| T-2 AW Number of Wake-Ups | 0.015 |
| T+2 AW % of Time Asleep While In Bed | 0.014 |
| T+2 Time Spent in REM Stage | 0.014 |
| AW Min HR - Interval | 0.014 |
| T-2 Time Spent in Light Stage | 0.013 |
| **Income Medium High** | |
| **Feature** | **Values** |
| AW Min HR | 0.027 |
| T+2 AW Min HR | 0.018 |
| T-1 AW Total Time in Bed | 0.017 |
| T-2 AW Min HR | 0.017 |
| AW Total Time in Bed | 0.016 |
| Temp | 0.016 |
| T+1 AW Consolidated Time During Awake | 0.016 |
| T-2 AW Mean HR | 0.015 |
| AW Consolidated Time During Awake | 0.014 |
| ECG_AR_AbsolutePower_HF | 0.014 |
| **Employment Students** | |
| **Feature** | **Values** |
| T+2 AW Consolidated Time During Awake | 0.031 |
| T+2 AW Number of Wake-Ups | 0.027 |
| T+2 Time Spent in REM Stage | 0.026 |
| T+1 AW Consolidated Time During Awake | 0.025 |
| T+1 AW Number of Wake-Ups | 0.023 |
| T+2 AW Min HR | 0.019 |
| Time Spent in REM Stage | 0.018 |
| AW Number of Wake-Ups | 0.017 |
| Weight | 0.013 |
| T-2 AW Min HR | 0.012 |
| **Employment Workers** | |
| **Feature** | **Values** |
| AW Total Time in Bed | 0.018 |
| AW Mean HR - Interval | 0.017 |
| AW Min HR | 0.017 |
| Temp | 0.016 |
| AW Mean Steps | 0.015 |
| AW Min HR - Interval | 0.015 |
| T-1 AW Total Time in Bed | 0.014 |
| Time Spent in REM Stage | 0.014 |
| HRV-1 | 0.013 |
| sys | 0.013 |
| **Age 18-24** | |
| **Feature** | **Values** |
| Weight | 0.025 |
| T+2 Time Spent in REM Stage | 0.025 |
| T+2 AW Consolidated Time During Awake | 0.024 |
| T+2 AW Min HR | 0.023 |
| T+1 AW Min HR | 0.019 |
| T+1 AW Consolidated Time During Awake | 0.016 |
| Time Spent in REM Stage | 0.016 |
| AW Number of Wake-Ups | 0.015 |
| ECG_FFT_RelativePower_LF | 0.015 |
| T-1 AW Min HR | 0.015 |
| **Age 25-34** | |
| **Feature** | **Values** |
| T+2 AW Consolidated Time During Awake | 0.030 |
| T+2 AW Number of Wake-Ups | 0.028 |
| Weight | 0.022 |
| T+2 AW Mean HR | 0.018 |
| T+1 AW Min HR | 0.018 |
| T+2 AW Min HR | 0.018 |
| T-2 % of Time Asleep While In Bed | 0.017 |
| T+1 AW Consolidated Time During Awake | 0.017 |
| % of Time Asleep While In Bed | 0.016 |
| AW Number of Wake-Ups | 0.015 |
| **Age 35-44** | |
| **Feature** | **Values** |
| T+2 AW Mean HR | 0.018 |
| T+1 AW Mean HR | 0.018 |
| T+2 Time Spent in Light Stage | 0.018 |
| T-1 Withings Total Time Asleep | 0.016 |
| AW Max HR - Interval | 0.016 |
| Temp | 0.015 |
| T-2 Time Spent in Deep Stage | 0.015 |
| HRV-1 | 0.015 |
| AW Mean HR - Interval | 0.015 |
| AW Min HR | 0.014 |
| **Healthy** | |
| **Feature** | **Values** |
| T+2 AW Number of Wake-Ups | 0.020 |
| AW Total Time in Bed | 0.019 |
| Weight | 0.018 |
| T+1 AW Consolidated Time During Awake | 0.018 |
| T+2 AW Consolidated Time During Awake | 0.017 |
| User | 0.016 |
| T-2 AW Max HR | 0.015 |
| T-2 AW Total Time in Bed | 0.015 |
| T-2 AW Min HR | 0.014 |
| T-2 Time Spent in REM Stage | 0.014 |

Table B15: Generalized Model - RF Feature Importance, SDW

| **All** | |
| --- | --- |
| **Feature** | **Values** |
| Weight | 0.089 |
| Temp | 0.082 |
| MAP | 0.068 |
| dia | 0.063 |
| sys | 0.062 |
| T-2 Time Spent in REM Stage | 0.034 |
| T+2 Time Spent in REM Stage | 0.031 |
| Withings Total Time Asleep | 0.030 |
| T+1 Withings Total Time Asleep | 0.029 |
| Time Spent in REM Stage | 0.029 |
| **Gender - Male** | |
| **Feature** | **Values** |
| T-1 Withings Total Time Asleep | 0.058 |
| T+2 Withings Total Time Asleep | 0.056 |
| T-2 Withings Total Time Asleep | 0.055 |
| Temp | 0.049 |
| MAP | 0.048 |
| T+1 Withings Total Time Asleep | 0.045 |
| sys | 0.044 |
| T-2 Time Spent in Light Stage | 0.044 |
| T+2 Time Spent in Light Stage | 0.043 |
| Withings Total Time Asleep | 0.043 |
| **Gender - Female** | |
| **Feature** | **Values** |
| Weight | 0.067 |
| User | 0.053 |
| T+1 Withings Total Time Asleep | 0.053 |
| Temp | 0.044 |
| T+2 Withings Total Time Asleep | 0.042 |
| Withings Total Time Asleep | 0.042 |
| T-2 Time Spent in REM Stage | 0.039 |
| sys | 0.037 |
| T+2 Time Spent in REM Stage | 0.036 |
| T-1 Withings Total Time Asleep | 0.035 |
| **Income - Low** | |
| **Feature** | **Values** |
| Weight | 0.066 |
| Temp | 0.062 |
| MAP | 0.055 |
| sys | 0.050 |
| dia | 0.049 |
| T+2 Time Spent in REM Stage | 0.043 |
| Time Spent in Light Stage | 0.040 |
| T-2 Time Spent in REM Stage | 0.039 |
| Time Spent in REM Stage | 0.035 |
| Total Time In Bed | 0.034 |
| **Income Medium High** | |
| **Feature** | **Values** |
| Temp | 0.086 |
| Weight | 0.071 |
| MAP | 0.065 |
| sys | 0.060 |
| dia | 0.060 |
| User | 0.052 |
| Time Spent in REM Stage | 0.042 |
| T+1 Withings Total Time Asleep | 0.033 |
| T+2 Time Spent in Light Stage | 0.028 |
| T+2 Withings Total Time Asleep | 0.028 |
| **Employment Students** | |
| **Feature** | **Values** |
| T+2 Time Spent in REM Stage | 0.070 |
| Weight | 0.067 |
| Time Spent in REM Stage | 0.055 |
| Temp | 0.053 |
| MAP | 0.047 |
| T-2 Time Spent in REM Stage | 0.046 |
| User | 0.040 |
| sys | 0.040 |
| dia | 0.039 |
| T+2 % of Time Asleep While In Bed | 0.036 |
| **Employment Workers** | |
| **Feature** | **Values** |
| Temp | 0.088 |
| Weight | 0.071 |
| MAP | 0.068 |
| dia | 0.067 |
| sys | 0.064 |
| T+1 Withings Total Time Asleep | 0.035 |
| User | 0.034 |
| T-1 Withings Total Time Asleep | 0.032 |
| T-2 Time Spent in REM Stage | 0.029 |
| T+2 Withings Total Time Asleep | 0.029 |
| **Age 18-24** | |
| **Feature** | **Values** |
| Weight | 0.098 |
| Temp | 0.078 |
| MAP | 0.072 |
| sys | 0.066 |
| dia | 0.065 |
| T+2 Time Spent in REM Stage | 0.049 |
| Time Spent in REM Stage | 0.044 |
| % of Time Asleep While In Bed | 0.033 |
| T+2 Withings Total Time Asleep | 0.031 |
| T+2 % of Time Asleep While In Bed | 0.030 |
| **Age 25-34** | |
| **Feature** | **Values** |
| Weight | 0.103 |
| T-2 % of Time Asleep While In Bed | 0.057 |
| T+2 % of Time Asleep While In Bed | 0.050 |
| T-2 Time Spent in Deep Stage | 0.049 |
| % of Time Asleep While In Bed | 0.049 |
| User | 0.046 |
| Time Spent in REM Stage | 0.046 |
| Temp | 0.038 |
| MAP | 0.038 |
| T+2 Time Spent in Deep Stage | 0.034 |
| **Age 35-44** | |
| **Feature** | **Values** |
| T+2 Withings Total Time Asleep | 0.069 |
| T+2 Total Time In Bed | 0.064 |
| T-1 Withings Total Time Asleep | 0.056 |
| T+2 Time Spent in Light Stage | 0.047 |
| T-2 Time Spent in REM Stage | 0.046 |
| Total Time In Bed | 0.045 |
| User | 0.045 |
| T-2 Total Time In Bed | 0.041 |
| Weight | 0.040 |
| T-2 Withings Total Time Asleep | 0.039 |
| **Healthy** | |
| **Feature** | **Values** |
| Weight | 0.080 |
| Temp | 0.049 |
| MAP | 0.047 |
| sys | 0.045 |
| T-2 Time Spent in REM Stage | 0.045 |
| T+1 Withings Total Time Asleep | 0.041 |
| dia | 0.040 |
| Time Spent in Light Stage | 0.039 |
| Time Spent in Deep Stage | 0.037 |
| T+2 Withings Total Time Asleep | 0.036 |

Table B16: Generalized Model - RF Feature Importance, SDS

| **All** | |
| --- | --- |
| **Feature** | **Values** |
| T+2 AW Number of Wake-Ups | 0.049 |
| T+1 AW Consolidated Time During Awake | 0.047 |
| User | 0.034 |
| T+2 AW Consolidated Time During Awake | 0.033 |
| AW Total Time in Bed | 0.033 |
| T-2 AW Min HR | 0.029 |
| T+2 AW Min HR | 0.028 |
| T+1 AW Number of Wake-Ups | 0.028 |
| AW Min HR | 0.026 |
| T-2 Time Spent in REM Stage | 0.024 |
| **Gender - Male** | |
| **Feature** | **Values** |
| T+1 AW Max HR | 0.039 |
| T-2 AW Max HR | 0.038 |
| T+2 AW Consolidated Time During Awake | 0.034 |
| AW Max HR | 0.033 |
| T-2 AW Total Time in Bed | 0.033 |
| T-1 AW Max HR | 0.028 |
| T-2 AW Total Time Asleep | 0.028 |
| T+2 AW % of Time Asleep While In Bed | 0.026 |
| T-2 Time Spent in REM Stage | 0.025 |
| T+2 % of Time Asleep While In Bed | 0.022 |
| **Gender - Female** | |
| **Feature** | **Values** |
| AW Total Time in Bed | 0.039 |
| T+1 AW Consolidated Time During Awake | 0.037 |
| User | 0.027 |
| T+2 AW Total Time Asleep | 0.026 |
| AW Min HR | 0.025 |
| Time Spent in Light Stage | 0.024 |
| T+2 Time Spent in REM Stage | 0.023 |
| AW Consolidated Time During Awake | 0.022 |
| T-2 AW Min HR | 0.022 |
| T-2 Time Spent in Deep Stage | 0.021 |
| **Income - Low** | |
| **Feature** | **Values** |
| T+1 AW Consolidated Time During Awake | 0.043 |
| T+2 AW Consolidated Time During Awake | 0.036 |
| T+2 AW Number of Wake-Ups | 0.034 |
| Time Spent in Light Stage | 0.028 |
| T-2 W Consolidated Time During Awake | 0.027 |
| T+1 AW Number of Wake-Ups | 0.027 |
| T+2 AW % of Time Asleep While In Bed | 0.026 |
| T-2 AW Number of Wake-Ups | 0.022 |
| T-1 AW Mean HR | 0.021 |
| AW Consolidated Time During Awake | 0.021 |
| **Income Medium High** | |
| **Feature** | **Values** |
| AW Min HR | 0.047 |
| T-1 AW Total Time in Bed | 0.032 |
| AW Total Time in Bed | 0.028 |
| T+2 AW Min HR | 0.028 |
| T+1 AW Min HR | 0.026 |
| T-2 AW Min HR | 0.024 |
| T-2 AW Mean HR | 0.024 |
| AW Number of Wake-Ups | 0.023 |
| AW Consolidated Time During Awake | 0.022 |
| T+1 AW Consolidated Time During Awake | 0.022 |
| **Employment Students** | |
| **Feature** | **Values** |
| T+1 AW Consolidated Time During Awake | 0.047 |
| T+2 AW Number of Wake-Ups | 0.041 |
| T+2 Time Spent in REM Stage | 0.040 |
| T+2 AW Min HR | 0.036 |
| T+1 AW Number of Wake-Ups | 0.035 |
| T+2 AW Consolidated Time During Awake | 0.030 |
| Time Spent in REM Stage | 0.029 |
| AW Number of Wake-Ups | 0.022 |
| T-2 Time Spent in REM Stage | 0.022 |
| User | 0.021 |
| **Employment Workers** | |
| **Feature** | **Values** |
| AW Min HR | 0.040 |
| AW Total Time in Bed | 0.040 |
| T-1 AW Total Time in Bed | 0.036 |
| User | 0.030 |
| Time Spent in REM Stage | 0.028 |
| AW Max HR | 0.027 |
| T+2 AW Max HR | 0.024 |
| AW Consolidated Time During Awake | 0.024 |
| T+2 AW Total Time in Bed | 0.024 |
| AW Mean HR | 0.022 |
| **Age 18-24** | |
| **Feature** | **Values** |
| Time Spent in REM Stage | 0.043 |
| T+2 Time Spent in REM Stage | 0.042 |
| T+1 AW Min HR | 0.040 |
| T-2 AW Min HR | 0.040 |
| T+2 AW Min HR | 0.038 |
| T+1 AW Consolidated Time During Awake | 0.038 |
| T-1 AW Min HR | 0.035 |
| AW Min HR | 0.032 |
| T-2 AW Mean HR | 0.029 |
| T+2 AW Total Time in Bed | 0.026 |
| **Age 25-34** | |
| **Feature** | **Values** |
| T+2 AW Number of Wake-Ups | 0.058 |
| T+2 AW Consolidated Time During Awake | 0.047 |
| T+2 AW Mean HR | 0.042 |
| T+1 AW Min HR | 0.034 |
| T-2 % of Time Asleep While In Bed | 0.033 |
| AW Number of Wake-Ups | 0.031 |
| T+2 AW Min HR | 0.030 |
| T+1 AW Mean HR | 0.027 |
| T+1 AW Consolidated Time During Awake | 0.027 |
| AW Mean HR | 0.024 |
| **Age 35-44** | |
| **Feature** | **Values** |
| T-1 Withings Total Time Asleep | 0.042 |
| T+1 AW Mean HR | 0.031 |
| T-2 Time Spent in REM Stage | 0.030 |
| T+1 Withings Total Time Asleep | 0.028 |
| T+2 AW Mean HR | 0.027 |
| T+2 Time Spent in Light Stage | 0.026 |
| T+2 AW Max HR | 0.025 |
| AW Min HR | 0.025 |
| T-2 AW % of Time Asleep While In Bed | 0.024 |
| T-2 Time Spent in Deep Stage | 0.023 |
| **Healthy** | |
| **Feature** | **Values** |
| T+2 AW Consolidated Time During Awake | 0.051 |
| T-2 AW Total Time in Bed | 0.044 |
| T+1 AW Consolidated Time During Awake | 0.043 |
| T+2 AW Total Time in Bed | 0.036 |
| User | 0.035 |
| T+2 AW Number of Wake-Ups | 0.032 |
| T-2 AW Max HR | 0.029 |
| T-1 AW Total Time in Bed | 0.025 |
| T+2 Time Spent in REM Stage | 0.024 |
| T+1 AW Min HR | 0.021 |

**References for Table B6**

[1] A. Muaremi, B. Arnrich, and G. Tröster, “Towards Measuring Stress with Smartphones and Wearable Devices During Workday and Sleep,” *Bionanoscience*, vol. 3, no. 2, pp. 172–183, 2013, doi: 10.1007/s12668-013-0089-2.

[2] C. Setz, B. Arnrich, J. Schumm, R. La Marca, G. Tr, and U. Ehlert, “Discriminating stress from cognitive load using a wearable EDA device.,” *Technology*, vol. 14, no. 2, pp. 410–417, 2010, doi: 10.1109/TITB.2009.2036164.

[3] C. W. Jin, Z. Oravecz, and A. Osotsi, “Predicting stress in teens from wearable device data using machine learning methods,” *medRxiv*, 2020, doi: 10.1101/2020.11.26.20223784.

[4] J. Choi, B. Ahmed, and R. Gutierrez-Osuna, “Development and evaluation of an ambulatory stress monitor based on wearable sensors,” *IEEE Trans. Inf. Technol. Biomed.*, vol. 16, no. 2, pp. 279–286, 2012, doi: 10.1109/TITB.2011.2169804.

[5] Y. S. Can, D. Gokay, D. R. Kılıç, D. Ekiz, N. Chalabianloo, and C. Ersoy, “How laboratory experiments can be exploited for monitoring stress in the wild: A bridge between laboratory and daily life,” *Sensors (Switzerland)*, vol. 20, no. 3, 2020, doi: 10.3390/s20030838.

[6] A. Muaremi, A. Bexheti, F. Gravenhorst, B. Arnrich, and G. Troster, “Monitoring the impact of stress on the sleep patterns of pilgrims using wearable sensors,” *2014 IEEE-EMBS Int. Conf. Biomed. Heal. Informatics, BHI 2014*, pp. 185–188, 2014, doi: 10.1109/BHI.2014.6864335.

[7] A. O. Akmandor and N. K. Jha, “Keep the Stress Away with SoDA: Stress Detection and Alleviation System,” *IEEE Trans. Multi-Scale Comput. Syst.*, vol. 3, no. 4, pp. 269–282, 2017, doi: 10.1109/TMSCS.2017.2703613.

[8] K. Hovsepian, M. Al’absi, E. Ertin, T. Kamarck, M. Nakajima, and S. Kumar, “CStress: Towards a gold standard for continuous stress assessment in the mobile environment,” in *UbiComp 2015 - Proceedings of the 2015 ACM International Joint Conference on Pervasive and Ubiquitous Computing*, 2015, pp. 493–504, doi: 10.1145/2750858.2807526.

[9] M. Gjoreski, H. Gjoreski, M. Luštrek, and M. Gams, “Continuous stress detection using a wrist device - in laboratory and real life,” *UbiComp 2016 Adjun. - Proc. 2016 ACM Int. Jt. Conf. Pervasive Ubiquitous Comput.*, pp. 1185–1193, 2016, doi: 10.1145/2968219.2968306.

[10] C. Y. Liao, R. C. Chen, and S. K. Tai, “Emotion stress detection using EEG signal and deep learning technologies,” *Proc. 4th IEEE Int. Conf. Appl. Syst. Innov. 2018, ICASI 2018*, no. 2, pp. 90–93, 2018, doi: 10.1109/ICASI.2018.8394414.

[11] A. Sano and R. W. Picard, “Stress recognition using wearable sensors and mobile phones,” *Proc. - 2013 Hum. Assoc. Conf. Affect. Comput. Intell. Interact. ACII 2013*, pp. 671–676, 2013, doi: 10.1109/ACII.2013.117.

[12] E. Garcia-Ceja, V. Osmani, and O. Mayora, “Automatic Stress Detection in Working Environments from Smartphones’ Accelerometer Data: A First Step,” *IEEE J. Biomed. Heal. Informatics*, vol. 20, no. 4, pp. 1053–1060, 2016, doi: 10.1109/JBHI.2015.2446195.

[13] Y. S. Can, N. Chalabianloo, D. Ekiz, and C. Ersoy, “Continuous stress detection using wearable sensors in real life: Algorithmic programming contest case study,” *Sensors (Switzerland)*, vol. 19, no. 8, 2019, doi: 10.3390/s19081849.

[14] R. Castaldo, L. Montesions, P. Melillo, S. Massaro, and L. Pecchia, “To What Extent Can we Shorten HRV Analysis in Wearable Sensing? A Case Study on Mental Stress Detection,” 2017, doi: 10.1007/978-981-10-5122-7.

[15] V. Vanitha and P. Krishnan, “Real time stress detection system based on EEG signals,” *Biomed. Res.*, vol. 2016, no. Special Issue 2, pp. S271–S275, 2016.

[16] V. Sandulescu, S. Andrews, D. Ellis, N. Bellotto, and O. M. Mozos, “Stress detection using wearable physiological sensors,” *Lect. Notes Comput. Sci. (including Subser. Lect. Notes Artif. Intell. Lect. Notes Bioinformatics)*, vol. 9107, pp. 526–532, 2015, doi: 10.1007/978-3-319-18914-7_55.
