# Supplementary material for: Application of a mobile health data platform for public health surveillance: A case study in stress monitoring and prediction
Source: Digit Health. 2024 Jun 8;10:20552076241249931. doi: 10.1177/20552076241249931 (PMC11394344; doi:10.1177/20552076241249931)
Supplement: sj-docx-3-dhj-10.1177_20552076241249931 - Supplemental material for Application of a mobile health data platform for public health surveillance: A case study in stress monitoring and prediction [file sj-docx-3-dhj-10.1177_20552076241249931.docx]

## Supplementary Material

## Appendix 3 – User Manual

**Get Started**

Welcome and thank you for participating in our study and contributing to our research on the use of wearable devices to improve population health and stress detection. We ask that you please take a moment to read these instructions to ensure you know how to properly install the devices and apps.

**Study Package**

You should have received a package with the devices below and 3 documents: **User Manual – Get Started, User Manual – Data Collection Schedule,** and **User Manual – Data Collection Protocol.**

The contents of the documents are as follows:

**User Manual – Get Started:** Please read this document first in order to properly set up the devices.

**User Manual – Data Collection Schedule:** Please read this document to understand the schedule for data collection.

**User Manual – Data Collection Protocol:** Please read this document to understand the data collection protocol for each device.

**Devices of the Study**

For this study, you should have received:

1. **iPhone** (iOS 14.1 or higher) and **charging cable**


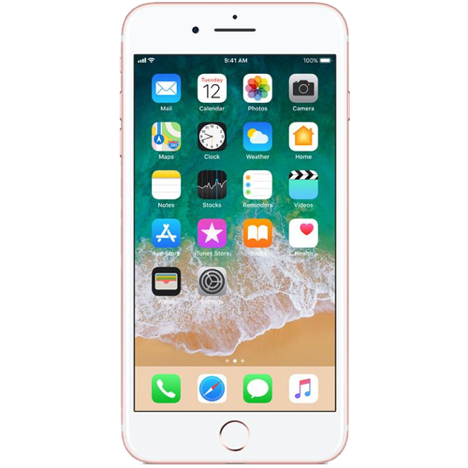

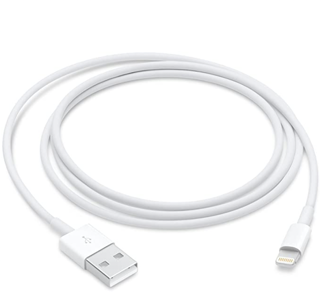


1. **Apple Watch** (Series 4 or higher, watchOS 5.1 or higher), **small size band** and **charging cable**


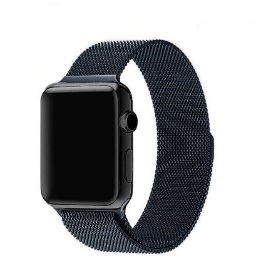

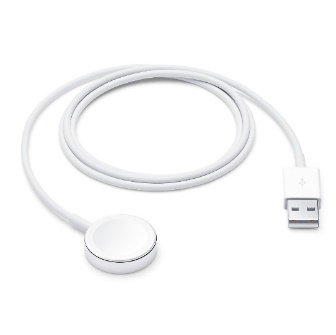


1. **Withings Sleep** and **charging cable**

**
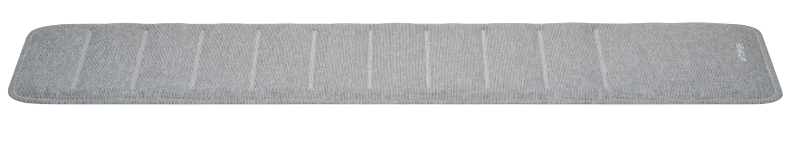
**

1. **Withings BPM Connect** and **charging cable**


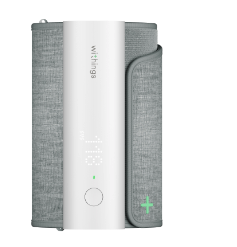


1. **Withings Wireless Scale**


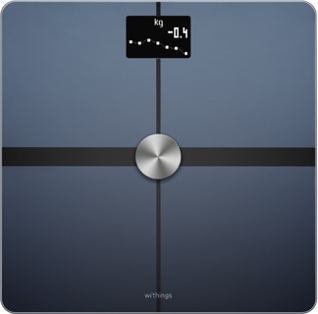


1. **Withings Termos**


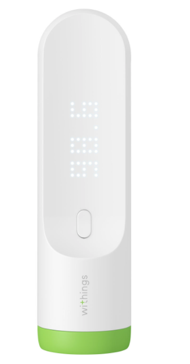


1. **Empatica E4** and **charging cable**


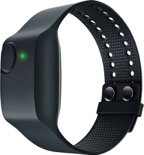


**Setting up the devices**

1. **iPhone – Get Started – Password of iPhone: 000000**

- Open the Health app
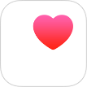
**.**
- On the Browse tap, please **select Body Measurements.**


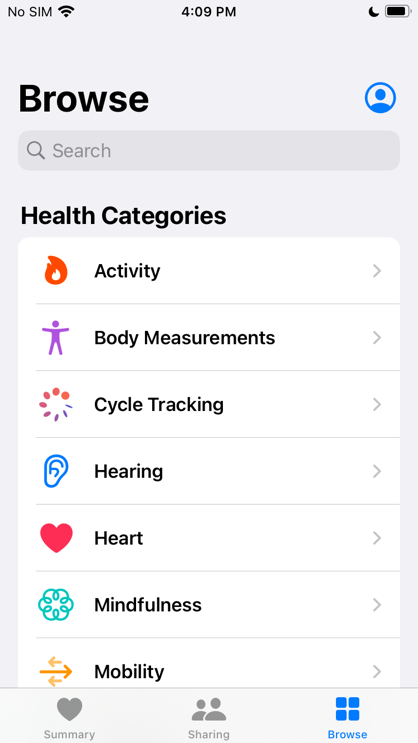


- Click on **Height.**
- On the top right corner, select **Add Data.**
- Please include your Height.
- On the Browse tap, please **select Sleep.**


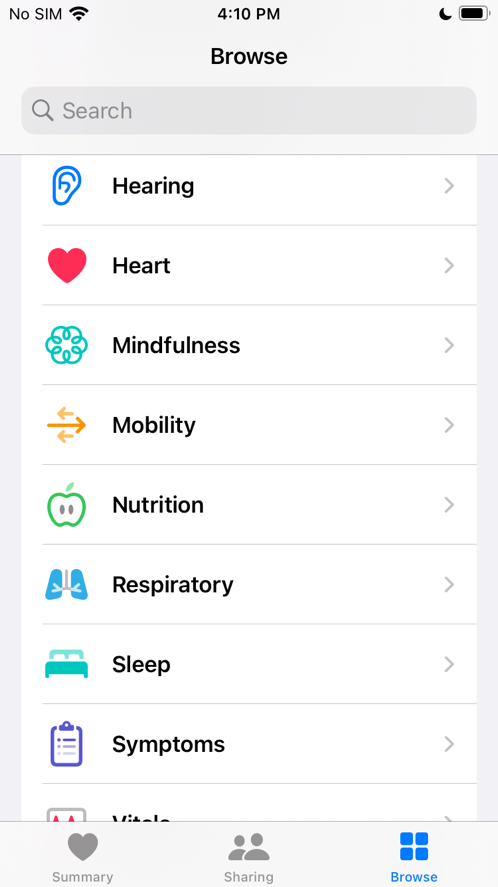


- In **Your Schedule,** click **Edit** and insert your sleep schedule. Please include the most approximate estimate of the times you generally go to sleep and wake up.


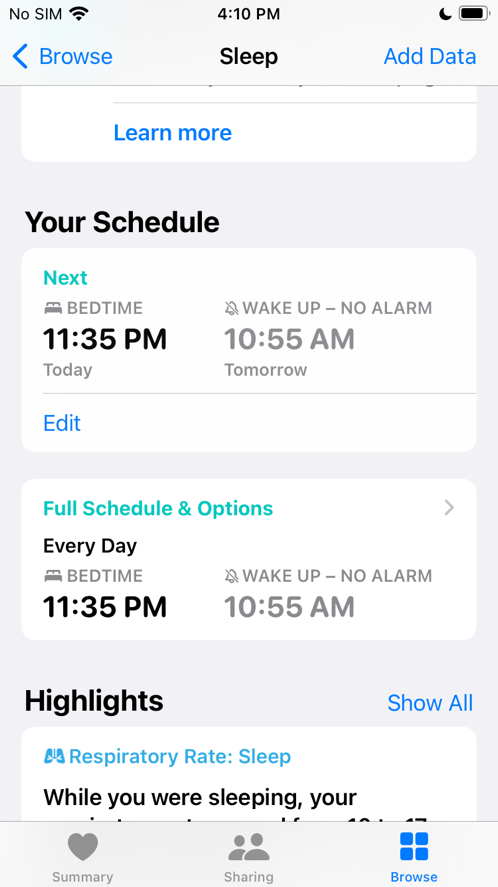


1. **Apple Watch – Get Started**

**Placement** The Apple Watch band should fit closely but comfortably on the top of your wrist. Please adjust the band accordingly, not too tight or too loose and with room for the skin to breathe. You may tighten Apple Watch for workouts if necessary, and loosen the band when the workout is done. Please use the Apple Watch in your dominant wrist and adjust it so that the Digital Crown on the side of the Watch is nearest to the top of your wrist.


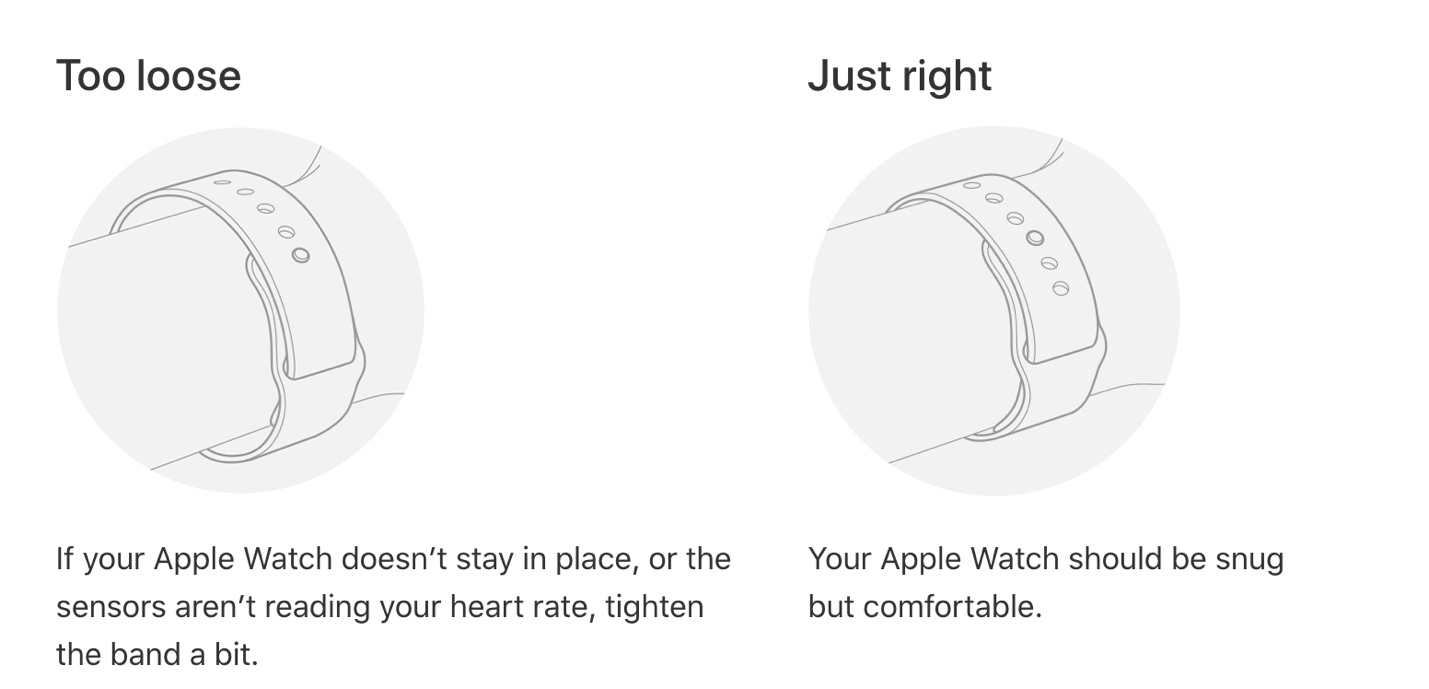


**In case band is too big** The package also contains a smaller size band for the bottom of the Apple Watch. To switch bands, please hold down the band release button and slide the band across to remove it.

**
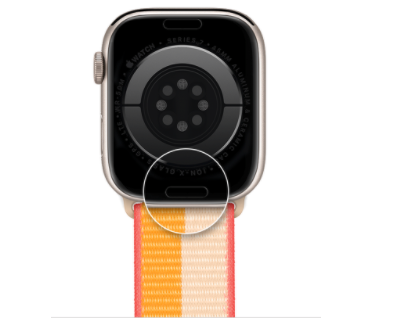
**

Adapted from: <https://support.apple.com/en-us/HT204818>

**Select Crown Orientation and Dominant Hand** The Digital Crown should be nearest to the top of your wrist. If necessary**,** please adjust your orientation as follows: open the Settings app
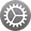
, then go to General > Orientation. To change the settings in the Apple Watch app on iPhone, tap My Watch, then go to General > Watch Orientation.


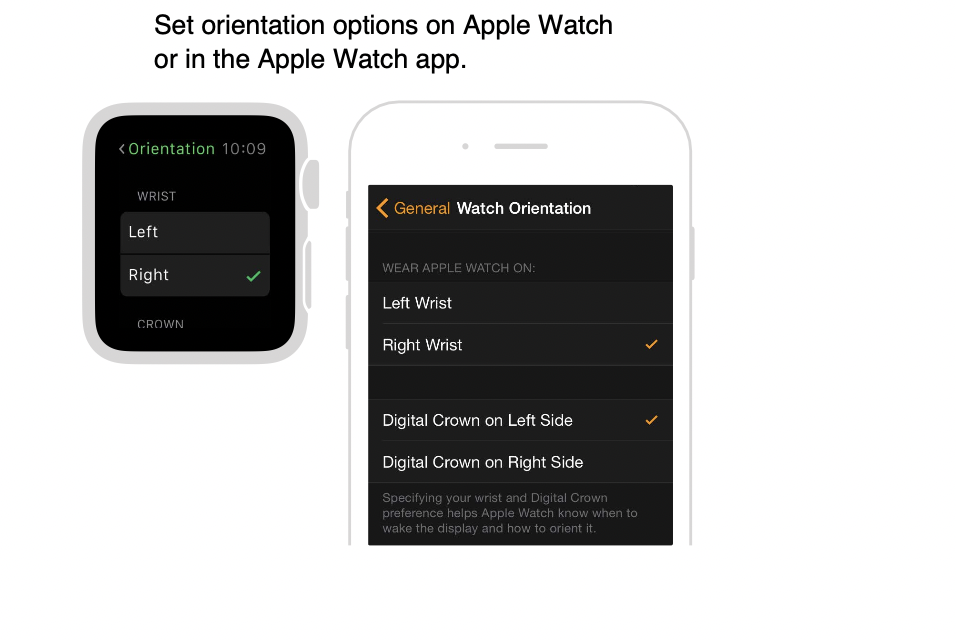


**Charging:** Please follow the instructions below to charge your Apple Watch:

1.Plug in the charging cable into the USB port on your computer or the USB wall charger provided.

2.Place the concave end of the charging cable on the back of your Apple Watch. The concave end of the charging cable magnetically snaps to the back of your Apple Watch and aligns it properly.

3.You will hear a chime when charging begins (unless your Apple Watch is in silent mode) and see a green charging symbol on the watch face.

4.Charging fully takes about two to three hours. While the watch charges, you can tap it to check the battery level. A fully charged watch shows the green charging symbol encircled by a green circle on the watch face.

Your fully charged Apple Watch has battery life of up to 18 hours.


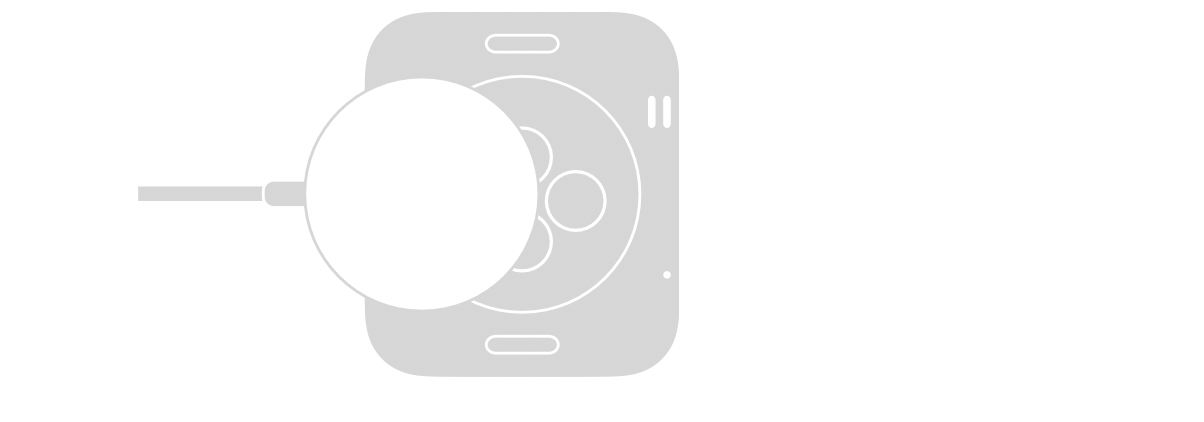


Adapted from: <https://support.apple.com/en-ca/guide/watch/welcome/watchos>

1. **Health Mate – Get Started**

1.Open the Health Mate ^
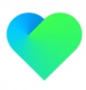
^ app.

2. On the tab at the top, click the **+** sign at the top.


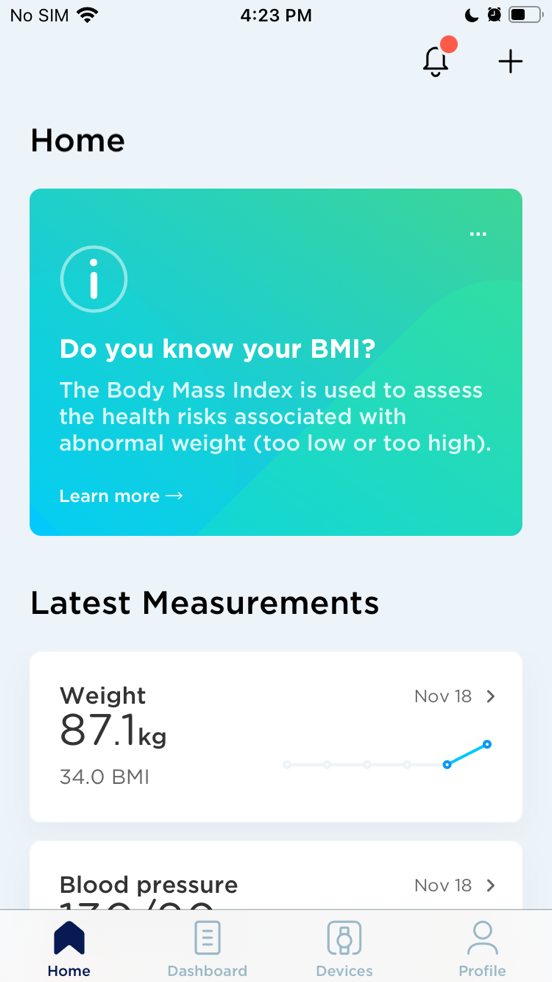


3. Select **Weight** and include an estimate of weight. This information is necessary for the scale to identify you as the user.


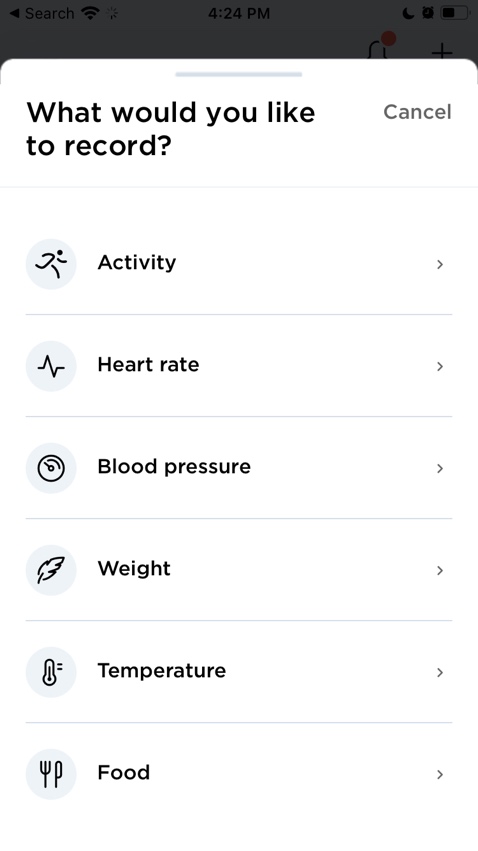


1. **Withings Sleep – Get Started**
2. Open the Health Mate ^
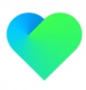
^ app.
3. Tap the Devices tab, scroll to the bottom and select Install a Device.


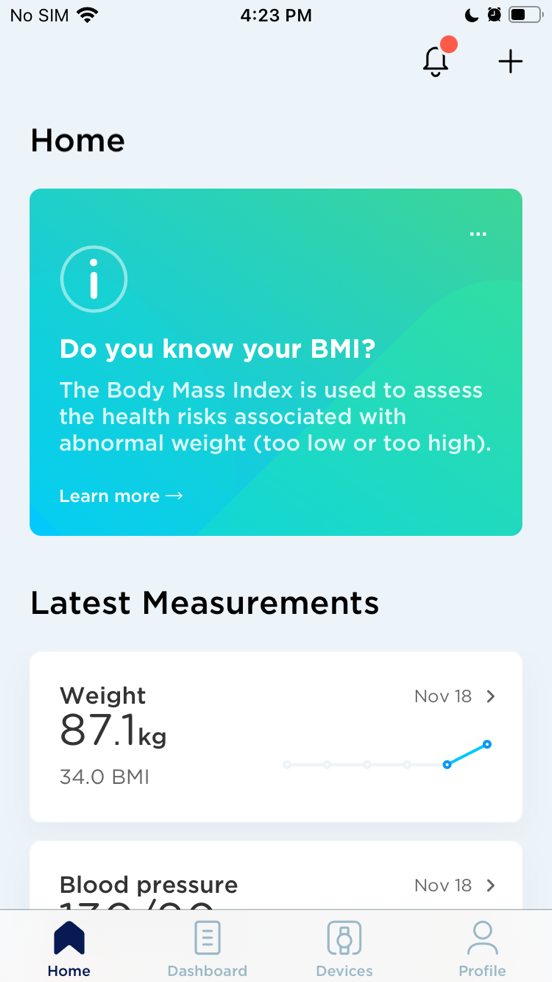


1. Tap Sleep Sensors


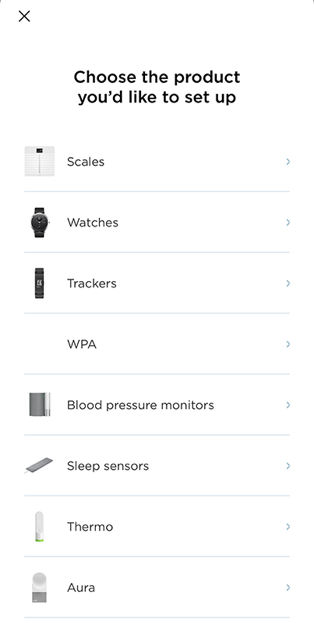


1. Tap Sleep


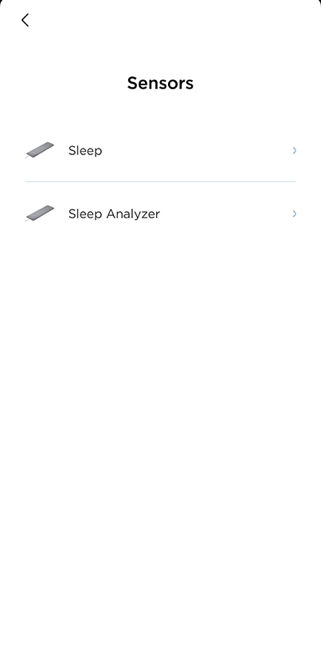


1. Tap Install
2. Place Sleep entirely under your mattress according to the provided instructions. You can also place it between the mattress and mattress topper/pad.


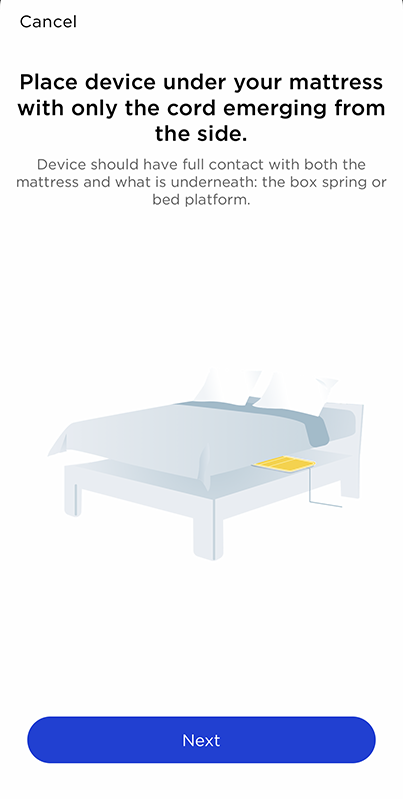

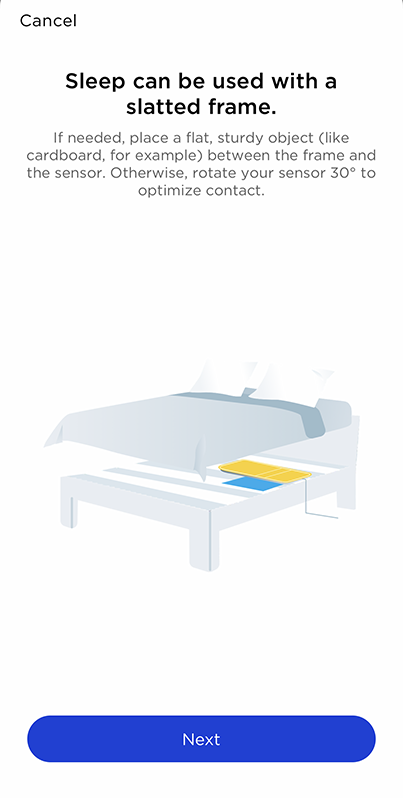

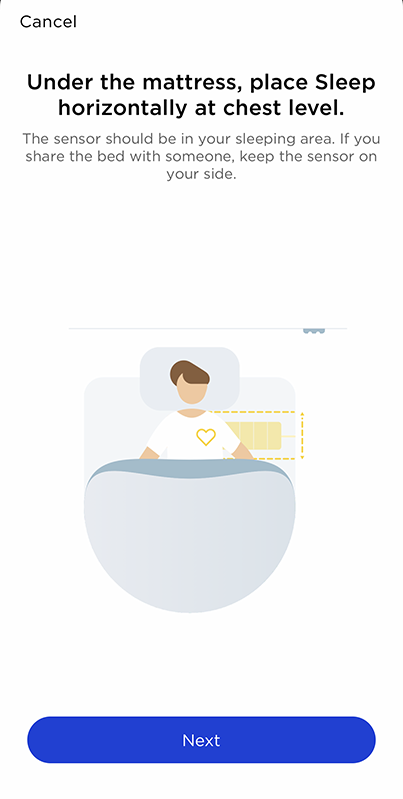


1. Plug Sleep using the provided adapter. Please keep the device plugged in throughout the duration of the study.


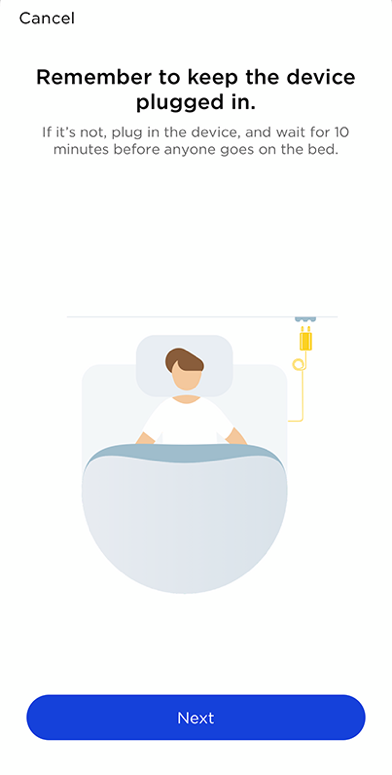


1. Pair the sensor to the iPhone by tapping Pair as requested by the app.


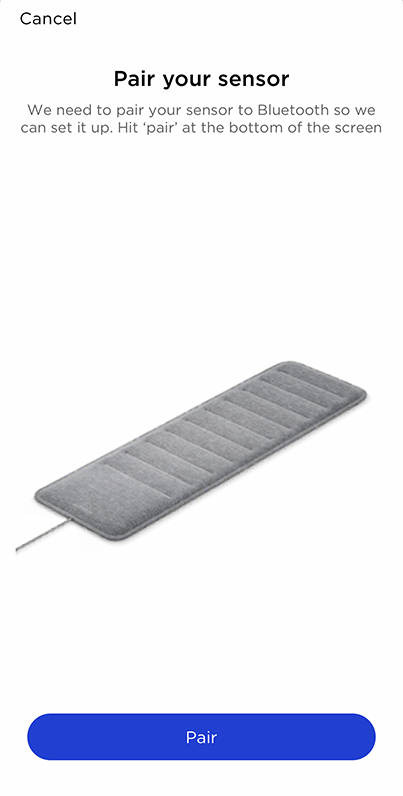

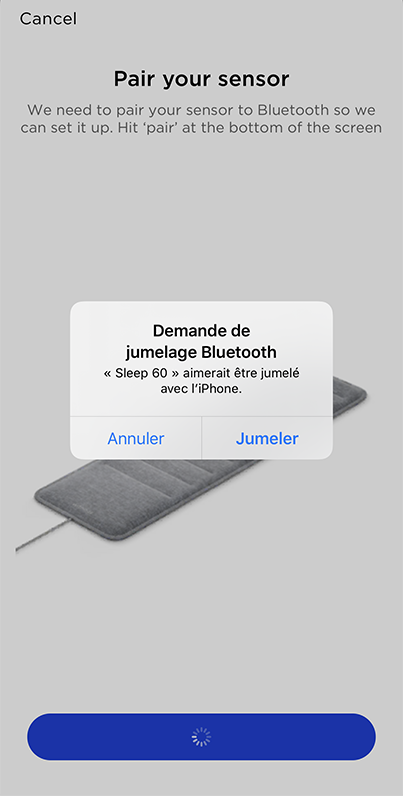


1. Tap the Wi-Fi network you want to use or tap Choose a different network. Please select the network that is most stable and with better connectivity.


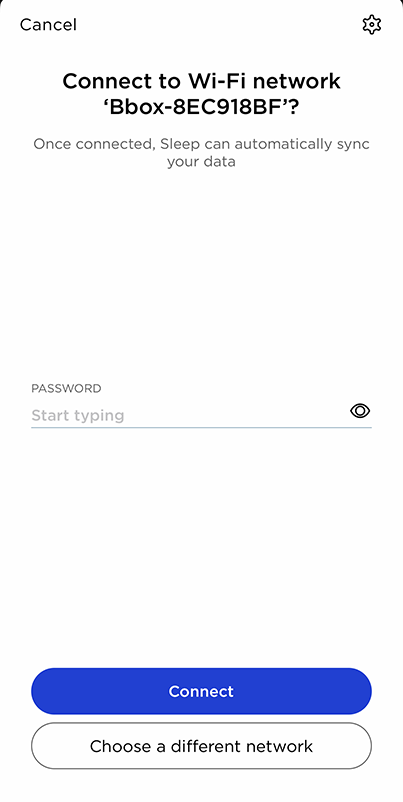


1. Tap Next for the calibration of your Sleep to start. This step can last up to 10 minutes during which a buzzing sound can be heard. Please do not sit on the bed during the process. You will receive a notification in the Timeline of the Health Mate app once the calibration process is over.


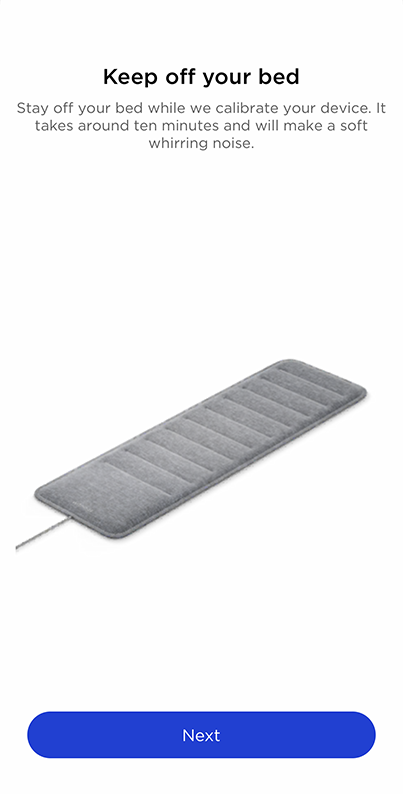


1. If the calibration process went well, you can start using the Sleep Sensor! Thank you for following the instructions.
2. Please return the device inflated to the researcher at the end of the study.

Adapted from: <https://support.withings.com/hc/en-us/articles/360020911714-Sleep-Sleep-Analyzer-User-Guide>

1. **Withings BPM Connect – Get Started**

**Charging** BPM Connect lasts about 6 months per charge, so you shouldn’t need to charge it during the study. However, if the battery of the device is low, you can charge your BPM Connect using the charging cable. To do so, connect the USB end of the charging cable to a power source. Please charge it for approximately 3 hours.


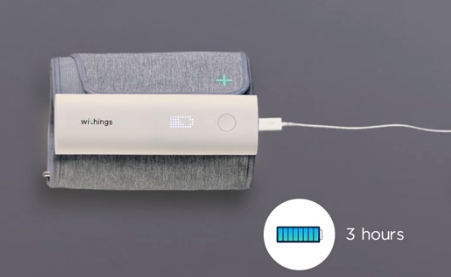


Adapted from: <https://support.withings.com/hc/en-us/articles/360024569473-BPM-Connect-Charging-the-device>

1. **Empatica E4 – Get Started**

The Empatica E4 comes with pre-installed silver-plated electrodes (1), a USB dock placed under the device (2), and a USB MICRO-B Cable (4).


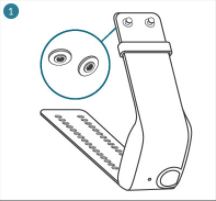

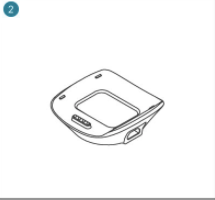

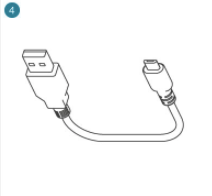


The E4 is easy to wear and adjust. To ensure proper fit and quality data, please follow the steps below:

1. Slide the loop towards the case and place the E4 wristband top-down on a surface.
2. Wear the E4 wristband on the non-dominant hand with the case on the top of the wrist. The EDA electrodes (under the snap-fastener) should line up on the bottom of the wrist. Line them up under the middle and ring fingers.


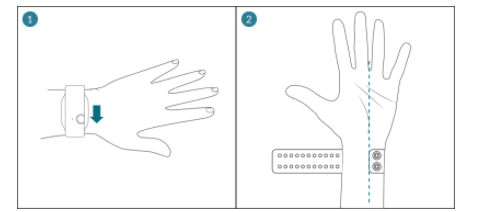


1. Wrap the band over snaps and tighten. To secure, connect one snap at a time. If too tight, loosen by one snap. Tighten the E4 wristband band enough to ensure the EDA electrodes do not change position on the skin during normal movement but not so much as to constrict blood flow or cause discomfort. Adjust the band by sliding up the wrist towards the elbow until it is snug. Reposition the band if it becomes loose during use.
2. The E4 wristband should fit snugly above the wrist joint. When the E4 wristband is properly secured, you should not be able to see any light escaping from the PPG sensor on the back of the wrist under the E4 wristband without lifting the housing from the wrist.
3. Press the button for 2 seconds to power on the E4 wristband.


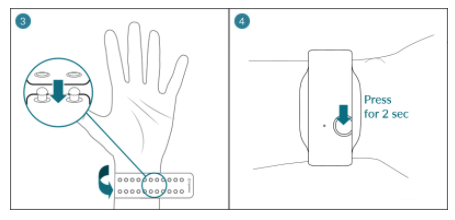


Adapted from: <https://www.empatica.com/get-started-e4>

**Charging** In order to charge the device please follow the instructions below.

- Snap the E4 into the dock and affix the dock via USB to a power source.
- The LED will turn YELLOW indicating it has received power and is charging.
- When E4 is fully charged, the LED will turn a solid green.

The charging dock is only used while charging the E4; **remove it before wearing the device**. It is a passive component. Charging typically takes between 1 and 2 hours.


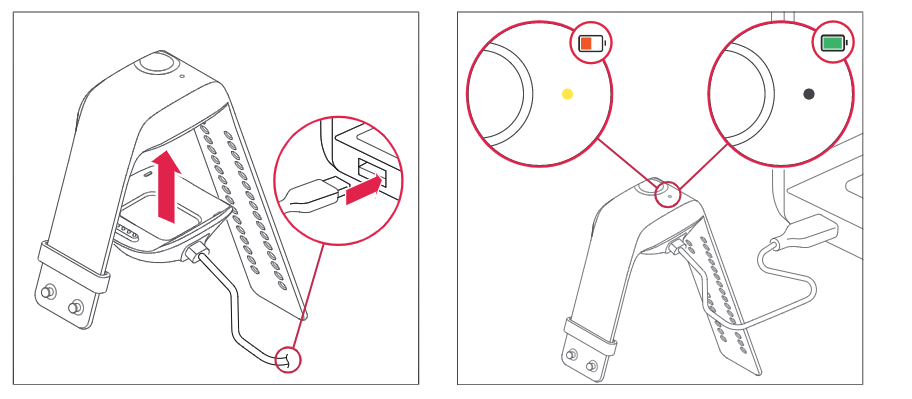


**Data Collection Schedule**

Welcome and thank you for participating in our study and contributing to our research on the use of wearable devices to improve population health and stress detection. If you haven’t done so, we ask that you first read the **User Manual – Get Started** document.

If you already read the previous document, we ask that you please take a moment to read the following instructions to ensure you properly follow the schedule for data collection.

Please take a moment to look at the schedule below.


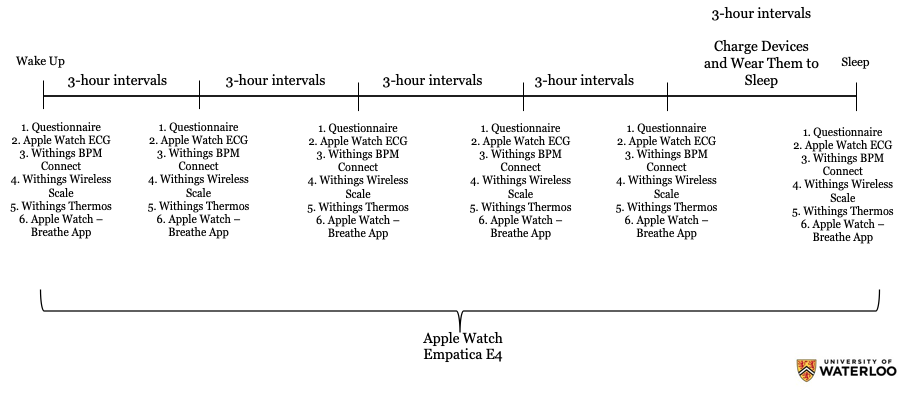


Throughout the study, we kindly ask that you **wear the Empatica E4 and the Apple Watch throughout the day and night**, taking the devices at the end of day for charging as detailed in the diagram and **wearing them again to bed.** Further, we ask that you leave the **Withings Sleep device plugged** for the duration of the study.

Obs: if the Empatica device is too far away from the phone, the session disconnects. We ask that you please check throughout the day in the Empatica E4 Realtime App
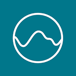
 to ensure data collection is still ongoing. If not, please start recording a new session as soon as possible.

We also ask that you perform the data collection protocols, detailed in the document **User Manual – Data Collection Protocol**, 6 times during the day. Please try taking the measurements approximately every 3 hours. Taking the measurement before or after the 3 hours shouldn’t be a problem – the important is to take the measurements 6 times during the day according to intervals as regular as possible. If you miss one data collection, please take the readings as soon as possible.

The order for data collection is as follows:

1. Fill the **Stress Questionnaire**.
2. Take an **Apple Watch ECG** Reading.
3. Take a Blood Pressure reading with the **Withings BPM Connect**.
4. Take a Weight reading with the **Withings Wireless Scale** (obs: For the scale, please take the readings every moment that you are at home; it is not expected that you will carry the scale with you throughout the day).
5. Take a Temperature reading with the **Withings Thermos.**
6. Take an Apple Watch Mindfulness app 5-minute reading.

Items 2, 3,4 and 5 can be done in any order. However, please fill out the **Stress Questionnaire** at the beginning of the cycle and use the **Apple Watch Breathe App** as the last measure in the cycle.

**After data collection:**

Once you are done with all the readings, please fully close the Apple Health
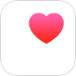
and Health Mate
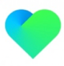
 app, open it again and wait until it syncs the new data.

**Obs:** To fully close an app in the iPhone, double click on the Home Button (the circle beneath the screen). You will see all open apps. Swipe up to close them.


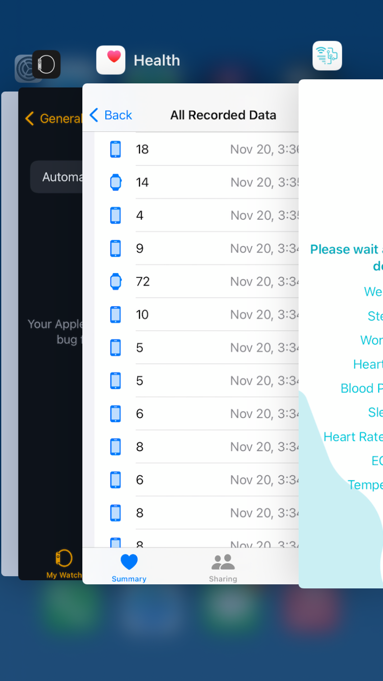


**Data Collection Protocol**

Welcome and thank you for participating in our study and contributing to our research on the use of wearable devices to improve population health and stress detection. If you haven’t done so, we ask that you first read the **User Manual – Get Started** document.

If you already read the previous document, we ask that you please take a moment to read the following instructions to ensure you know how to properly use the devices and apps and collect data for the study.

Before taking the readings, please ensure that you are wearing your Apple Watch device on your dominant hand and the Empatica E4 on your non-dominant hand throughout the day.

1. **Empatica E4**

At the start of the day, please wear your Empatica E4 as described in the **User Manual – Get Started Document.** In order to begin taking the reading:

- Launch the E4 realtime App
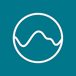

- Power ON the E4 - when the device is powered off a 2-sec button press will power it on. The LED indicator will blink light blue.


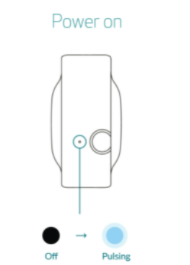


- Start streaming - tap “CONNECT E4 AND START STREAMING” and select your E4 from the list. The LED light turns a steady blue to indicate that streaming has started. In a few second the real-time streaming starts.


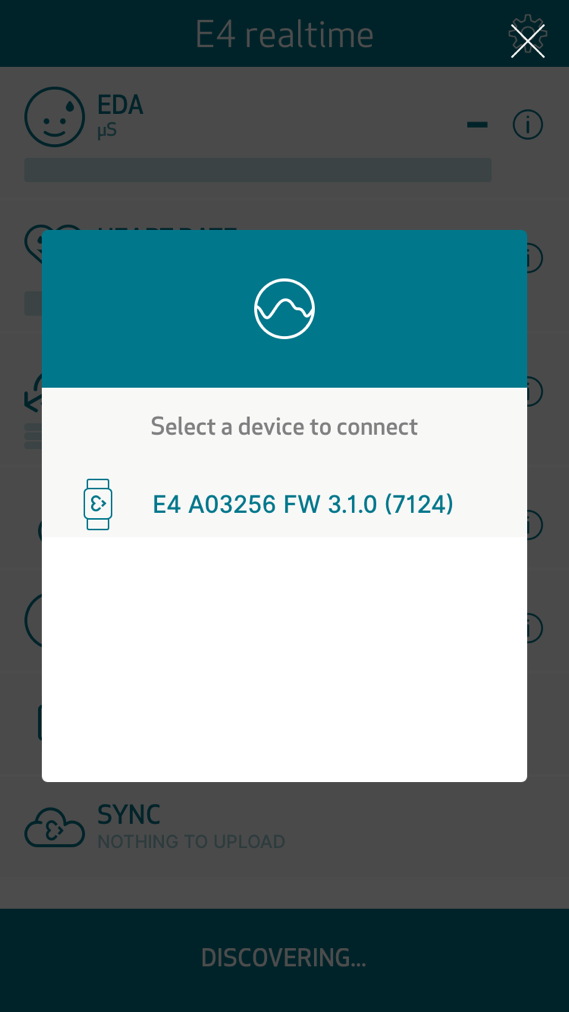


- **For the Empatica E4 device, you only need to start recording once throughout the day.**
- End session - press the "STOP RECORDING" button on the home screen. The E4 powers off and the session uploads automatically to Empatica secure cloud storage. The E4 will also power off if the Bluetooth connection is lost. e.g. is out-of-range. Please only end the session at the end of day or and charge the device.
- **In occasions where the device should be removed (e.g., shower), please end the session and start it again once you wear the device.**

1. **Stress Questionnaire**

Important: please ensure that the phone is connected to Wi-Fi before taking the stress questionnaire.

1. Please access the MHP app.
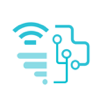

2. Please click on the tab **Questions (the second tab in the bottom)**


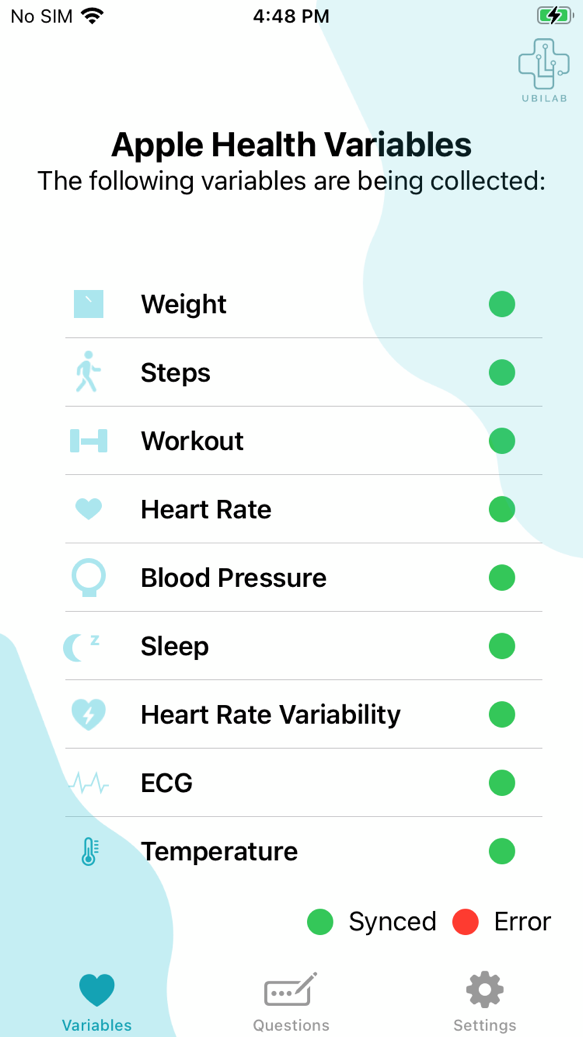


1. Please click on the **Fill Questionnaire** button


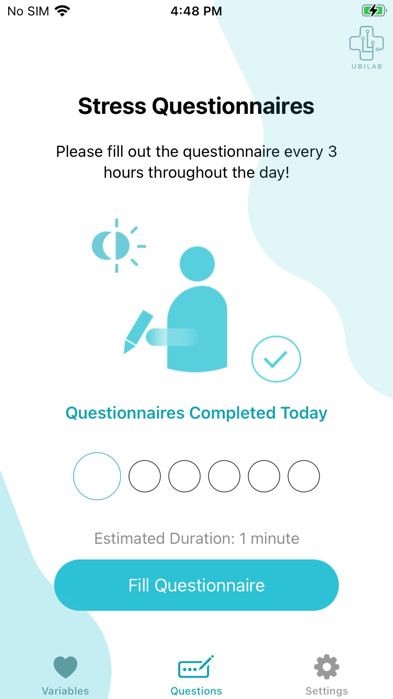


1. Please complete the form to the best of your abilities. After you respond to a question, click rhe right arrow to move on to the next one.

**
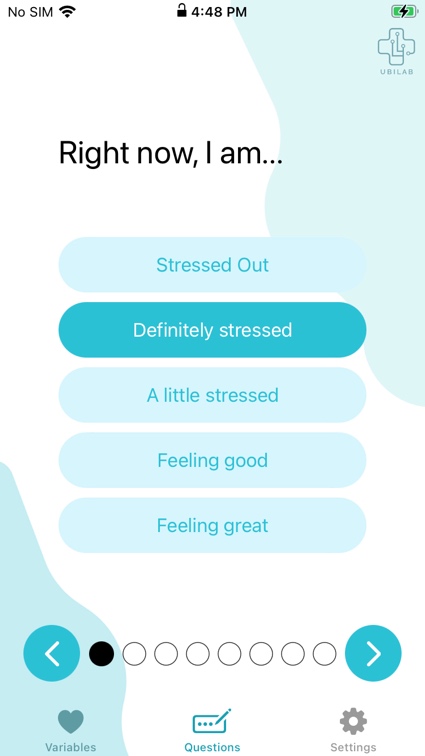
**

1. Once all questions are responded, the screen below will appear. Please wait until the **Updating**…message disappears. Please wait on the tab for a few seconds until you are redirected to the first questionnaire screen.

**
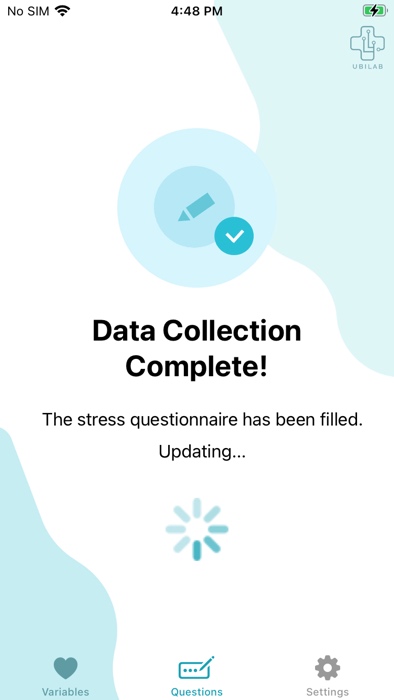

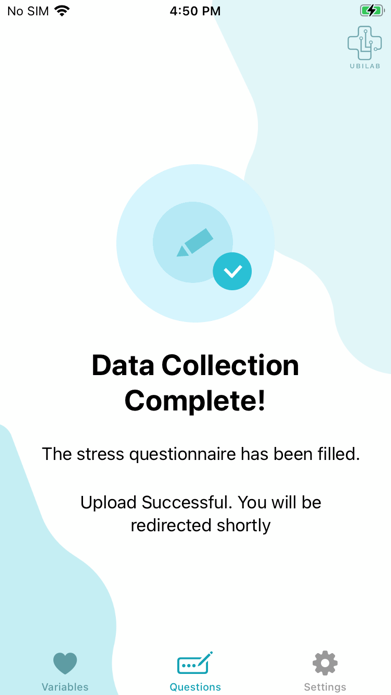
**

1. **Apple Watch - ECG**

Preparing for taking the reading:

- Rest your arms on a table or in your lap while you take a recording. Try to relax and not move too much.
- Make sure that your Apple Watch isn’t loose on your wrist. The band should be snug, and the back of your Apple Watch needs to be touching your wrist.
- Make sure that your wrist and your Apple Watch are clean and dry.
- Make sure that your Apple Watch is on the wrist that you selected in the Apple Watch app. To check, open the Apple Watch app, tap the My Watch tab, then go to General > Watch Orientation.
- Move away from any electronics that are plugged into an outlet to avoid electrical interference.

Please follow the instructions below when taking an ECG reading:

1. Make sure that your Apple Watch is snug and on the wrist that you selected in the Apple Watch app. To check, open the Apple Watch app, tap the My Watch tab, then go to General > Watch Orientation.
2. Open the ECG app 
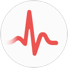
 on your Apple Watch.
3. Rest your arms on a table or in your lap.
4. With the hand opposite your watch, hold your finger on the Digital Crown. You don't need to press the Digital Crown during the session.
5. Wait. The recording takes 30 seconds.


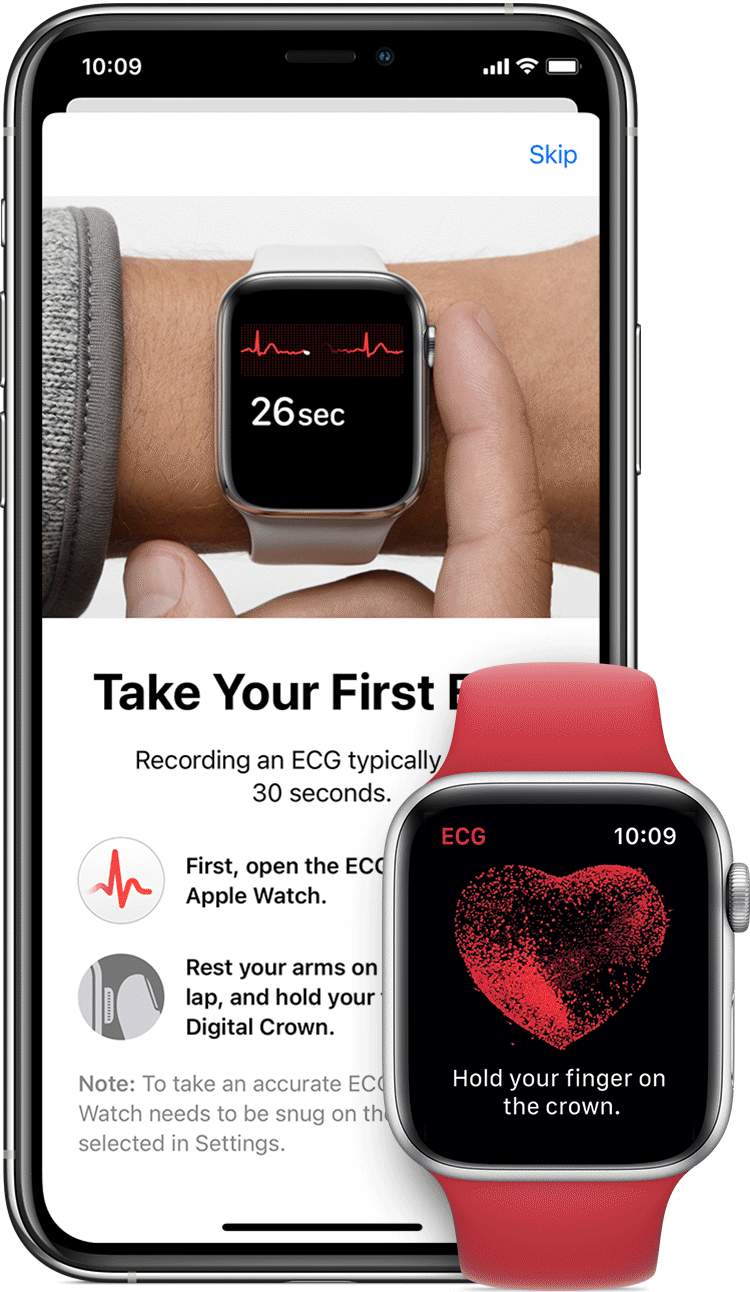


**Please keep in mind that the Apple Watch cannot: detect a heart attack; blood clots or stroke; other heart-related conditions. If you are not feeling well, please contact emergency services.**

Adapted from: <https://support.apple.com/en-ca/HT208955>

1. **Withings BPM Connect**

Preparing for taking the reading:

- Use BPM Connect on the left upper arm
- Rest 5 minutes before the measurement.
- Sit down in a comfortable position, legs uncrossed, feet flat or on the floor, arm and back supported.
- Do not speak or move during the measurement.
- You can wear one layer of clothes but it should not cover your left arm.
- The electrodes should be in contact with the skin.
- Take the measurement in a calm and quiet area.


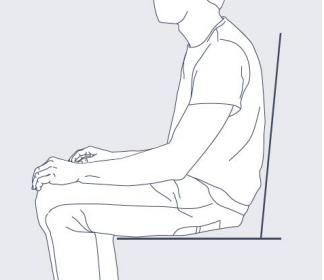


Please follow the instructions below when taking a Blood Pressure reading:

- Unroll cuff and place your arm inside it.


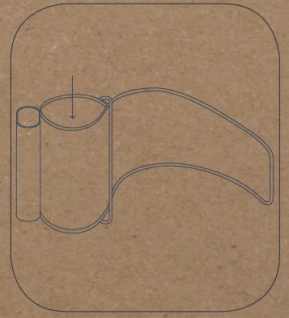


- Tighten the cuff around your arm. The tube should be positioned against your inner arm.


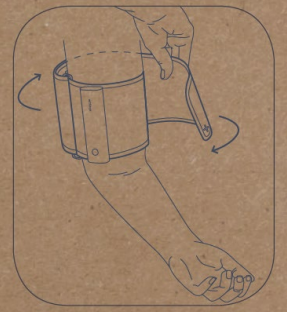


- Place your arm on a table and level with your heart.


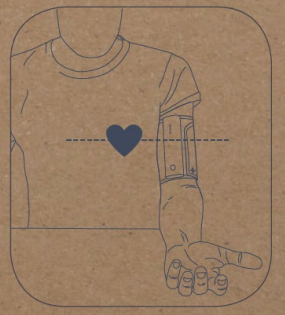


- Press the button to start BPM Connect. Press the button again to start the measurement.


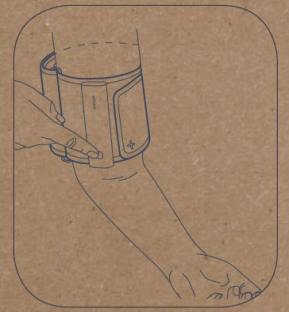


- At the end of the measurement, results are displayed on the screen of BPM Connect. Press the button to validate the measurement. Press the button again to attribute the measurement. Results are sent via WiFi or Bluetooth in the HealthMate app. Please ensure the results were updated.


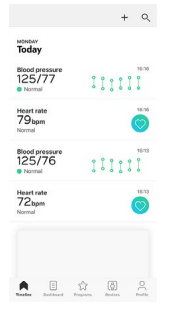


Adapted from: <https://support.withings.com/hc/en-us/articles/360026536033-BPM-Connect-User-Guide>

1. **Withings Wireless Scale**

Taking the reading:

- Step on the scale. Reading should start automatically.
- Adjust your body position according to the arrows that appear on the scale if necessary until the number shown as your weight starts blinking.

1. **Withings Thermos**

- Remove the protective cap (green cap at the bottom of the thermometer).
- Put the device in front of the forehead. The device does not need to touch the skin but it must be close.
- Starting from the center of the forehead, press and release the button of your Thermo and scan across the forehead in a straight line to the temple.


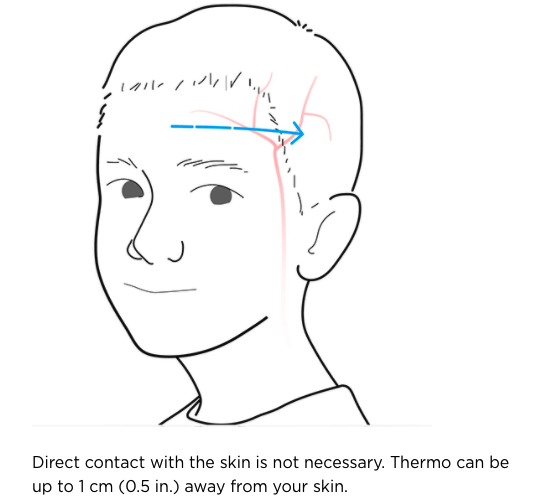


- Thermos vibrates at the end of the measurement and the result comes up on the display. If the result is RETRY, please take another measurement with the device closer to the forehead and moving more slowly, until the result comes up as your current temperature.
- Once the data is read, please assign the correct user. You can do this by sliding your finger up or down on the touch sensitive area of the display to select the correct user, and pressing the button to confirm your choice.
- Pleae open the **Thermos** app.
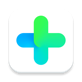

- If there is a blue banner at the top of the screen:


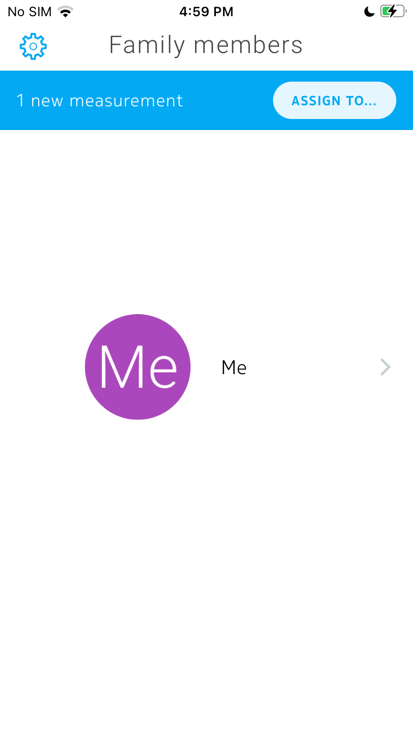


- Click on the Assign to button, and click **Me.**


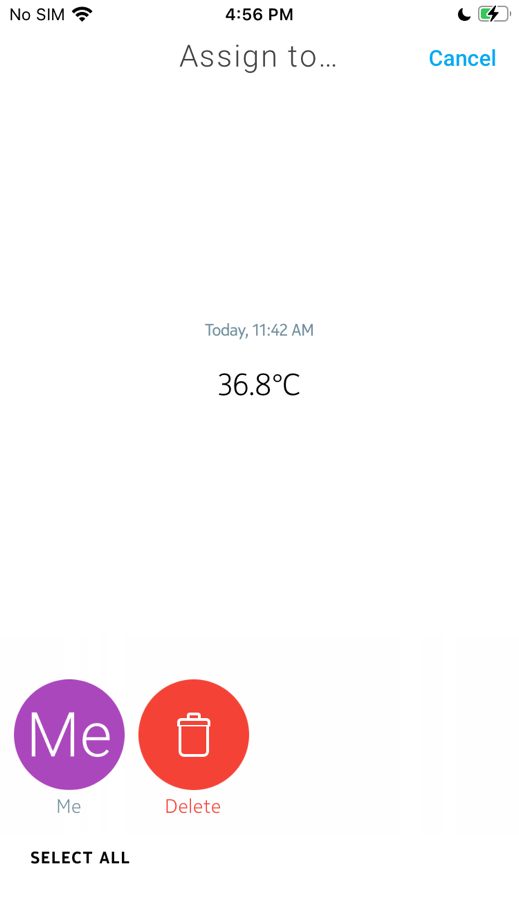


- The new measurement should appear on top of the timeline.


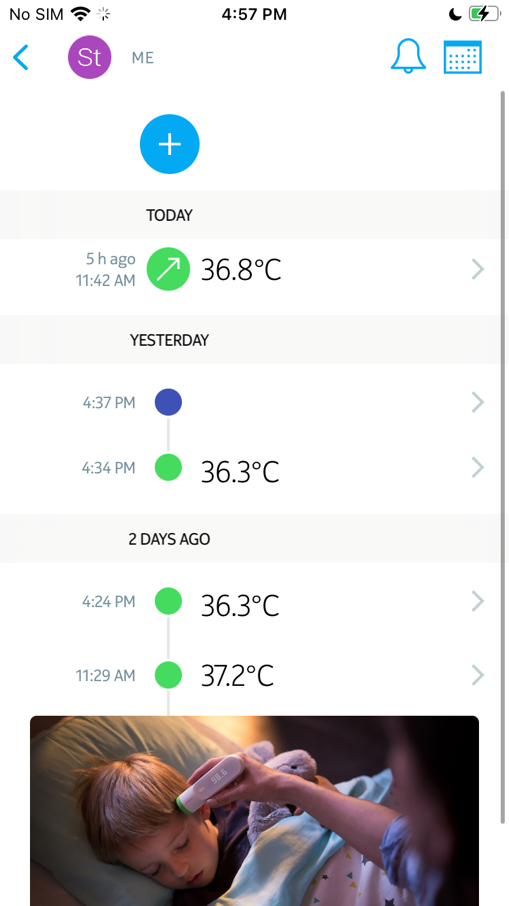


1. **Apple Watch – Breathe/Mindfulness App**

**Please remember to take this reading as your last reading of the cycle, after blood pressure, ECG, and stress questionnaire.**

Taking a reading:

- On the Apple Watch, access the Mindfulness app
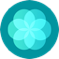

- Press the Digital Crown to go to the Home screen, then open the Mindfulness app.
- The session length should be set for 5 minutes to ensure data accuracy. In case the session length is different, please turn the Digital Crown to set the session's length to 5 minutes.
- Tap Start when you're ready. Remember to stay still while you breathe.
- Inhale as the animation grows and your watch taps your wrist. Then exhale as the animation shrinks and the taps stop.
- Breathe until the session ends and your watch taps you twice and chimes. When you're done, you can see your heart rate.
- When you use the Mindfulness app, your watch mutes some notifications, so you can focus. If you answer a call or move too much during a session, the session ends automatically, and you won’t get credit.

Adapted from: <https://support.apple.com/en-ca/HT206999>

1. **Apple Watch – Workout App**
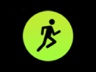


When you begin a workout, please use the **workout** app on the Apple Watch.

1. Open the Workout app.
2. Find the workout that best matches your activity and select it.
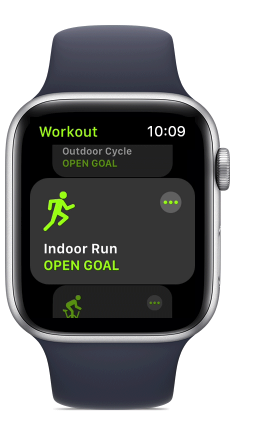

3. To end your workout, swipe right, then tap the End Button.
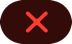
.


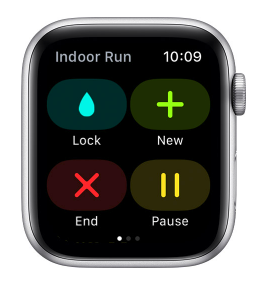


1. **Withings Sleep**

- After setup as described in **User Manual – Get Started**, Withings Sleep will collect data automatically when plugged. Please keep the device plugged for the duration of the study.
- In the morning, please verify in the Health Mate app that sleep data was collected correctly.

*
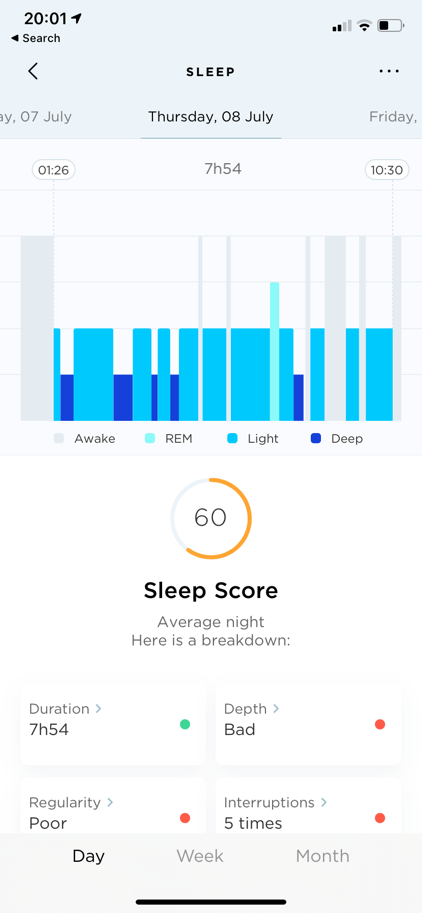
*
